# Molecular Tools for the Study of ADP‐Ribosylation: A Unified and Versatile Method to Synthesise Native Mono‐ADP‐Ribosylated Peptides

**DOI:** 10.1002/chem.202100337

**Published:** 2021-05-06

**Authors:** Jim Voorneveld, Johannes Gregor Matthias Rack, Luke van Gijlswijk, Nico J. Meeuwenoord, Qiang Liu, Herman S. Overkleeft, Gijsbert A. van der Marel, Ivan Ahel, Dmitri V. Filippov

**Affiliations:** ^1^ Leiden Institute of Chemistry Leiden University Department of Bioorganic Synthesis Einsteinweg 55 2333CC Leiden The Netherlands; ^2^ Sir William Dunn School of Pathology University of Oxford South Parks Road Oxford OX1 3RE United Kingdom

**Keywords:** ADP-ribosylation, (ADP-ribosyl) hydrolase, poly(ADP-ribose) polymerases, post-translational modification, solid-phase peptide synthesis

## Abstract

ADP‐ribosylation (ADPr), as a post‐translational modification, plays a crucial role in DNA‐repair, immunity and many other cellular and physiological processes. Serine is the main acceptor for ADPr in DNA damage response, whereas the physiological impact of less common ADPr‐modifications of cysteine and threonine side chains is less clear. Generally, gaining molecular insights into ADPr recognition and turn‐over is hampered by the availability of homogeneous, ADP‐ribosylated material, such as mono‐ADP‐ribosylated (MARylated) peptides. Here, a new and efficient solid‐phase strategy for the synthesis of Ser‐, Thr‐ and Cys‐MARylated peptides is described. ADP‐ribosylated cysteine, apart from being a native post‐translational modification in its own right, proved to be suitable as a stabile bioisostere for ADP‐ribosylated serine making it a useful tool to further biochemical research on serine ADP‐ribosylation. In addition, it was discovered that the *Streptococcus pyogenes* encoded protein, *Spy*MacroD, acts as a Cys‐(ADP‐ribosyl) hydrolase.

## Graphical abstract

**ADP‐ribosylated peptides**: A universal approach to synthesise mono‐ADP‐ribosylated peptides is presented. These peptides can provide a valuable tool for studying ADP‐ribosylation. This is demonstrated by the synthesis of a variety of ADP‐ribosylated peptides modified on Ser‐, Thr‐ or Cys‐residues, the latter of which can act as a stable isostere for Ser‐ADPr as Cys‐ADPr is not reversed by the Ser‐ADPr hydrolase ARH3. Furthermore, in using these peptides, the first hydrolase capable of hydrolysing Cys‐ADPr was identified.

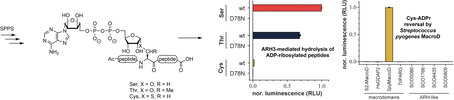

## Introduction

ADP‐ribosylation is a complex post‐translational modification (PTM) that partakes in a wide variety of cellular and physiological biology, including DNA damage response (DDR) and immune‐related processes. ADP‐ribosylation occurs by transfer of ADP‐ribose from NAD^+^ onto one of multiple types of amino acids including ones featuring a carboxylic acid (Glu/Asp),[^1^, ^2^, ^3^, ^4^] an alcohol (Ser/Tyr),[^5^, ^6^] a thiol (Cys) or a guanidine (Arg).[^7^, ^8^, ^9^, ^10^, ^11^] The ADPr modification of amino acid side chains of proteins is catalysed by members of the (ADP‐ribosyl)transferase (ART) superfamily, including poly(ADP‐ribose) polymerases (PARPs).^[10]^ In the past few years, many insights have been acquired regarding the biological role of ADP‐ribosylated serine (Ser‐ADPr),^[8]^ its chemical stability,^[12]^ and the distinctly different mechanisms by which Ser‐ADPr is introduced (by PARP1:HPF1 complex)[^13^, ^14^, ^15^] and removed (by (ADP‐ribosyl)hydrolase 3 (ARH3)).^[16]^ Cysteine ADPr has been shown to be synthesized by PARP7^[17]^ (TiPARP) and PARP8, but hydrolases for erasing this particular PTM remain unidentified to date.[^7^, ^18^] Herein we describe the development of a new strategy to synthesize ADP‐ribosylated peptides, modified on the side chains of serine (Ser), threonine (Thr) and cysteine (Cys) residues. We reasoned that ADPr‐Cys containing peptides, besides emulating natural ADP‐ribosylated proteins,[^6^, ^7^, ^18^] may also serve as chemically stable ADP‐ribosylated Ser linkages, possibly less susceptible to ARH3‐catalyzed hydrolysis. Thr‐ADPr is a less common PTM and until now, no family member of mammalian PARP has been identified as being able to transfer ADPr onto threonine. Reports have emerged however that some bacterial enzymes are able to ADP‐ribosylate threonine residues, for example, on human ubiquitin (Ub).^[19]^ This modification inhibits the function of PolyUb on multiple levels, such as biosynthesis, Ub recognition and the reversal of the modification, and plays a crucial role in bacterial colonization.

In 2016, we developed the first solid phase synthesis of ADP‐ribosylated peptides, in which a phosphoribosylated building block was used for the introduction of ribosylated amino acids in a peptide sequence.^[20]^ The ensuing installation of the pyrophosphate moiety forced us to use acid labile *t*Bu‐protection of the phosphotriester to attain orthogonality. After removal of the *t*Bu‐groups, the ADP moiety was introduced via our established P^III^−P^V^ coupling method^[21]^ with an adenosine phosphoramidite. Although a variety of sequences, MARylated on Gln, Asn or Cit sites^[20]^ as well as Ser^[22]^ have been prepared using this method, inherent drawbacks of the methodology include the presence of a carboxamide at the C‐terminus of the synthetic MARylated peptide and the extensive amount of protective group manipulations that is needed for some peptide sequences. For instance, when the ADPr‐modification site in the target peptide is flanked by Ser or Thr at the *C*‐terminal or N‐terminal sequence, the side chain protecting trityl (Trt) groups must be replaced by an acetyl to allow further processing of the *t*Bu‐protected phosphotriester.

**Scheme 1:**
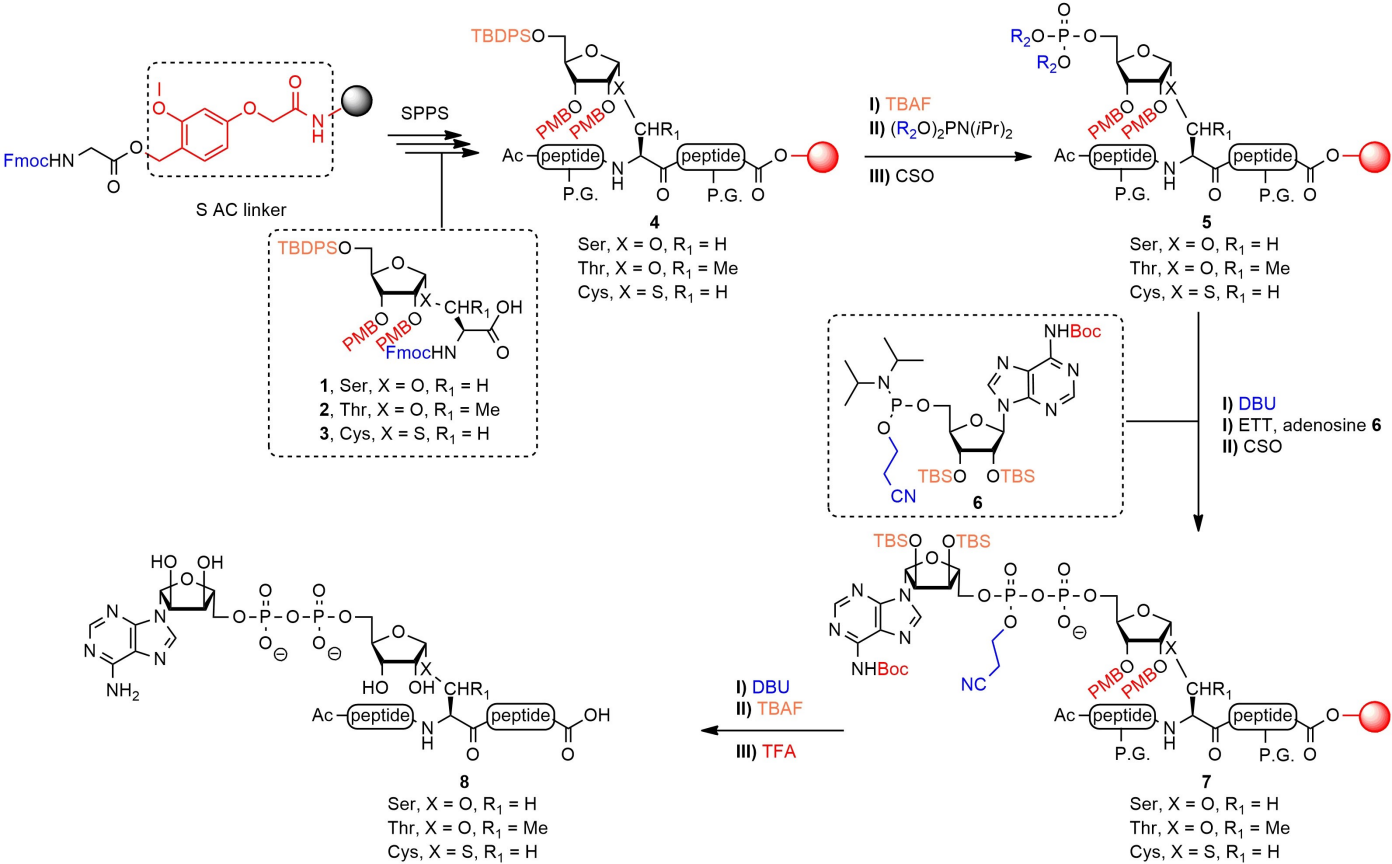
Synthetic strategy for the preparation of peptides MARylated on their Ser‐, Thr‐ or Cys‐residue. P.G.=protecting group for amino acid side chains: Trt for serine/threonine/histidine, Mtt for lysine, 2‐Ph*i*Pr for glutamic acid, bis‐Alloc for arginine.

The here‐presented new, generally applicable strategy toward MARylated peptides overcomes these disadvantages. In the design of our new method we decided to again employ Fmoc‐based solid‐phase peptide synthesis (SPPS) that is compatible with most peptide synthesizers and also to our procedure for the introduction of the pyrophosphate moiety. We selected the TentaGel^®^ resin equipped with the highly acid sensitive S AC linker^[23]^ (Scheme 1), because this would return oligopeptides with a C‐terminal carboxylic acid, thus to ADPr‐peptides more closely resembling natural sequences, such as those that would emerge from proteolytic processing of ADP‐ribosylated proteins. The acid‐sensitive Trt and 4‐methyltrityl (Mtt) groups were chosen for protection of the side chains of Ser‐, Thr‐ and Lys‐residues. We decided to postpone the introduction of the phosphotriester to the final stage of the SPPS leading to the design of ribosylated amino acid building blocks **1**–**3** (Scheme 1) for incorporation of the prospected ADPr moiety in the sequence. The building blocks are compatible with standard Fmoc‐based SPPS and are endowed with the following protecting groups on the ribosyl moiety: **I**) the 4‐methoxybenzyl (PMB) groups on the secondary hydroxyls, which proved useful for orthogonal protection in ribosides^[24]^ and pyranosides,^[25]^ enabling α‐selective glycosylation and also cleavage in the final stage of the immobilized MARylated peptides, and **II**) the bulky TBDPS group which not only enhances α‐selectivity during glycosylation, but also provides orthogonality for the introduction of the phosphotriester, the first step in the installation of the ADP‐moiety. For phosphotriester introduction, reagent **20** with fluorenylmethyl (Fm) groups^[26]^ and reagent **21** equipped with 2‐methylsulfonylethyl (Mse) groups[^27^, ^28^] were chosen (Scheme 4), as both of these can be cleaved in an orthogonal fashion by treatment with DBU. With these changes of the solid phase procedure and the protecting group pattern of the newly designed ribosylated amino acid building blocks, adenosine amidite **6** was chosen to complete the ADPr installation, after which global deprotection by subsequent treatment with DBU, TBAF and TFA would furnish the MARylated peptides. We have utilized this new SPPS approach to assemble a selection of biologically relevant MARylated peptides. With these, we investigated the influence of the ADP‐ribosylated amino acid on signal turn‐over by a variety of human and microbial (ADP‐ribosyl)hydrolases. We found that the Ser>Thr exchange slowed the hydrolysis reaction by ARH3, whereas Ser>Cys replacement abolished the reaction thus indicating that the Cys‐MARylated peptide is a useful stabilized bioisostere for the study of human proteins. As well, we found that *Spy*MacroD, a macrodomain‐type enzyme from *Streptococcus pyogenes*, efficiently removed the Cys modification.

## Results and Discussion

**Scheme 2:**
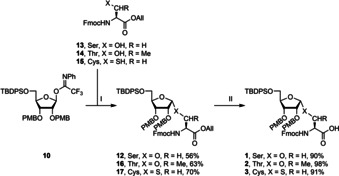
Synthesis of Fmoc‐based SPPS building blocks **1**–**3**. Reagents and conditions: **I)** TBSOTf, acceptors **13**, **14** or **15**, DCM, −50 °C. **II)** Pd(PPh_3_)_4_, DMBA, DCM. All=allyl.

The glycosylation procedure toward the ribofuranosylated Fmoc‐amino acids **1**–**3** was first optimized by testing two ribosyl donors; the known *N*‐(phenyl)trifluoroacetimidate donor **10** ^[29]^ and its trichloroacetimidate^[30]^ congener **9** (Table 1, see Supporting Information for preparation). The latter was chosen for its synthetic accessibility as donor **10** requires the labour‐intensive synthesis of 2,2,2‐trifluoro‐*N*‐phenyl‐acetimidoyl chloride as a reagent. On the other hand, trichloroacetimidate donors can undergo a Chapman‐like rearrangement to form the unreactive glycosylamides under glycosylation conditions.[^31^, ^32^] As a model reaction for the glycosylation, the condensation of serine acceptor **13** (Scheme 2, see Supporting Information for preparation) with ribosyl donors **9** and **10** was examined by varying the reaction conditions in terms of temperature, concentration and nature of the activator, the results of which are listed in Table 1. Coupling of donor **9** with acceptor **13** using the same conditions as described before in a similar reaction^[12]^ gave a low yield of the wanted ribofuranosylated Fmoc‐Ser **12** together with a significant amount of side‐product **11**, originating from the Chapman‐type rearrangement, (entry 1).[^32^, ^33^] Changing the activator to TfOH (entry 2) led to acid‐catalyzed cleavage of one or more PMB‐protecting groups and the PMB‐cation was scavenged by the acceptor, resulting in Fmoc‐Ser(PMB)‐OAll together with a complex mixture of ribose derived products. In an attempt to reduce the loss of the PMB group, TBSOTf was used as an activator (entry 3). It was reasoned that the softer character of the TBS cation would decrease the acidity of the glycosylation conditions thus diminishing the cleavage of the PMB groups. Although the use of TBSOTf significantly improved the yield of product **12**, side product **11** still occurred in a 12 % yield. A two‐fold increase in the concentration of donor **9** unexpectedly enlarged the formation of side product **11** to 48 % (entry 4). Finally, increasing the temperature of the reaction mixture to −40 °C did improve the yield of the glycosylation to 60 % (entry 5). For donor **10**, the same set of reaction conditions were tested (entries 6–10). It is of interest to note that using TfOH as activator with donor **10**, the amount of acid‐catalyzed PMB ether cleavage has been significantly reduced in comparison with trichloroacetimidate counterpart **9**. However, the results of the glycosylations proved to be more reproducible with TBSOTf, particularly for scaling up the reaction.

**Table 1:** Optimization of the glycosylation conditions with donors **9** and **10** and acceptor **13**. The concentration (C) is the concentration of the donor in DCM as solvent. Activators were used in 0.1 equivalent relative to the donor and the reactions were carried out at 0.2 mmol scale.

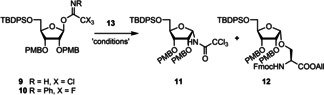						
entry	donor	C (M)	T (°C)	activator	11 (%)	12 (%)
1	**9**	0.1	−50	TMSOTf	34	23
2	**9**	0.1	−50	TfOH	n.d.	0
3	**9**	0.1	−50	TBSOTf	12	39
4	**9**	0.2	−50	TBSOTf	48	17
5	**9**	0.1	−40	TBSOTf	12	60
6	**10**	0.1	−50	TMSOTf	–	32
7	**10**	0.1	−50	TfOH	–	53
8	**10**	0.1	−50	TBSOTf	–	56
9	**10**	0.2	−50	TBSOTf	–	23
10	**10**	0.1	−40	TBSOTf	–	48

Having optimized the reaction conditions (entry 8, Table 1) we glycosylated appropriately protected acceptors **13** (Ser),[^34^, ^35^] **14** (Thr)^[34]^ and **15** (Cys) (See Supporting Information for preparation) with ribosyl donor **10**.^[29]^ This transformation furnished the suitably protected, ribofuranosylated amino acids **12**, **16** and **17** in high α‐selectivity (no β‐product was observed) and good yields (56–70 %, Scheme 2). For protection of the C‐terminus, the allyl ester was chosen since it can be selectively removed by treatment with catalytic Pd(PPh_3_)_4_ under neutral conditions.^[36]^ Treatment of amino acids **12**, **16** and **17** with Pd(PPh_3_)_4_ and 1,3‐dimethylbarbituric acid (DMBA) as the allyl cation scavenger^[37]^ furnished the required building blocks **1**, **2** and **3** in good yields in a few steps.

**Scheme 3:**
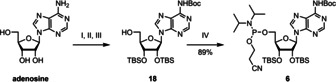
Synthesis of adenosine amidite **6**. Reagents and conditions: **I)** TBS−Cl, imidazole, DMF, 50 °C. **II)** Boc_2_O, DMAP, THF, reflux. **III)** TFA, H_2_O, THF, 0 °C. **IV)** 2‐cyanoethyl‐*N,N*‐diisopropylchlorophosphoramidite, DIPEA, DCM, rt.

The final building block, needed for the assembly of MARylated peptides is adenosine phosphoramidite **6** (Scheme 3) that enables the introduction of the ADPr moiety via the P^III^−P^V^ procedure.^[38]^ Silylation of the hydroxyl functions in adenosine with TBS−Cl was followed by protecting the exocyclic amine with a Boc group. The ensuing acid mediated, regioselective desilylation^[39]^ of the primary alcohol led to partially protected adenosine **18**, which was phosphitylated to give phosphoramidite **6**.

**Scheme 4:**
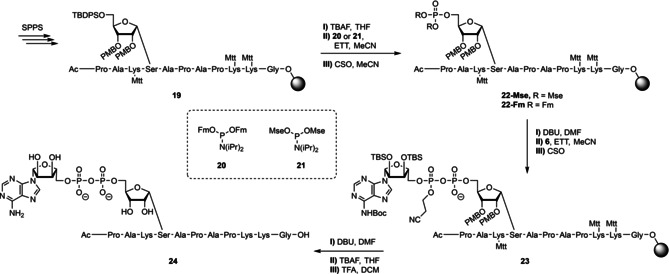
Synthesis of MARylated H2B peptide **24**.

With all the required building blocks in hand, the solid phase assembly of MARylated peptide **24**, derived from the N‐terminus of human histone H2B, was undertaken (Scheme 4). Standard Fmoc solid phase peptide synthesis methodology was employed using amino acid building blocks having highly acid sensitive side chain protecting groups (Mtt for lysine, Trt for serine). As depicted in Scheme 4, Tentagel® S AC resin preloaded with glycine was elongated using the selected protected amino acid building blocks, including ribofuranosylated Fmoc‐Ser‐OH **1** to give immobilized peptide **19**. The ensuing cleavage of the TBDPS‐protecting group was tested using three different F^−^ sources: TEA ⋅ 3HF, HF⋅pyridine and TBAF. Both deprotections using TEA ⋅ 3HF and HF⋅pyridine needed reaction times of up to 16 h to fully remove the silyl protecting group whereas employing a 1 M ⋅ TBAF solution in THF ensured full deprotection in 30 min. The TBAF treatment was not only superior with regard to cleavage time but also in the quality of the product according to LC‐MS analysis of the peptides after desilylation, removal of all other protecting groups and cleavage from the solid support. After desilylation of the primary hydroxyl in the ribose moiety, two phosphoramidite reagents were investigated for obtaining the ribosyl‐5‐phosphomonoester. Two phosphoramidites, both bearing base sensitive protecting groups, were tested: reagent **20** bearing fluorenylmethyl (Fm) groups and reagent **21** equipped with 2‐methylsulfonylethyl (Mse) groups. The phosphitylation of the immobilized peptide, having a ribose with 5‐OH by either the reagent **20** or **21**, followed by the CSO mediated oxidation of the formed phosphite to the phosphotriester intermediate provided the immobilized, fully protected phosphoribosyl peptides **22**‐**Fm** and **22**‐**Mse**, respectively. To convert the phosphotriesters in these into the phosphomonoester, the Fm and Mse protecting groups were removed by treatment of the resins with 10 % DBU in DMF. Monitoring the reaction progress for 20 min by LC‐MS showed that both Fm protecting groups were completely eliminated under these conditions, whereas only one Mse‐group had been removed, leading to the phosphodiester. Although the crystalline Mse reagent **21** is easier to handle, the Fm protecting group was chosen for further synthetic studies for its more efficient deprotection. Thus, the assembly of the MARylated peptide was continued with the deprotection of **22**‐**Fm**. Condensation of the resulting phosphate monoester with adenosine phosphoramidite **6** and oxidation of the resultant P(III)‐P(V) intermediate gave immobilized peptide **23**, containing a partially protected pyrophosphate moiety. The cyanoethyl group was then removed from the pyrophosphate in **23** by 10 % DBU in DMF, after which the silyl ethers were deprotected with TBAF and the remaining protective groups finally removed with concomitant cleavage of the target MARylated peptide from the resin by treatment with 10 % TFA solution in DCM containing 2.5 % TIS as a scavenger. Monitoring of the latter deprotection by LC‐MS analysis revealed that the trityl and PMB‐protecting groups were split off instantly while the more stable Boc protective group on the exocyclic amine of adenosine needed at least 2 h for its removal. Purification with RP‐HPLC of the obtained crude product led to the isolation of homogeneous, MARylated peptide **24**, derived from the N‐terminus histone H2B in 3.6 % overall yield.

Following the above‐described general procedure, we next set out to assemble Ser‐ADPr oligopeptides **25** and **26** (Table 2), both sequences that are confirmed to exist in nature bearing this PTM as established by LC‐MS/MS analysis of biological samples.[^40^, ^41^] The synthesis of peptide **25** proceeded uneventful where **25** was obtained in 11 % after HPLC‐purification. En route to peptide **26**, incorporation of Thr‐7 proved to be difficult and required us to repeat the solid‐phase condensation step employing Fmoc‐Thr(Trt)‐OH twice. Peptide **26** also contains an arginine, for whose side chain no suitable acid labile protecting group is available, which led us to employ bis‐Alloc protection for the guanidine function.[^20^, ^42^] This necessitated adaptation of the deprotection procedure at the final stage of the synthesis of Ser‐ADPr peptide **26** by subjecting the resin to Pd(PPh_3_)_4_ and DMBA as a scavenger prior to treatment with the acidic cleavage cocktail. In this way and after HPLC purification peptide **26** was obtained in 6.1 % overall yield.

**Table 2:** Synthesis of MARylated peptides **24**–**30**. The amino acids indicated by bold print are the modification sites.

Number	Sequence	Yield (%)	Notes
**24**	Ac‐PAKS^ADPr^APAPKKG‐OH	3.6	n.a.
**25**	Ac‐GKS^ADPr^GAALSKKG‐OH	11	n.a.
**26**	Ac‐GKS^ADPr^SGPTSLFAVTVAPPGARG‐OH	6.1	Strenuous coupling of Thr‐7
**27**	Ac‐GKSSGPT^ADPr^SLF‐OH	9.5	n.a.
**28**	Ac‐KEST^ADPr^LHLVLRL‐OH	0.94	n.a.
**29**	Ac‐PAKC^ADPr^APAPKKG‐OH	4.1	EDT added in cleavage cocktail
**30**	biotin‐PAKC^ADPr^APAPKKG‐OH	1.9	*t*BuOOH was used instead of CSO

Having effectively completed the Ser‐ADPr peptides **24**–**26**, our attention was turned to the assembly of ADPr peptides with Thr or Cys at the site of ADP‐ribosylation. Thr‐ADPr peptide **27**, which is sharing part of the sequence of peptide **26**, has been selected to help determine the exact site of modification as its identification by MS/MS of proteomic mixtures is not always conclusive.^[43]^ The aforementioned difficulties incorporating the Thr‐7 residue in **26** were not encountered in coupling ribosylated Thr‐building block **2** to obtain peptide **27**. Another relevant Thr‐ADPr peptide is **28**, a fragment containing the ADP‐ribosylation site in human Ub that is modified at Thr‐66 by the bacterial effector protein CteC, as detected by LC‐MS/MS analysis in proteomics studies.^[19]^ This ADPr peptide includes the amino acids Glu and His and successful construction of this sequence would mean that also these amino acids, in appropriately protected form, can be included in the synthetic scheme. In the SPPS to **28**, which was obtained in 0.94 % after HPLC purification, the building blocks Fmoc‐His(Trt)‐OH and Fmoc‐Glu(O‐2‐Ph*i*Pr)‐OH were used as both protecting groups are removable by the levels of TFA used in the cleavage cocktail. As was mentioned before, peptides containing ADP‐ribosylated cysteine can be considered as isosteric to ADP‐Ser peptides with the ADPr moiety relatively more stabile towards enzymatic hydrolysis. The SPPS of Cys‐ADPr peptide **29**, the Ser‐to‐Cys analogue of **24**, was performed using ribofuranosylated Cys building block **3**. After deprotection and cleavage of the immobilized Cys‐ADPr peptide **29** from the resin, Ac‐PAK**C**(PMB)APAPKKG‐OH was detected, a side product originating from the migration of the PMB cation to the thiol of the Cys side chain.

Addition of ethane dithiol (EDT), a more potent thiol‐based scavenger, to the cleavage cocktail suppressed this side reaction, providing **29** in 4.1 % yield and good homogeneity (see Supporting Information for experimental data such as LC‐MS trace). Lastly, to obtain a useful pull‐down tag for biological experiments, N‐biotin‐Cys‐ADPr peptide **30** was assembled. After completion of the synthesis of **30**, LC‐MS analysis of the crude product revealed a main product with a mass 16 Dalton higher than expected, presumably due to the oxidation of an alkylsulfide. Since such overoxidation has not been detected during the synthesis of the similar Cys‐ADPr peptide **29**, it is postulated that this unwanted reaction has occurred on the sulfur of the biotin tag. Oxidized biotin occurs as both α‐ and β‐sulfoxide and while the β‐form nearly completely ablates its affinity towards avidin the α‐biotin sulfoxide still has strong binding properties^[44]^ and can be used without loss of streptavidin pull‐down efficiency.^[45]^ Still, the synthesis of ADPr‐peptide **30** was repeated, using a milder oxidizing agent than CSO for the oxidation of P(III)−P(V) precursor of the pyrophosphate. Indeed, the application of 0.5 M *t*BuOOH for 30 min proved effective in the chemoselective oxidation of the phosphite species whilst leaving the biotin moiety intact.

**Figure 1:**
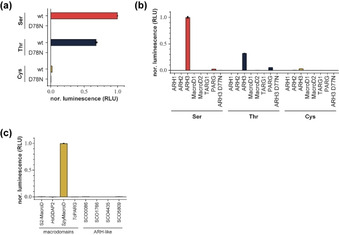
Enzymatic hydrolysis of interglycosidic linkages in ADP‐ribosylated serine, threonine and cysteine containing peptides **24**, **27** and **29**, respectively. (a–c) Measurements of hydrolase activity against the various ADPr‐peptide linkages was assessed by converting released ADPr into AMP via NUDT5 and subsequently measured using the AMP‐Glo™ assay (Promega). Samples are background corrected and normalised to ARH3 wt (a+b) or *Spy*MacroD (c). Data are represented as mean values ± SD measured in triplicates. (a) Hydrolysis of Ser‐, Thr‐ and Cys‐linked ADPr by ARH3 wt and catalytic mutant D77 N. (b) Hydrolysis of Ser‐, Thr‐ and Cys‐linked ADPr by human (ADP‐ribosyl)hydrolases. (c) Hydrolysis of Cys‐linked ADPr by evolutionary diverged (ADP‐ribosyl)hydrolases of the macrodomain and ARH‐like families. Abbreviations: S2‐MacroD, SARS‐CoV‐2 nsp3 macrodomain; *Hs*GDAP2, *Homo sapiens* Ganglioside‐induced differentiation‐associated protein 2 (GDAP2); *Tc*PARG, *Thermomonospora curvata* PARG, *Streptomyces coelicolor* (SCO) ARH‐like hydrolases indicated by their respective gene identifiers.

Having obtained MARylated peptides **24**–**30**, we set out to investigate the differences in enzymatic turn‐over of these modifications. As ARH3 is the only known hydrolase of ADP‐ribosylated serine residues,^[16]^ we first tested its ability to hydrolytically remove the ADPr moiety from these peptides (Figure 1a). We found that ARH3, but not its catalytic mutants D77 N or D78 N, is capable of hydrolysing the glycosidic linkage in **24** (Ser) and **27** (Thr). The turnover of the latter proved not as efficient as the former (Figure 1a and b), which might be a result of steric clash of the additional methyl group of the threonine side chain within the enzyme active site. Please note that we employed 45 min incubation, where we have demonstrated earlier^[12]^ that Ser‐MARylation turn‐over is complete <20 min. In contrast, the ADPr‐Cys interglycosidic linkage was largely stable towards enzymatic hydrolysis under the conditions applied. Since ADP‐ribosylation of cysteine residues is a modification found in human cells, we expanded our investigation to all known human hydrolases and confirmed that only ARH3 could remove the modification from serine and by extension threonine (Figure 1b). Surprisingly, none of the tested hydrolases was able to remove the modification from peptide **26**. This suggests that either the modification is irreversible in human cells or is reversed by a thus far unidentified enzyme. To test whether the modification could in principle be reversed, we tested several evolutionary divergent hydrolases of the macrodomain and (ADP‐ribosyl)hydrolase family from various lower organisms (Figure 1c). While none of the ARH‐like enzymes was able to hydrolyse this particular linkage, *Streptococcus pyogenes* MacroD (*Spy*MacroD)^[46]^ efficiently hydrolysed the ADP‐ribosyl‐cysteinyl glycosidic bond. Earlier structural studies on SAV0325, the *Staphylococcus aureus* homologue of *Spy*MacroD, showed a Zn^2+^‐binding motif within the active site and the authors suggested that this zinc centre participates in substrate recognition and catalysis.^[47]^ Our results suggest that an increased Lewis acidity, due to the presence of the Zn^2+^ ion, relative to other macrodomains as well as production of cysteine, a favourable zinc‐coordination ligand, are supporting the reaction. This result clearly shows that hydrolases can readily evolve into efficient cysteine deADP‐ribosylating enzymes and that such activity may exist in humans. Together, our data provide new insights into the turnover of ADP‐ribosylated substrate and highlight the suitability of the MARylated peptides **24**–**30** as tools for the study of ADP‐ribosylation.

## Conclusion

Herein we describe the development of a new and robust synthetic strategy to obtain peptides modified with mono(ADP‐ribose) at a chosen serine, threonine or cysteine side chain. For this purpose, unprecedented ribofuranosylated Ser‐, Thr‐ and Cys‐building blocks **1**, **2** and **3** were developed and used in Fmoc‐based SPPS to obtain immobilized peptides that were decorated with an orthogonally protected ribosyl unit at the prospected ADPr site. This ribosylated part is then functionalized with a phosphomonoester prior to on‐resin construction of the pyrophosphate, yielding immobilized and partially protected ADP‐ribosylated peptides. The peptides were then subjected to a deprotection sequence, the final acid step of which also led to cleavage from the resin. RP‐HPLC purification of the crude products finally furnished the targeted peptides, MARylated at a predetermined Ser, Thr or Cys residue, in decent yields, homogeneity and sufficient quantities which allows their usage in biological experiments. Our methodology and the set of peptides synthesized vary in functional side chains flanking the ADPr site, demonstrating that a wide set of amino acids can be incorporated in this way. As well, functionalization of the peptide with a biotin tag is allowed, provided that oxidation of the phosphite species is performed with *t*BuOOH rather than CSO.

The availability of the Ser‐ADPr, Thr‐ADPr and Cys‐ADPr peptides allowed us for the first time to directly assess and compare liability of these linkages towards enzymatic hydrolysis. We found that the additional methyl group in Thr, as compared to Ser, leads to a pronounced reduction in turn‐over by ARH3. This suggests that the additional methyl group hinders optimal substrate arrangement within the active site due to increased steric hindrance but no other hydrolase was identified as being able to hydrolyse Thr‐ADPr. In contrast, Ser‐ to Cys‐ADPr peptide **29**, in which the glycosidic bond nature differs from *O* to *S*, is almost completely resistant to ARH3‐mediated hydrolysis. Given that Cys‐modifications, which occur in human cells and are suggested to be involved in regulation of hypoxia, immunity, coronavirus response and nuclear receptors,[^6^, ^18^, ^48^, ^49^] cannot be reversed by any of the known human hydrolases, it may be that the modification is either irreversible or is reversed by an as yet unidentified hydrolase or mechanism. Our discovery of *Spy*MacroD as a Cys‐(ADP‐ribosyl)hydrolase shows that efficient and specific hydrolysis is possible, suggesting that a human enzyme harbouring this activity may exist as well.

## Conflict of interest

The authors declare no conflict of interest.

## Supporting information

As a service to our authors and readers, this journal provides supporting information supplied by the authors. Such materials are peer reviewed and may be re‐organized for online delivery, but are not copy‐edited or typeset. Technical support issues arising from supporting information (other than missing files) should be addressed to the authors.

Supporting Information
