# Supplementary material for: Molecular Tools for the Study of ADP‐Ribosylation: A Unified and Versatile Method to Synthesise Native Mono‐ADP‐Ribosylated Peptides
Source: Chemistry. 2021 May 6;27(41):10621–7. doi: 10.1002/chem.202100337 (PMC8360141; doi:10.1002/chem.202100337)
Supplement: Supplementary file 1 — Supporting Information [file CHEM-27-10621-s001.pdf]

Chemistry–A European Journal

Supporting Information

Molecular Tools for the Study of ADP-Ribosylation: A Unified and Versatile Method to Synthesise Native Mono-ADP-Ribosylated Peptides

Jim Voorneveld[+], Johannes Gregor Matthias Rack[+], Luke van Gijlswijk, Nico J. Meeuwenoord, Qiang Liu, Herman S. Overkleeft, Gijsbert A. van der Marel, Ivan Ahel,* and Dmitri V. Filippov*

Building block synthesis

Ribofuranosylated Fmoc-amino acids

The glycosylation procedure toward the respective ribofuranosylated Fmoc-amino acids was optimized by testing two ribosyl donors, the known *N*-(phenyl)trifluoroacetimidate donor **10**[1] and trichloroacetimidate[2] donor **9** (Scheme S1A). Trichloroacetamide donor **9** (Scheme S1A) was prepared in 90% yield by treating known partially protected ribose **S1**[1] with trichloroacetonitrile in the presence of DBU whereas trifluoroacetamide donor **10** was obtained in 75% yield by reacting **S1** with 2,2,2-trifluoro-*N*-phenyl-acetimidoyl chloride in the presence of Cs_2CO_3.[1]

Scheme S1B shows the synthesis of the appropriately protected serine **13**, [3,4] threonine **14**[3] and cysteine **15** to be used as the acceptors. To prepare appropriately protected Ser acceptor **13**, commercially available Fmoc-Ser-OH (**S2**) was treated with allyl bromide and DIPEA. For the synthesis of Thr and Cys acceptors **14** and **15** the corresponding side chain protected Fmoc-amino acids **S3** and **S4** were converted in allyl esters **S5** and **S6** under the same conditions as used for **13**. Subsequent removal of the acid sensitive side chain protecting groups with TFA yielded acceptors **14** and **15**.

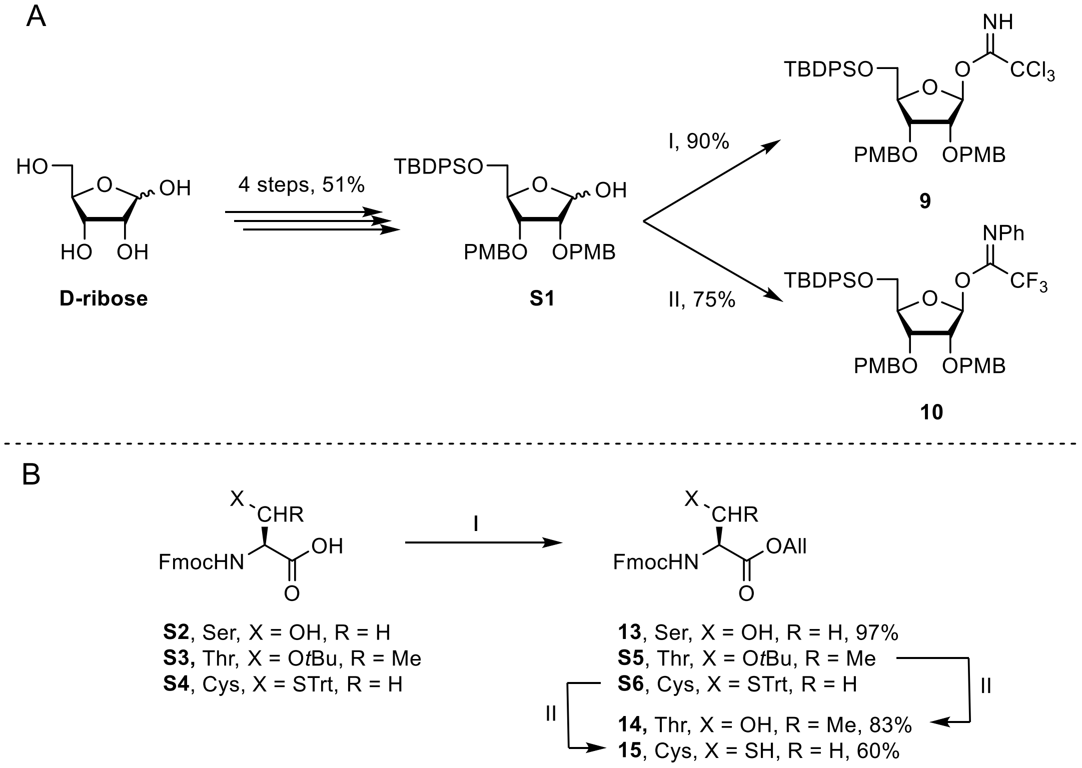

Scheme S1. A) Synthesis of ribosyl donors **9** and **10**. Reagents and conditions **I)** Cl_3CCN, DBU, acetonitrile. **II)** Cl(C=NPh)CF_3, Cs_2CO_3, acetone. **B)** synthesis of amino acid acceptors **13**, **14** and **15**. Reagents and conditions: **I)** Allyl-Br, DIPEA, DMF. **II)** TFA, DCM.

Experimental section

Expression plasmids and protein purification

The construction of the expression plasmids and the purification procedures were described earlier.[5–7] Briefly, expression plasmids were transferred into Rossetta (DE3) cells and grown to an OD_{600} of 0.6 in LB medium supplemented with appropriate antibiotics. For metal-coordinating proteins the medium was further enriched either by addition of 2 mM $MgSO_4$ (ARHs) or 100 uM $ZnCl_2$ (*Spy*MacroD). Expression was induced with 0.4 mM isopropyl β-D-1-thioglactopyranoside (IPTG) and cultured were allowed to grow further overnight at 17 °C. Cultures were harvested by centrifugation, pellets resuspended in lysis buffer (50 mM TrisHCl [pH 8], 500 mM NaCl and 25 mM imidazole) and stored at -20 °C. Proteins were purified by Ni^{2+}-NTA chromatography (Jena Bioscience) according to the manufacturer's protocol using the following buffers: all buffers contained 50 mM TrisHCl (pH 8) and 500 mM NaCl; additionally, the lysis buffer contained 25 mM, the washing buffer 40 mM, and the elution buffer 500 mM imidazole. Proteins were dialyzed overnight against 50 mM TrisHCl (pH 8), 200 mM NaCl, 1 mM dithiothreitol and 5% (v/v) glycerol and stored at -80 °C. For the purification of ARH and ARH-like proteins all purification buffers were additionally supplemented with 10 mM $MgCl_2$.

(ADP-ribosyl)hydrolase activity assay

The peptide demodification assay was described earlier[8]. Briefly, peptide concentration for the assay were estimated using absorbance at λ_{260nm} using the molar extinction coefficient of ADP-ribose (15,400 M^{-1} cm^{-1}). 20 µM indicated peptide were demodified by incubation with 1 µM hydrolase for 45 min at 30 °C in assay buffer (50 mM TrisHCl [pH 8], 200 mM NaCl, 10 mM MgCl2, 1 mM dithiothreitol and 0.2 µM human NUDT5[9]). Reactions were stopped and analyzed by performing the AMP-Glo™ assay (Promega) according to the manufacturer's protocol. Luminescence was recorded on a SpectraMax M5 plate reader (Molecular Devices) and data analyzed with GraphPad Prism 7. Control reactions were carried out in absence of peptide.

General synthetic procedures

All reagents were of commercial grade and used as received unless stated otherwise. Solvents used in synthesis were dried and stored over 4 Å molecular sieves, except MeOH and MeCN which were stored over 3 Å molecular sieves. Triethylamine (TEA) and Di-isopropylethylamine (DIPEA) were stored over KOH pellets. Column chromatography was performed on silica gel 60 Å (40-63 µm, Macherey-Nagel). TLC analysis was performed on Macherey-Nagel aluminium sheets (silica gel 60 F_{254}). TLC was used to visualize compounds by UV at wavelength 254 nm and by spraying with either cerium molybdate spray (25 g/L $(NH4)_6Mo_7O_{24}$, 10 g/L $(NH4)_4Ce(SO_4)_4 \cdot H_2O$ in 10% H_2SO_4 water solution) or $KMnO_4$ spray (20 g/L $KMnO_4$ and 10 g/L K_2CO_3 in water) followed by charring at c.a. 250 °C. LC-MS analysis was performed on a Finnigan Surveyor HPLC system with a Nucleodur C18 Gravity 3 µm 50 x 4.60 mm column (detection at 200-600 nm) coupled to a Finnigan LCQ Advantage Max mass spectrometer with ESI or coupled to a Thermo LCQ Fleet Ion mass spectrometer with ESI. The method used was 10→90% 13.5 min (0→0.5 min: 10% MeCN; 0.5→8.5 min: 10% to 90% MeCN; 8.5→11 min: 90% MeCN; 11→13.5 min: 10% MeCN) or 0→50% 13.5 min. ^{1}H and ^{13}C NMR spectra were recorded on a Bruker AV-400, AV-500 or AV-600 NMR. Chemical shifts (δ) are given in ppm relative to tetramethylsilane as internal standard. Coupling constants (*J*) are given in Hz. For

compounds **24** – **30**, a small amount of EDTA was added to the NMR sample to sharpen the peaks for ^{31}P-NMR. All given ^{13}C-APT spectra are proton decoupled.

1-(2,2,2-trichlororoacetimido)-2,3-*O*-di-(4-methoxybenzyl)-5-O-*tert*-butyldiphenylsilyl -α,β-D-ribofuranoside (9)

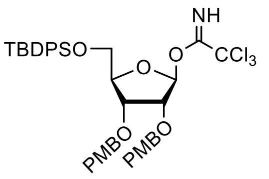

2,3-bis-*O*-(4-methoxybenzyl)-5-*O*-((*tert*-butyl)-diphenylsilyl)-α,β-D-ribofuranoside **S1** (1.01 gram, 1.6 mmol, 1 eq.) was co-evaporated thrice with toluene, dissolved in dry DCM (16 mL, 0.1 M) and the solution was cooled to 0 °C. DBU (0.11 mL, 0.74 mmol, 0.5 eq.) and trichloroacetonitrile (0.8 mL, 8.0 mmol, 5 eq.) were added and the solution was stirred at 0 °C for 1 hour after which the reaction was concentrated *in vacuo*. Purification by flash column chromatography in neutralized silica (5 -> 20% EtOAc in pentane with 1% TEA) yielded titled compound as a pale oil (1.11 g, 1.44 mmol, 90%). **Rf**: 0.85 (20% EtOAc in Pentane). **^{1}H NMR** (400 MHz, $CDCl_3$) δ 8.48 (s, 1H, NH acetimidate), 7.69 – 7.54 (m, 6H, H arom.), 7.46 – 7.33 (m, 6H, H arom.), 7.36 – 7.25 (m, 7H, H arom.), 7.18 (s, 1H, H arom.), 6.84 (dd, *J* = 25.9, 8.7 Hz, 3H, H arom.), 6.31 (s, 1H, H-1), 4.80 – 4.64 (m, 1H, CH_2 PMB), 4.60 (d, *J* = 11.7 Hz, 1H, CH_2 PMB), 4.46 (d, *J* = 11.3 Hz, 1H, CH_2 PMB), 4.42 – 4.34 (m, 2H, H-4 + CH_2 PMB), 4.17 – 4.11 (m, 1H, H-3), 4.06 (d, *J* = 4.8 Hz, 1H, H-2), 3.86 – 3.79 (m, 4H, CH_3 PMB + H-5), 3.79 – 3.76 (m, 4H, CH_3 PMB + H-5), 1.02 (s, 9H TBDPS). **^{13}C NMR** (101 MHz, $CDCl_3$) δ 161.1, 159.4, 135.7, 133.4, 130.0, 129.6, 127.8, 113.9, 103.9 (C-1), 83.7 (C-4), 78.6 (C-2), 76.6 (C-3), 72.1, 71.8 (CH_2 PMB), 64.2 (H-5), 55.4, (CH_3 PMB), 27.0 (CH_3 TBDPS), 19.4 (Cq TBDPS).

1-*O*-((*N*-phenyl)-2,2,2-trifluoroacetimido) 2,3-bis-*O*-(4-methoxybenzyl)-5-*O*-((*tert*-butyl)-diphenylsilyl)-α,β-D-ribofuranoside (10)

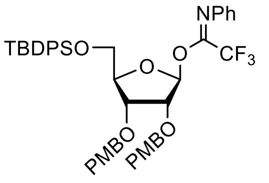

2,3-bis-*O*-(4-methoxybenzyl)-5-*O*-((*tert*-butyl)-diphenylsilyl)-α,β-D-ribofuranoside **S1** (6.28 gram, 10.0 mmol) was dissolved in acetone (50 mL, 0.2M). Cs_2CO_3 (4.89 gram, 15.0 mmol, 1.5 eq.) and 2,2,2-trifluoro-*N*-phenylacetimidoyl chloride (1.74 mL, 11.0 mmol, 1.1 eq.) were added. The suspension was stirred for 2 hours before TLC indicated full conversion into a higher running product (). The reaction was filtered over a pad of Celite and the filtrate was concentrated *in vacuo*. Flash column chromatography in neutralized silica (10 -> 20% Et_2O in pentane) yielded titled compound as a pale oil (6.01 g, 7.51 mmol, 75%). Spectral data was in accordance with literary precedence.[1] **Rf:** 0.20 (10% Et_2O in pentane) **^{1}H NMR** (400 MHz, $CDCl_3$) δ 7.74 – 7.66 (m, 4H, TBDPS arom. α+β), 7.66 – 7.50 (m, 4H, NPh arom. α+β), 7.46 – 7.32 (m, 12H, TBDPS arom. + NPh arom. α+β), 7.32 – 7.17 (m, 12H, PMB arom. α + β), 7.12 – 7.00 (m, 2H, PMB arom. α), 6.91 – 6.72 (m, 12H, PMB arom. α + β), 6.48 (bs, 1H, H-1α), 6.30 (bs, 1H, H-2β), 4.82 – 4.57 (m, 4H, 2x CH_2 PMB α/β), 4.57 – 4.40 (m, 4H, 2x CH_2 PMB α/β), 4.40 – 4.31 (m, 2H, H-4 α+β), 4.26 (t, *J* = 5.5 Hz, 1H, H-3β), 4.19 – 4.01 (m, 3H, H-2α + H-2β + H-3α), 4.00 – 3.86 (m, 1H, H-5$_a$β), 3.84 – 3.56 (m, 15H, H-5$_b$β + H-5α + 2x CH_3 PMB α+β), 1.07 (s, 9H, *t*Bu TBDPS β), 0.97 (s, 9H, *t*Bu TBDPS α).**^{13}C NMR** (101 MHz, $CDCl_3$) δ 159.5, 159.4, 159.4, 159.2 (Cq PMB), 144.3, 143.9 (Cq NPh), 135.7, 135.6, 135.6, 135.5(CH arom. TBDPS/NPh), 133.3, 133.1, 133.0, 132.7, 130.4 (Cq TBDPS/PMB), 129.9, 129.9, 129.7, 129.7 (CH arom.), 129.6 (Cq), 129.5 (CH arom.), 129.4 (Cq), 129.4, 129.3, 129.2, 128.7, 128.7, 127.8, 127.8, 127.7 (CH arom.), 124.2, 120.6, 119.6, 114.3, 113.9, 113.8, 113.8, 113.7 (CH arom. PMB), 102.8 (C-1β), 85.7 (C-4α), 83.4 (C-4β), 78.7 (C-2α), 78.4 (C-2β), 76.0 (C-3β), 75.2 (C-3α),

72.9, 72.3, 72.2, 71.9 (CH_2PMB), 63.7 (C-5β), 63.3 (C-5α), 55.5, 55.2, 55.1, 55.1 (CH_3 PMB), 26.8, 26.7 (CH_3 *t*Bu), 19.2, 19.2 (Cq *t*Bu).

N-fluorenylmethoxycarbonyl-serine-O-allyl ester (13)

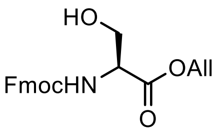

Fmoc-Ser-OH (3.45 g, 10 mmol, 1 eq) was co-evaporated thrice with 1,4-dioxane and dissolved DMF (50 mL, 0.2M). DIPEA (2.10 mL, 12 mmol, 1.2 eq) was added followed by the subsequent addition of allyl-bromide (1.04 mL, 12 mmol, 1.2 eq) and stirred overnight. The reaction was quenched with 100 mL water and transferred into a separatory funnel. The reaction mixture was extracted three times with Et2O. The combined organics were washed three times with brine, dried over MgSO4 and concentrated *in vacuo*. Flash column chromatography (10 -> 40% EtOAc in pentane) afforded titled compound as a white solid (3.58 g, 9.74 mmol, 97%). **Rf:** 0.49 (40% EtOAc in pentane). **^{1}H NMR** (400 MHz, $CDCl_3$) δ 7.77 (d, *J* = 7.5 Hz, 2H, Fmoc arom.), 7.61 (d, *J* = 6.8 Hz, 2H, Fmoc arom.), 7.41 (t, *J* = 7.4 Hz, 2H, Fmoc arom.), 7.32 (t, *J* = 7.5 Hz, 2H, Fmoc arom.), 5.99 – 5.84 (m, 1H, OCH_2CHCH_2), 5.73 (d, *J* = 7.2 Hz, 1H, NH), 5.35 (d, *J* = 17.1 Hz, 1H, CH2 OCH_2CHCH_{2a}), 5.27 (d, *J* = 10.4 Hz, 1H, OCH_2CHCH_{2b}), 4.69 (d, *J* = 5.5 Hz, 2H, OCH_2CHCH_2), 4.51 – 4.40 (m, 3H, CH Ser + CH_2 Ser), 4.23 (t, *J* = 6.9 Hz, 1H, CH Fmoc), 4.04 (d, *J* = 10.7 Hz, 1H, CH_2 Fmoc), 3.95 (d, *J* = 10.6 Hz, 1H, CH_2 Fmoc), 2.15 (s, 1H, OH Ser). **^{13}C NMR** (101 MHz, CDCl3) δ 170.4 (C=O COOAll), 156.4 (C=O Fmoc), 143.9, 143.8, 141.4 (Cq Fmoc), 131.4 (OCH_2CHCH_2), 127.9, 127.2, 125.2, 120.1 (CH Fmoc arom.), 119.1 (OCH2CH*CH2*), 67.3 (CH_2 Fmoc), 66.5 (OCH_2CHCH_2), 63.3 (CH_2 Ser), 56.2 (CH Ser), 47.2 (CH Fmoc).

N-fluorenylmethoxycarbonyl-threonine-O-allyl ester (14)

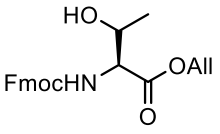

Fmoc-Cys(Trt)-OH (1.98 gram, 5 mmol) was co-evaporated thrice with 1,4-dioxane and dissolved in DMF (25 mL, 0.2M). DIPEA (1.04 mL, 6.0 mmol, 1.2 eq.) was added followed by allyl bromide (0.52 mL, 6.0 mmol, 1.2 eq.). The resulting solution was stirred overnight. The reaction was carefully quenched by the addition of water and the resulting slurry was transferred into a separatory funnel. The water layer was extracted thrice with Et_2O and the combined organics were washed with brine, dried over $MgSO_4$ and concentrated *in vacuo*. The crude allylated cysteine was dissolved in 25 mL of a 10% TFA solution in DCM and TIS (4.1 mL, 20 mmol, 4.0 eq.) was added. The resulting solution was stirred for 3 hours after which TLC showed full conversion of the starting material. The reaction was diluted with toluene and concentrated *in vacuo*. The resulting oil was co-evaporated thrice with toluene. Flash column chromatography (10 -> 40% EtOAc in pentane) yielded titled compound as a white solid (1.58 gram, 4.15 mmol, 83%). **Rf:** 0.65 (40% EtOAc in pentane). **^{1}H NMR** (400 MHz, $CDCl_3$) δ 7.77 (dt, *J* = 7.6, 0.9 Hz, 2H, Fmoc arom.), 7.62 (dd, *J* = 7.7, 4.2 Hz, 2H, Fmoc arom.), 7.46 – 7.38 (m, 2H, Fmoc arom.), 7.33 (dt, *J* = 7.5, 1.5 Hz, 2H, Fmoc arom.), 5.92 (ddt, *J* = 16.4, 11.0, 5.8 Hz, 1H, OCH_2CHCH_2), 5.60 (d, *J* = 9.1 Hz, 1H, NH), 5.35 (dd, *J* = 16.2, 1.7 Hz, 1H, OCH_2CHCH_{2a}), 5.27 (dd, *J* = 1.1 Hz, 1H, OCH_2CHCH_{2b}), 4.69 (d, *J* = 5.7 Hz, 2H, OCH_2CHCH_2), 4.49 – 4.34 (m, 4H, CH_2 Fmoc + 2x CH Thr), 4.25 (t, *J* = 7.1 Hz, 1H, CH Fmoc), 1.77 (s, 1H, OH), 1.27 (d, *J* = 6.3 Hz, 3H, CH_3 Thr). **^{13}C NMR** (101 MHz, $CDCl_3$) δ 144.0, 141. 5, 131.5 (OCH_2CHCH_2), 127.9, 127.2, 125.3, 120.1, 119.2 ($OCH2CHCH_2$), 68.2 (CH Thr), 67.4 (CH_2 Fmoc), 66.4 (OCH_2CHCH2), 59.2 (CH Thr), 47.3 (CH Fmoc), 20.1 (CH_3 Thr).

_N_-fluorenylmethoxycarbonyl-cysteine-_O_-allyl ester (15)

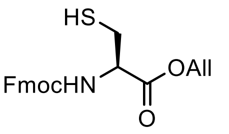

Fmoc-Cys(Trt)-OH (2.23 gram, 3.81 mmol) was co-evaporated thrice with toluene and dissolved in DMF (19 mL, 0.2M). DIPEA (0.80 mL, 4.6 mmol, 1.2 eq.) was added followed by allyl bromide (0.40 mL, 4.6 mmol, 1.2 eq.). The resulting solution was stirred overnight. The reaction was carefully quenched by the addition of water and the resulting slurry was transferred into a separatory funnel. The water layer was extracted thrice with Et_2O and the combined organics were washed with brine, dried over $MgSO_4$ and concentrated *in vacuo*. The crude allylated cysteine was dissolved in 19 mL of a 10% TFA solution in DCM and TIS (3.1 mL, 15.2 mmol, 4.0 eq.) was added. The resulting solution was stirred for 4 hours in a dark environment after which TLC showed full conversion of the starting material. The reaction was diluted with toluene and concentrated *in vacuo*. The resulting oil was co-evaporated thrice with toluene. Flash column chromatography (5 -> 10% EtOAc in toluene) yielded titled compound as a white solid (875 mgram, 2.28 mmol, 60%). **Rf:** 0.53 (10% EtOAc in toluene). **^{1}H NMR** (400 MHz, CDCl3) δ 7.76 (dd, J = 7.6, 1.1 Hz, 2H, Fmoc arom.), 7.60 (d, J = 7.5 Hz, 2H, Fmoc arom.), 7.40 (t, J = 8.3, 6.8 Hz, 2H, Fmoc arom.), 7.32 (tt, J = 7.4, 1.5 Hz, 2H, Fmoc arom.), 5.92 (ddt, J = 16.5, 10.4, 5.8 Hz, 1H, $OCH_2C\mathit{H}CH_2$), 5.72 (bs, 1H, NH), 5.43 – 5.18 (m, 2H, $OCH_2CHC\mathit{H_2}$), 4.76 – 4.61 (m, 3H, CH Cys + $OC\mathit{H_2}CHCH_2$), 4.51 – 4.32 (m, 2H, CH_2 Fmoc), 4.23 (t, J = 6.9 Hz, 1H, CH Fmoc), 3.01 (dqt, J = 14.0, 8.8, 4.2 Hz, 2H, CH_2 Cys), 1.37 (t, J = 9.0 Hz, 1H, SH). **^{13}C NMR** (101 MHz, CDCl3) δ 169.8 (C=O COOAll), 155.8 (C=O Fmoc), 143.9, 143.7, 141.4, 141.4 (Cq Fmoc), 131.4 ($OCH_2\mathit{C}HCH_2$), 127.9, 127.2, 127.2, 125.2, 125.2, 120.2, 120.1 (CH Fmoc arom.), 119.5 ($OCH_2CH\mathit{C}H_2$), 67.2 (CH_2 Fmoc), 66.6 ($O\mathit{C}H_2CHCH_2$), 55.3 (CH Cys), 47.3 (CH Fmoc), 27.3 (CH_2 Cys).

1-_O_-(2,3-bis-_O_-(4-methoxybenzyl)-5-_O_-((_tert_-butyl)-diphenylsilyl)-α-D-ribosyl)-_N_-fluorenylmethoxycarbonyl serine allyl ester (12)

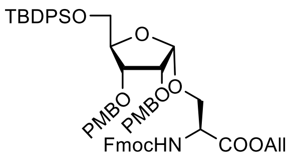

1-*O*-((*N*-phenyl)-2,2,2-trifluoroacetimido) 2,3-bis-*O*-(4-methoxybenzyl)-5-*O*-((*tert*-butyl)-diphenylsilyl)-α,β-D-ribofuranoside **10** (1.60 gram, 2.00 mmol, 1.1 eq.) and Fmoc-Ser-OAll **13** (669 mgram, 1.82 mmol, 1.0 eq. relative to the donor) were co-eavporated thrice with toluene and dissolved in DCM (20 mL, 0.1M relative to the donor). The reaction was cooled to -50 °C and TBSOTf (46 µL, 0.2 mmol, 0.1 eq. relative to the donor) was added. The reaction was stirred at -50 °C for 4.5 hours before TLC analysis showed near full conversion of the starting material into a higher running product. The reaction was quenched with TEA and evaporated *in vacuo*. Flash column chromatography (0.5 -> 3% acetone in DCM) yielded titled compound as a clear oil (994 mgram, 1.02 mmol, 56%). **Rf:** 0.60 (3% acetone in DCM). **^{1}H NMR** (400 MHz, $CDCl_3$) δ 7.72 – 7.65 (m, 2H, Fmoc arom.), 7.65 – 7.49 (m, 6H, Fmoc arom. + TBDPS arom.), 7.46 – 7.10 (m, 14H, Fmoc arom. + TBDPS arom. + PMB arom. + $CHCl_3$ overlap), 6.89 – 6.74 (m, 4H, PMB arom.), 6.61 (d, *J* = 9.0 Hz, 1H, NH), 5.88 (ddt, *J* = 16.1, 10.8, 5.5 Hz, 1H, $CHC\mathit{H}CH_2$), 5.31 (dd, *J* = 17.2, 1.6 Hz, 1H, $CH_2CHC\mathit{H}_{2a}$), 5.16 (dd, *J* = 10.5, 1.5 Hz, 1H, $CH_2CHC\mathit{H}_{2b}$), 4.98 (d, *J* = 4.1 Hz, 1H, H-1), 4.73 – 4.46 (m, 7H, CH Ser + $C\mathit{H_2}CHCH_2$ + 2x CH_2 PMB), 4.39 (dd, *J* = 10.5, 6.9 Hz, 1H, CH_{2a} Fmoc), 4.28 – 4.15 (m, 3H, H-4 + CH_{2b} Fmoc + CH_{2a} Ser), 4.12 (t, *J* = 7.3 Hz, 1H, CH Fmoc), 4.03 (dd, *J* = 6.1, 2.3 Hz, 1H, H-3), 3.97 (dd, *J* = 10.3, 3.3 Hz, 1H, CH_{2b} Ser), 3.89 (dd, *J* = 6.1, 4.1 Hz, 1H, H-2), 3.74 (s, 3H, CH_3 PMB), 3.69 (s, 3H, CH_3 PMB), 3.61 (ddd, *J* = 38.2, 11.3, 3.2 Hz, 2H, H-5), 0.97 (s, 9H, *t*Bu TBDPS). **^{13}C NMR** (101 MHz, $CDCl_3$) δ 170.3 (C=O COOAll), 159.4, 159.1 (Cq PMB), 156.5 (C=O Fmoc), 144.1,

143.8, 141.2, 141.1 (Cq Fmoc), 135.6, 135.5 (CH arom. TBDPS), 133.1, 132.9 (Cq TBDPS), 131.8 (CH_2*C*HCH_2), 130.3 (Cq PMB), 129.9 (CH arom.), 129.8 (Cq PMB), 129.8, 129.7, 129.5, 127.8, 127.8, 127.6, 127.5, 127.0, 127.0, 125.4, 125.1 (CH arom.), 119.9, 119.8 (CH arom. Fmoc), 118.3 (CH_2CH*C*H_2), 113.8, 113.7 (CH arom. PMB), 101.0 (C-1), 84.1 (C-4), 78.6 (C-2), 75.2 (C-3), 72.3, 72.1 (CH_2 PMB), 67.3 (CH_2 Ser), 67.0 (CH_2 Fmoc), 66.0 (*C*H_2CHCH_2), 64.0 (C-5), 55.2, 55.1 (CH_3PMB), 54.5 (CH Ser), 47.1 (CH Fmoc), 26.8 (CH_3 *t*Bu), 19.2 (Cq *t*Bu). **HRMS:** $[C_{58}H_{63}NO_{11}Si + Na]^+$ found: 1000.4053, calculated: 1000.4063

1-*O*-(2,3-bis-*O*-(4-methoxybenzyl)-5-*O*-((*tert*-butyl)-diphenylsilyl)-α-D-ribosyl)-*N*-fluorenylmethoxycarbonyl threonine allyl ester (16)

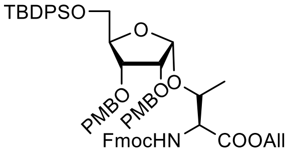

1-*O*-((*N*-phenyl)-2,2,2-trifluoroacetimido) 2,3-bis-*O*-(4-methoxybenzyl)-5-*O*-((*tert*-butyl)-diphenylsilyl)-α,β-D-ribofuranoside **10** (1.22 gram, 1.53 mmol, 1.3 eq.) and Fmoc-Thr-OAll **14** (450 mgram, 1.18 mmol, 1.0 eq. relative to the donor) were co-eavporated thrice with toluene and dissolved in DCM (15 mL, 0.1M relative to the donor). The reaction was cooled to -50 °C and TBSOTf (35 µL, 0.15 mmol, 0.1 eq. relative to the donor) was added. The reaction was stirred at -50 °C for 2 hours before TLC analysis showed near full conversion of the starting material into a higher running product. The reaction was quenched with TEA and evaporated *in vacuo*. Flash column chromatography (0.5 -> 3% acetone in DCM) yielded titled compound as a clear oil (742 mgram, 0.748 mmol, 63%). **Rf:** 0.57 (3% acetone in DCM). **^{1}H NMR** (400 MHz, $CDCl_3$) δ 7.70 (d, *J* = 7.5 Hz, 2H, Fmoc arom.), 7.67 – 7.52 (m, 6H, Fmoc arom. + TBDPS arom.), 7.46 – 7.28 (m, 10H, Fmoc arom. + TBDPS arom.), 7.28 – 7.15 (m, 4H, PMB arom.), 6.91 – 6.75 (m, 4H, PMB arom.), 6.62 (d, *J* = 8.7 Hz, 1H, NH), 5.88 (ddt, *J* = 17.3, 10.5, 5.7 Hz, 1H, OCH_2C*H*CH_2), 5.37 – 5.26 (m, 1H, OCH_2CHCH_{2a}), 5.21 – 5.15 (m, 1H, OCH_2CHCH_{2b}), 5.13 (d, *J* = 4.2 Hz, 1H, H-1), 4.71 – 4.55 (m, 4H, OCH_2CHCH_2 + CH_2 PMB) , 4.55 – 4.36 (m, 5H CH_2 PMB + CH_{2a} Fmoc + 2x CH Thr), 4.30 (dd, *J* = 10.3, 7.6 Hz, 1H, CH_{2b} Fmoc), 4.23 (t, *J* = 7.3 Hz, 1H, CH Fmoc), 4.18 (q, *J* = 3.3 Hz, 1H, H-4), 3.95 (dd, *J* = 6.6, 3.0 Hz, 1H, H-3), 3.82 (dd, *J* = 6.6, 4.2 Hz, 1H, H-2), 3.76 – 3.61 (m, 7H, 2x CH_3 PMB + H-5_a), 3.55 (dd, *J* = 11.3, 3.4 Hz, 1H, H-5_b), 1.40 (d, *J* = 6.3 Hz, 3H, CH_3 Thr), 0.97 (s, 9H, *t*Bu TBDPS).**^{13}C NMR** (101 MHz, $CDCl_3$) δ 170.7 (C=O, COOAll), 159.3, 159.1 (Cq PMB), 157.0 (C=O Fmoc), 144.0, 143.8, 141.2, 141.1 (Cq Fmoc), 135.5, 135.5 (CH arom. TBDPS), 133.1, 132.9 (Cq TBDPS), 131.6 (OCH_2*C*HCH_2), 130.1 (Cq PMB), 129.8, 129.7 (CH arom.), 129.6 (Cq PMB), 129.5, 129.4, 127.7, 127.7, 127.6, 127.5, 127.0, 127.0, 125.3, 125.2 (CH arom.), 119.8 (CH arom. Fmoc), 118.7 (OCH_2CH*C*H_2), 113.8, 113.7 (CH arom. PMB), 101.4 (C-1), 83.6 (C-4), 77.8 (C-2), 74.8 (C-3), 74.4 (RO-*C*H(CH_3)CH-R Thr), 72.0, 71.8 (CH_2 PMB), 67.1 (CH_2 Fmoc), 65.9 (O*C*H_2CHCH_2), 63.9 (C-5), 59.4 (RO-CH(CH_3)*C*H-R Thr), 55.1, 55.0 (CH_3 PMB), 47.1 (CH Fmoc), 26.7 (CH_3 *t*Bu), 19.1 (Cq *t*Bu), 19.1 (CH_3 Thr). **HRMS:** $[C_{59}H_{65}NO_{11}Si + H]^+$ found: 992.4402, calculated: 992.4400.

1-*O*-(2,3-bis-*O*-(4-methoxybenzyl)-5-*O*-((*tert*-butyl)-diphenylsilyl)-α-D-ribosyl)-*N*-fluorenylmethoxycarbonyl cysteine allyl ester (17)

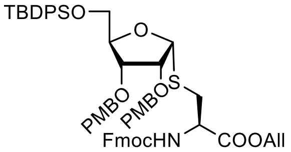

1-*O*-((*N*-phenyl)-2,2,2-trifluoroacetimido) 2,3-bis-*O*-(4-methoxybenzyl)-5-*O*-((*tert*-butyl)-diphenylsilyl)-α,β-D-ribofuranoside **10** (1.60 gram, 2.00 mmol, 1.3 eq.) and Fmoc-Cys-OAll **15** (595 mgram, 1.55 mmol, 1.0 eq. relative to the donor) were co-eavporated thrice with toluene and dissolved in DCM (20 mL, 0.1M relative to the donor). The reaction was cooled to -50 °C and TBSOTf (46 µL, 0.2 mmol, 0.1 eq. relative to the

donor) was added. The reaction was stirred at -50 °C for 1.5 hours before TLC analysis showed near full conversion of the starting material into a higher running product. The reaction was quenched with TEA and evaporated *in vacuo*. Flash column chromatography (0.5 -> 1.5% acetone in DCM) yielded titled compound as a clear oil (1.08 gram, 1.08 mmol, 70%). **Rf:** 0.65 (1.5% acetone in DCM). **^{1}H NMR** (400 MHz, $CDCl_3$) δ 7.73 – 7.62 (m, 2H, Fmoc arom.), 7.62 – 7.43 (m, 6H, Fmoc arom. + TBDPS arom.), 7.42 – 7.06 (m, 14H, Fmoc arom. + TBDPS arom. + PMB arom.), 6.90 – 6.77 (m, 4H, PMB arom.), 6.67 (d, *J* = 8.8 Hz, 1H, NH), 5.87 (ddt, *J* = 16.3, 10.8, 5.7 Hz, 1H, OCH2C*H*CH2), 5.39 (d, *J* = 5.5 Hz, 1H, H-1), 5.28 (dq, *J* = 17.2, 1.5 Hz, 1H, OCH_2CHCH_{2a}), 5.15 (dd, *J* = 10.4, 1.4 Hz, 1H, OCH_2CHCH_{2b}), 4.77 (dt, *J* = 8.9, 4.4 Hz, 1H, CH Cys), 4.72 – 4.53 (m, 5H, OCH_2CHCH_2 + 1x CH_2 PMB + 1x CH_{2a} PMB), 4.47 (d, *J* = 11.8 Hz, 1H, CH_{2b} PMB), 4.42 – 4.26 (m, 2H, H-4 + CH_{2a} Fmoc), 4.21 – 3.97 (m, 4H, H-2 + H-3 + CH_{2b} Fmoc + CH fmoc), 3.77 (dd, *J* = 11.4, 3.3 Hz, 1H, H-5_a), 3.74 – 3.70 (m, 6H, 2x CH_3 PMB), 3.66 (dd, *J* = 11.3, 2.9 Hz, 1H, H-5_b), 3.44 (dd, *J* = 14.6, 5.0 Hz, 1H, CH_{2a} Cys), 2.95 (dd, *J* = 14.6, 3.9 Hz, 1H, CH_{2b} Cys), 0.96 (s, 9H, *t*Bu TBDPS). **^{13}C NMR** (101 MHz, $CDCl_3$) δ 170.2 (C=O COOAll, 159.3, 159.1 (Cq PMB), 156.1 (C=O Fmoc), 143.9, 143.7, 141.1, 141.0 (Cq Fmoc), 135.4, 135.4 (CH arom. TBDPS), 132.9, 132.8 (Cq TBDPS), 131.6 (OCH_2CHCH_2), 130.0 (Cq PMB), 129.7, 129.6, 129.5 (CH arom.), 129.4 (Cq PMB), 129.2, 127.7, 127.6, 127.6, 127.5, 127.5, 126.9, 126.9, 125.2, 125.0 (CH arom.), 119.7 (CH arom. Fmoc) 118.5 (OCH_2CHCH_2), 113.7, 113.6 (CH arom. PMB), 89.1 (C-1), 82.6 (C-4), 78.0 (C-2), 76.0 (C-3), 72.5, 72.0 (CH_2 PMB), 66.9 (CH_2 Fmoc), 65.9 (OCH_2CHCH_2), 63.3 (C-5), 55.1 (CH_3 PMB), 54.2 (CH Cys), 46.9 (CH Fmoc), 34.8 (CH_2 Cys), 26.7 (CH_3 *t*Bu), 19.1 (Cq *t*Bu). **HRMS:** $[C_{58}H_{63}NO_{10}SSi + H]^+$ found: 994.4017, calculated: 994.4015.

1-*O*-(2,3-bis-*O*-(4-methoxybenzyl)-5-*O*-((*tert*-butyl)-diphenylsilyl)-α-D-ribosyl)-*N*-fluorenylmethoxycarbonyl serine (1)

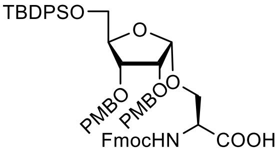

1-*O*-(2,3-bis-*O*-(4-methoxybenzyl)-5-*O*-((tert-butyl)-diphenylsilyl)-α-D-ribosyl)-*N*-fluorenylmethoxycarbonyl serine allyl ester **12** (836 mgram, 0.855 mmol, 1.0 eq.) was dissolved in DCM (8.5 mL, 0.1M). DMBA (265 mgram, 1.70 mmol, 2.0 eq.) and $Pd(PPh_3)_4$ (10 mgram, 8.5 µmol, 0.01 eq.) were added and the reaction was stirred for 1 hour before TLC showed full conversion of the starting material into a lower running product. The reaction was diluted with DCM, washed with 1M HCl and the organics were dried over $MgSO_4$, filtered and concentrated *in vacuo*. Flash column chromatography in (4% MeOH in DCM + 0.1% AcOH) yielded titled compound as a white foam (724 mgram, 0.772 mmol, 90%). **Rf:** 0.38 (5% MeOH in DCM + 0.1% AcOH). $[\alpha]_D^{20}$ = +50.8° (c = 1.0, $CHCl_3$). **^{1}H NMR** (400 MHz, $CDCl_3$) δ 7.70 (d, *J* = 7.6 Hz, 2H, Fmoc arom.), 7.61 – 7.49 (m, 6H, Fmoc arom. + TBDPS arom.), 7.49 – 7.13 (m, 14H, Fmoc arom. + TBDPS arom. + PMB arom.), 6.86 – 6.72 (m, 4H, PMB arom.), 6.27 – 6.18 (m, 1H, NH), 4.93 (d, *J* = 4.1 Hz, 1H, H-1), 4.62 – 4.44 (m, 5H, CH Ser + 2x CH_2 PMB), 4.42 – 4.26 (m, 2H, CH_2 Fmoc), 4.20 – 4.09 (m, 2H, H-4 + CH Fmoc), 4.06 – 3.99 (m, 1H, CH_{2a} Ser), 3.97 (dd, *J* = 6.1, 1.6 Hz, 1H, H-3), 3.94 – 3.85 (m, 2H, CH_{2b} Ser + H-2), 3.79 – 3.69 (m, 6H, 2x CH_3 PMB), 3.56 (dd, *J* = 11.3, 3.5 Hz, 1H, H-5_a), 3.44 (dd, *J* = 11.3, 2.8 Hz, 1H, H-5_b), 0.95 (s, 9H, *t*Bu TBDPS). **^{13}C NMR** (101 MHz, $CDCl_3$) δ 172.0 (C=O COOH), 159.6, 159.4 (Cq PMB), 156.4 (C=O Fmoc), 141.4, 141.3 (Cq Fmoc), 135.7, 135.6 (CH arom. TBDPS), 133.1, 132.8 (Cq TBDPS), 130.1, 130.0, 130.0, 129.9 (CH arom.), 129.7, 129.2 (Cq PMB), 127.9, 127.9, 127.8, 127.2, 125.3, 125.2 (CH arom.), 120.0 (CH arom. Fmoc), 114.1, 113.9 (CH arom. PMB), 101.5 (C-1), 84.6 (C-4), 78.2 (C-2), 74.9 (C-3), 72.3 (CH_2 PMB), 67.2 (CH_2 Fmoc), 66.7 (CH_2

Ser), 64.1 (C-5), 55.3 (CH_3 PMB), 53.6 (CH Ser), 47.2 (CH Fmoc), 26.9 (CH_3 *t*Bu), 19.3 (Cq *t*Bu). **HRMS:** $[C_{55}H_{59}NO_{11}Si + Na]^+$ found: 960.3756, calculated: 960.3750.

1-*O*-(2,3-bis-*O*-(4-methoxybenzyl)-5-*O*-((*tert*-butyl)-diphenylsilyl)-α-D-ribosyl)-*N*-fluorenylmethoxycarbonyl threonine (2)

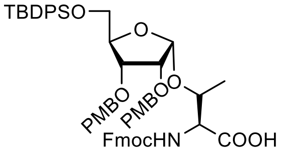

1-*O*-(2,3-bis-*O*-(4-methoxybenzyl)-5-*O*-((tert-butyl)-diphenylsilyl)-α-D-ribosyl)-*N*-fluorenylmethoxycarbonyl threonine allyl ester **16** (525 mgram, 0.529 mmol, 1.0 eq.) was dissolved in DCM (5.3 mL, 0.1M). DMBA (372 mgram, 2.38 mmol, 4.5 eq.) and a catalytic amount of $Pd(PPh_3)_4$ were added and the reaction was stirred for 15 minutes before TLC showed full conversion of the starting material into a lower running product. The reaction was diluted with DCM, washed with sat. aq. $NaHCO_3$. The water layer was extracted six times with DCM. The combined organics were dried over $MgSO_4$, filtered and concentrated *in vacuo*. After purification by flash column chromatography in (1 -> 10% MeOH in DCM) all fractions containing titled compound were combined and concentrated *in vacuo.* The residue was taken up in DCM and washed with 10% aqueous citric acid. The water layer was extracted twice with DCM and the combined organics were dried over $MgSO_4$, filtered and evaporated *in vacuo* yielding titled compound as an off-white foam (495 mgram, 0.520 mmol, 98%). **Rf:** 0.05 (25% EtOAC in pentane + 0.1% AcOH). $[\alpha]_D^{20}$ = +43.2° (c = 1.0, $CHCl_3$). **^{1}H NMR** (400 MHz, $CDCl_3$) δ 7.74 (d, *J* = 7.2 Hz, 2H, Fmoc arom.), 7.64 – 7.55 (m, 6H, Fmoc arom. + TBDPS arom.), 7.49 – 7.32 (m, 10H, Fmoc arom. + TBDPS arom.), 7.28 (tt, *J* = 7.5, 1.4 Hz, 4H, Fmoc arom. + TBDPS arom.), 7.22 (t, *J* = 8.3 Hz, 4H, PMB arom.), 6.86 – 6.74 (m, 4H, PMB arom.), 6.13 (d, *J* = 6.7 Hz, 1H, NH), 5.20 (d, *J* = 4.1 Hz, 1H, H-1), 4.58 – 4.47(m, 5H, 2x CH_2 PMB + CH Thr), 4.42 – 4.33 (m, 2H, CH_2 Fmoc), 4.26 (dd, *J* = 6.6, 4.4 Hz, 1H, CH Thr), 4.24 – 4.16 (m, 2H, CH Fmoc + H-4), 3.95 (dd, *J* = 6.4, 1.9 Hz, 1H, H-3), 3.89 (dd, *J* = 6.3, 4.2 Hz, 1H, H-2), 3.75 (s, 3H, CH_3 PMB), 3.71 (s, 3H, CH_3 PMB), 3.64 – 3.57 (m, 1H, H-5_a), 3.51 (dd, *J* = 11.3, 3.0 Hz, 1H, H-5_b), 1.27 (d, *J* = 6.5 Hz, 3H, CH_3 Thr), 0.97 (s, 9H, *t*Bu TBDPS). **^{13}C NMR** (101 MHz, $CDCl_3$) δ 171.4 (C=O COOH), 159.6, 159.3 (Cq PMB), 156.1 (C=O Fmoc), 143.9, 143.7, 141.3, 41.3 (Cq Fmoc), 135.6, 135.6 (CH arom. TBDPS), 133.1, 132.8 (Cq TBDPS), 129.9, 129.9, 129.8, 129.6 (CH arom.), 128.9 (Cq PMB), 127.8, 127.8, 127.8, 127.1, 125.2 (CH arom.), 120.0, 120.0 (CH arom. Fmoc), 114.0, 113.8 (CH arom. PMB), 103.1 (C-1), 84.7 (C-4), 78.0 (C-2), 74.9 (CH Thr), 74.5 (C-3), 72.2, 72.1 (CH_2 PMB), 67.2 (CH_2 Fmoc), 64.1 (C-5), 57.8 (CH Thr), 55.3, 55.2 (CH_3 PMB), 47.2 (CH Fmoc), 26.8 (CH_3 *t*Bu), 19.2 (Cq *t*Bu), 16.9 (CH_3 Thr). **HRMS:** $[C_{56}H_{61}NO_{11}Si + Na]^+$ found: 974.3910, calculated: 974.3906.

1-*O*-(2,3-bis-*O*-(4-methoxybenzyl)-5-*O*-((*tert*-butyl)-diphenylsilyl)-α-D-ribosyl)-*N*-fluorenylmethoxycarbonyl cysteine (3)

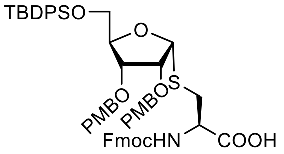

1-*O*-(2,3-bis-*O*-(4-methoxybenzyl)-5-*O*-((tert-butyl)-diphenylsilyl)-α-D-ribosyl)-*N*-fluorenylmethoxycarbonyl cysteine allyl ester **17** (1.08 gram, 1.09 mmol, 1.0 eq.) was dissolved in DCM (11 mL, 0.1M). DMBA (775 mgram, 4.95 mmol, 4.5 eq.) and a catalytic amount of $Pd(PPh_3)_4$ were added and the reaction was stirred for 15 minutes before TLC showed full conversion of the starting material into a lower running product. The reaction was diluted with DCM, washed with sat. aq. $NaHCO_3$. The water layer was extracted six times with DCM. The combined organics were dried over $MgSO_4$, filtered

and concentrated *in vacuo*. After purification by flash column chromatography in (0.5 -> 5% MeOH in DCM) all fractions containing titled compound were combined and concentrated *in vacuo.* The residue was taken up in DCM and washed with 10% aqueous citric acid. The water layer was extracted twice with DCM and the combined organics were dried over $MgSO_4$, filtered and evaporated *in vacuo* yielding titled compound as an off-white foam (943 mgram, 0.988 mmol, 91%). **Rf:** 0.10 (25% EtOAc in pentane + 0.1% AcOH). $[\alpha]_D^{20}$ = +62.8° (c = 1.0, $CHCl_3$). **^{1}H NMR** (400 MHz, $CDCl_3$) δ 7.67 (d, *J* = 7.7 Hz, 2H, Fmoc arom.), 7.58 – 7.45 (m, 6H, Fmoc arom. + TBDPS arom.), 7.40 – 7.09 (m, 14H, Fmoc arom. + TBDPS arom. + PMB arom.), 6.81 (t, *J* = 8.3 Hz, 4H, PMB arom.), 6.59 (d, *J* = 8.3 Hz, 1H, NH), 5.40 (d, *J* = 5.4 Hz, 1H, H-1), 4.75 – 4.42 (m, 5H, CH Cys + 2x CH_2 PMB), 4.39 – 4.24 (m, 2H, H-4 + CH_{2a} Fmoc), 4.20 – 3.96 (m, 4H, CH_{2b} Fmoc + H-2 + H-3 + CH Fmoc), 3.81 – 3.67 (m, 7H, 2x CH_3 PMB + H-5_a), 3.60 (dd, *J* = 11.5, 2.9 Hz, 1H, H-5_b), 3.46 – 3.36 (m, 1H, CH_{2a} Cys), 2.96 (dd, *J* = 14.3, 4.0 Hz, 1H, CH_{2b} Cys), 0.93 (s, 9H, *t*Bu TBDPS). **^{13}C NMR** (101 MHz, $CDCl_3$) δ 176.4 (C=O COOH), 159.4, 159.2 (Cq PMB), 156.5 (C=O Fmoc), 143.9, 143.8, 141.2 (Cq Fmoc), 135.6, 135.5 (CH arom. TBDPS), 133.1, 133.0 (Cq TBDPS), 130.1 (Cq PMB), 129.8, 129.8 (CH arom.), 129.6 (Cq PMB), 129.5, 127.7, 127.7, 127.6, 127.1, 127.1, 125.3, 125.2 (CH arom.), 119.9 (CH arom. Fmoc), 113.9, 113.8 (CH arom. PMB), 89.4 (C-1), 82.7 (C-4), 78.1 (C-2), 76.1 (C-3), 72.7, 72.2 (CH_2 PMB), 67.2 (CH_2 Fmoc), 63.4 (C-5), 55.3 (CH_3 PMB), 54.3 (CH Cys), 47.0 (CH Fmoc), 34.5 (CH_2 Cys), 26.8 (CH_3 *t*Bu), 19.2 (Cq *t*Bu). **HRMS:** $[C_{55}H_{59}NO_{10}SSi + Na]^+$ found: 976.3520, calculated: 976.3521.

2',3',5'-tri-*O*-*tert*-butyldimethylsilyl adenosine (S7)

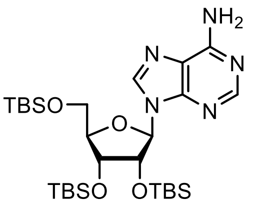

Adenosine (2.67 g, 10.0 mmol) was co-evaporated thrice with anhydrous pyridine (3 x 10 mL), dissolved in anhydrous dimethylformamide (20 mL, 0.2M) and heated to 50 °C, whereupon imidazole (3.40 g, 50.0 mmol, 5.0 eq.) and *tert*-butyldimethylsilyl chloride (50 wt. % in toluene, 12.2 mL, 35.0 mmol, 3.5 eq.) were added consecutively. After stirring overnight, excess silyl chloride was quenched by the addition of H_2O (10 mL), the mixture diluted with Et_2O (100 mL) and washed once with H_2O (100 mL). The aqueous layer was back-extracted twice with Et_2O (2 x 100 mL) and the resulting organic layers combined, washed with sat. aq. NaCl (100 mL), dried over $MgSO_4$, filtered and concentrated *in vacuo*. Purification by column chromatography (20 -> 30% EtOAc in toluene) afforded titled compound as a crystalline white solid (6.11 g, 10.0 mmol, quant.). Spectral data was in accordance with literary precedence.[10] **Rf:** 0.19 (20% EtOAc in toluene). **^{1}H NMR** (400 MHz, $CDCl_3$): δ 8.35 (s, 1H, H-2), 8.18 (s, 1H, H-8), 6.30 (s, 2H, 6-NH2), 6.05 (d, 1H, *J* = 5.2 Hz, H-1'), 4.70 (dd, 1H, *J* = 5.1, 4.3 Hz, H-2'), 4.33 (app. t, 1H, *J* = 3.9 Hz, H-3'), 4.14 (td, 1H, *J* = 3.9, 2.8 Hz, H-4'), 4.05 (dd, 1H, *J* = 11.3, 4.2 Hz, H-5'), 3.80 (dd, 1H, *J* = 11.3, 2.9 Hz, H-5'), 0.96 (s, 9H, CH_3 *t*Bu), 0.94 (s, 9H, CH_3 *t*Bu), 0.81 (s, 9H, CH_3 *t*Bu), 0.16 – 0.13 (m, 6H, CH_3 SiMe), 0.12 – 0.10 (m, 6H, CH_3 SiMe), -0.03 (s, 3H, CH_3 SiMe), -0.21 (s, 3H, CH_3 SiMe). **^{13}C NMR** (101 MHz, $CDCl_3$): δ 155.8 (C-6), 153.0 (C-2), 150.0 (C-4), 139.7 (C-8), 120.1 (C-5), 88.4 (C-1'), 85.5 (C-4'), 75.9 (C-2'), 72.1 (C-3'), 62.6 (C-5'), 26.2, 26.0, 25.8 (*t*Bu TBS), 18.6, 18.2, 18.0 (Cq *t*Bu), -4.3, -4.6, -4.6, -5.0, -5.3 (SiMe).

N^6-*tert*-butyloxycarbonyl-2',3',5'-tri-*O*-*tert*-butyldimethylsilyl adenosine (S8)

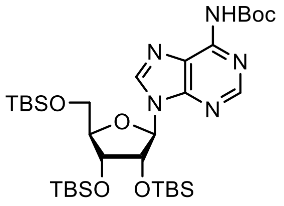

Persilylated adenosine **S7** (5.40 g, 8.86 mmol) was co-evaporated thrice with anhydrous 1,4-dioxane (3 x 10 mL), dissolved in anhydrous tetrahydrofuran (89 mL, 0.1 M) and cooled to 0 °C, whereupon DMAP (217 mg, 1.77 mmol, 0.2 eq.) and di-*tert*-butyl dicarbonate (6.1 mL, 26.6 mmol, 3.0 eq.) were added consecutively. The mixture was heated to reflux and stirred for 2.5 hours, whereafter it was cooled to room temperature and concentrated to dryness. The residue was partitioned betwixt EtOAc (100 mL) and sat. aq. NaCl (100 mL) and the organic layer dried over $MgSO_4$, filtered and evaporated *in vacuo*. The preceding red oil was redissolved in methanol (89 mL, 0.1 M) and cooled in an ice bath, whereupon methylamine (33 wt. % in EtOH, 3.3 mL, 35.4 mmol, 4.0 eq.) was added. After stirring overnight while gradually warming to ambient temperature, the resulting solution was concentrated *in vacuo*. Flash column chromatography (0 -> 30% EtOAc in pentane) furnished titled compound as a crystalline white solid (6.29 g, 8.66 mmol, quant.). Spectral data was in accordance with literary precedence.[10] **Rf:** 0.84 (20% EtOAc in toluene). **^{1}H NMR** (400 MHz, $CDCl_3$): δ 8.74 (s, 1H, H-2), 8.33 (s, 1H, H-8), 8.22 (s, 1H, 6-NH), 6.09 (d, 1H, *J* = 5.3 Hz, H-1'), 4.65 (dd, 1H, *J* = 5.3, 4.3 Hz, H-2'), 4.29 (dd, 1H, *J* = 4.3, 3.4 Hz, H-3'), 4.14 (app. q, 1H, *J* = 3.5 Hz, H-4'), 4.02 (dd, 1H, *J* = 11.4, 3.9 Hz, H-5'), 3.80 (dd, 1H, *J* = 11.4, 2.7 Hz, H-5'), 1.55 (s, 9H, CH3 *t*Bu Boc), 0.96 (s, 9H, CH_3 *t*Bu), 0.93 (s, 9H, CH_3 *t*Bu), 0.78 (s, 9H, CH_3 *t*Bu), 0.15 (s, 3H, CH_3 SiMe), 0.13 (s, 3H, CH_3 SiMe), 0.10 – 0.09 (m, 6H, CH_3 SiMe), -0.06 (s, 3H, CH_3 SiMe), -0.29 (s, 3H, CH_3 SiMe); 13C-APT NMR (101 MHz, CDCl3, HSQC, HMBC): δ 153.0 (C-2), 150.9 (C-6), 149.9 (C-4), 149.8 (C=O), 141.4 (C-8), 122.1 (C-5), 88.4 (C-2'), 85.8 (C-3'), 82.2 (Cq *t*Bu Boc), 76.2 (C-1'), 72.1 (C-4'), 62.7 (C-5'), 28.2 (*t*Bu Boc), 26.1, 25.9, 25.7 (*t*Bu TBS), 18.6, 18.1, 17.9 (Cq *t*Bu), -4.4, -4.6, -4.6, -5.1, -5.3, -5.3 (SiMe).

N^6-*tert*-butyloxycarbonyl-2',3'-di-*O*-*tert*-butyldimethylsilyl adenosine (18)

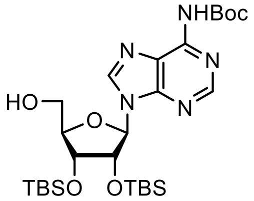

To an ice-cooled solution of N^6-*tert*-butyloxycarbonyl-2',3',5'-tri-*O*-*tert*-butyldimethylsilyl adenosine **S8** (6.29 g, 8.86 mmol) in tetrahydrofuran (89 mL, 0.1 M), a freshly prepared TFA/H2O mixture (1/1; v/v, 65.8 mL, 50 eq.) was gradually added. After stirring for 4.5 hours at 0 °C, the solution was quenched by the careful addition of solid $NaHCO_3$ until pH ~ 7, diluted with sat. aq. $NaHCO_3$ (300 mL) and extracted thrice with EtOAc (3 x 200 mL). The resulting organic layers were combined, dried over MgSO4 filtered and concentrated *in vacuo*. Flash column chromatography (30% -> 50% EtOAc in pentane) furnished titled compound as a crystalline white foam (4.98 g, 8.36 mmol, 94%). Spectral data was in accordance with literary precedence.[10] **Rf**: 0.79 (50% EtOAc in pentane). **^{1}H NMR** (400 MHz, $CDCl_3$): δ 8.75 (s, 1H, H-2), 8.32 (s, 1H, 6-NH), 7.99 (s, 1H, H-8), 6.24 (br. s, 1H- 5'-OH), 5.83 (d, 1H, *J* = 7.8 Hz, H-1'), 5.03 (dd, 1H, *J* = 7.8, 4.6 Hz, H-2'), 4.34 (d, 1H, *J* = 4.5 Hz, H-3'), 4.18 (d, 1H, *J* = 1.7 Hz, H-4'), 3.96 (dd, 1H, *J* = 13.1, 1.8 Hz, H-5'), 3.73 (d, 1H, *J* = 13.1 Hz, H-5'), 1.57 (s, 9H, CH_3 *t*Bu Boc), 0.95 (s, 9H, CH_3 *t*Bu), 0.73 (s, 9H, CH_3 *t*Bu), 0.14 – 0.12 (m, 6H, CH_3 SiMe), -0.14 (s, 3H, CH_3 SiMe), -0.66 (s, 3H, CH_3 SiMe). **^{13}C NMR** (101 MHz, $CDCl_3$): δ 152.5 (C-2), 150.8 (C-6), 149.7 (C=O), 149.5 (C-4), 142.9 (C-8), 123.3 (C-5), 91.2 (C-1'), 89.7 (C-4'), 82.6 (Cq *t*Bu Boc), 74.0 (C-2'), 74.0 (C-3'), 63.0 (C-5'), 28.2 (*t*Bu Boc), 25.9, 25.7 (*t*Bu TBS), 18.1, 17.8 (Cq *t*Bu), -4.5, -4.5, -4.6, -5.9 (SiMe).

5'-*O*-(N^6-*tert*-butyloxycarbonyl-2',3'-di-*O*-*tert*-butyldimethylsilyladenosine)-2-cyanoethyl-*N*-*N*-diisopropylphosphoramidite (6)

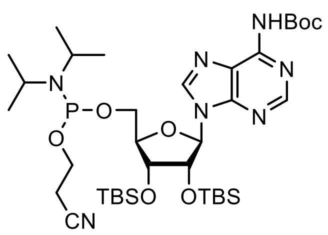

N^6-*tert*-butyloxycarbonyl-2',3'-di-*O*-*tert*-butyldimethylsilyl adenosine **18** (1.49 g, 2.5 mmol) was co-evaporated thrice with toluene and dissolved in anhydrous DCM (25 mL, 0.1M). DIPEA (1.34 mL, 7.5 mmol, 3.0 eq.) and 2-cyanoethyl-*N*,*N*-diisopropylchlorophosphoramidite (0.61 mL, 2.75 mmol, 1.1 eq.) were added to the reaction and after 3 hours TLC indicated full conversion of starting material. The reaction was diluted with DCM and washed with brine. The organic layer was dried over $MgSO_4$, filtered and evaporated *in vacuo*. Flash column chromatography (20 -> 30% EtOAc in pentane + 1% TEA) afforded titled compound as a white foam and a mixture of diastereomers (S_P/R_P). (1.77 g, 2.23 mmol, 89%). **Rf:** 0.76 (40% EtOAc in pentane). **^{1}H NMR** (400 MHz, $CDCl_3$) δ 8.76 – 8.67 (m, 1H, H-2), 8.36 – 8.29 (m, 1H, H-8), 8.03 (s, 1H, 6-NH), 6.04 (dd, *J* = 15.4, 5.3 Hz, 1H, H-1'), 4.86 – 4.67 (m, 1H, H-2'), 4.36 – 4.18 (m, 2H, H-3' + H-4'), 4.12 – 3.75 (m, 4H, H-5' + O$CH_2$$CH_2$CN), 3.62 (m, 3H, H-5' + 2x RNC*H*(CH_3)$_2$), 2.68 (m, 2H, OCH_2C*H_2*CN), 1.55 (s, 9H), 1.20 (dq, *J* = 8.5, 3.6, 3.0 Hz, 12H, 2x RNCH(C*H_3*)$_2$), 0.92 (m, 9H, CH_3 *t*Bu), 0.79 – 0.74 (m, 9H, CH_3 *t*Bu), 0.14 – 0.03 (m, 6H, CH_3 SiMe), -0.02 – -0.14 (m, 3H, CH_3 SiMe), -0.18 – -0.39 (m, 3H, CH_3 SiMe). **^{13}C NMR** (101 MHz, $CDCl_3$) δ 153.0, 152.9 (C-2), 151.0, 150.8 (C-6), 149.9, 149.9, 149.8 (C=O + C-4), 141.8 (C-8), 122.3, 122.2 (C-5), 117.5, 117.5 (Cq OCE), 89.1, 88.4 (C-1'), 85.2, 85.1, 84.4, 84.3 (C-4'), 82.3, 82.3 (Cq *t*Bu Boc), 75.6, 75.5 (C-2'), 73.0, 72.0 (C-3'), 63.0, 62.8, 62.2, 62.0 (C-5'), 58.8, 58.7, 58.6, 58.5 (O*C*$H_2$$CH_2$CN), 43.3, 43.2, 43.2, 43.1 (RN*C*H(CH_3)$_2$), 28.2 (*t*Bu Boc), 25.9, 25.9, 25.8, 25.8 (*t*Bu TBS), 24.9, 24.9, 24.8, 24.8, 24.7 (RNCH(*C*H_3)$_2$), 20.5, 20.5, 20.5 (OCH_2*C*H_2CN), 18.2, 18.1, 17.9 (Cq *t*Bu), -4.3, -4.4, -4.6, -4.6, -4.6, -4.9, -5.1 (SiMe). **^{31}P NMR** (162 MHz, $CDCl_3$) δ 149.8, 149.7. **HRMS:** *mass was detected as its corresponding H-phosphonate* [$C_{30}H_{53}N_6O_8PSi_2$ + H]$^+$ found: 713.3272, calculated: 713.3274.

Solid phase synthesis

Peptide synthesis

The intermediate peptides were synthesized using standard, Fmoc-based solid phase peptide synthesis utilizing (pre-loaded) Tentagel® S AC purchased from Rapp Polymer Gmbh. Coupling cycles were as followed: Fmoc deprotection: 2x2 min, 1x5 min treatment with 20% piperidine in DMF. Coupling: treatment of 6 eq. amino acid, 6 eq. HCTU (0.25M in DMF) and 12 eq. DIPEA (1M in DMF) for 30 minutes. Capping: 2x2 min treatment of the resin with a 10% Ac_2O solution in DMF and catalytic DIPEA. Washing between the steps was done with DMF. Ribosylated amino acids **1**, **2** and **3** were incorporated in the sequence by adding a solution of 2 eq. building block in a 0.25M HCTU solution (2 eq.) in DMF and a 1M DIPEA solution (4 eq.) in DMF to the resin in a fritted syringe. The resin was shaken overnight and thoroughly washed.

On-resin phosphorylation

The resin was treated with a sufficient amount of 1M TBAF in THF for 30 minutes. The resin was thoroughly washed with DCM and DMF before the treatment was repeated once, furnishing the desilylated intermediate. The resin was then extensively washed with MeCN and flushed with nitrogen to remove traces of water before the resin was subjected to a solution of 5 eq. of $(FmO)_2PN(iPr)_2$ (0.25M in MeCN) with 10 eq. ETT solution (0.25M in MeCN). The resin was shaken for 30 minutes after which the resin was washed with MeCN. The resin

was then treated with a sufficient amount of CSO solution (0.5M in MeCN) for 30 minutes. The resin was then treated with a 10% DBU solution in DMF (2x 15 minutes) to furnish the crude, immobilized and deprotected phosphoribosylated peptide.

Construction of the pyrophosphates

The resin was extensively washed with MeCN and flushed with nitrogen to remove traces of water. The resin was then treated with a solution of 5'-*O*-(N^6-*tert*-butyloxycarbonyl-2',3'-di-*O*-*tert*-butyldimethylsilyladenosine)-2-cyanoethyl-*N*-*N*-diisopropylphosphoramidite **6** (3 eq., 0.3M in MeCN) and ETT (6 eq., 0.25M in MeCN) for 30 minutes. The resin was thoroughly washed with MeCN before a sufficient amount of CSO (0.5M in MeCN) was added to the resin and shaken for 30 minutes.

Final deprotection and cleavage

The resin was then treated with a 10% DBU solution in DMF (2x 10 minutes) to remove the cyano ethyl protecting group. The resin was then treated with a 1M TBAF solution in THF (2x 45 minutes) and washed with DMF followed by DCM. Final cleavage/deprotection occurred by treating the resin with a cleavage cocktail (2.5/10/87.5 TIS/TFA/DCM) for 4 hours. The crude products were collected by filtration and the resin was washed with a solution of 1/1/1 water/*t*BuOH/MeCN. The solvents were evaporated *in vacuo* and co-evaporated with a 1/1/1 water/*t*BuOH/MeCN solution.

Ac-Pro-Ala-Lys-Ser(5-O-adenosine diphosphate-α-D-ribosyl)-Ala-Pro-Ala-Pro-Lys-Lys-Gly-OH (24)

The above described procedures were applied to 25 µmol Tentagel® S AC resin preloaded with glycine. The amino acids used were: Fmoc-Pro-OH, Fmoc-Ala-OH, Fmoc-Lys(Mtt)-OH and **7**. The crude peptide was purified by RP-HPLC in NH_4OAc buffer. The pure fractions were concentrated, co-eavporated extensively with a 1:1 mixture of MeCN:Milli-Q water, redissolved in Milli-Q water and lyophilized to obtain titled compound as a white solid (1.49 mgram, 0.89 µmol, 3.6%). **^{1}H NMR** (850 MHz, D_2O) δ 8.48 (s, 1H, H-2 adenine), 8.23 (s, 1H, H-8 adenine), 6.10 (d, J = 6.0 Hz, 1H, H-1' adenosine), 4.94 (d, J = 3.5 Hz, 1H, H-1' ribosyl). **^{31}P NMR** (202 MHz, D_2O) δ -11.21, -11.31, -11.35, -11.46. **LC-MS** (0 -> 50% B in A): Rt = 3.61. **HRMS:** $[C_{64}H_{105}N_{19}O_{27}P_2 + 2H]^{2+}$ found:817.8522, calculated: 817.8524.

Ac-Gly-Lys-Ser(5-*O*-adenosine-diphosphate-α-D-ribosyl)-Gly-Ala-Ala-Leu-Ser-Lys-Lys-Gly-OH (25)

The above described procedures were applied to 50 µmol Tentagel® S AC resin preloaded with glycine. The amino acids used were: Fmoc-Glyc-OH, Fmoc-Lys(Mtt)-OH, Fmoc-Ala-OH, Fmoc-Leu-OH, Fmoc-Ser(Trt)-OH and **7**. The crude peptide was purified by RP-HPLC in NH_4OAc buffer. The pure fractions were concentrated, co-eavporated extensively with a 1:1 mixture of MeCN:Milli-Q water, redissolved in Milli-Q water and lyophilized to obtain titled compound as a white solid (8.87 mgram, 5.46 µmol, 11%). **^{1}H NMR** (500 MHz, D_2O) δ 8.50 (s, 1H, H-2 adenine), 8.25 (s, 1H, H-8 adenine), 6.13 (d, *J* = 5.9 Hz, 1H, H-1' adenosine), 4.99 (d, *J* = 3.6 Hz, 1H, H-1' ribosyl). **^{31}P NMR** (202 MHz, D_2O) δ -10.5, -10.6, -10.7, -10.8. **LC-MS** (0 -> 20% B in A): Rt = 8.54. **HRMS:** $[C_{59}H_{101}N_{19}O_{28}P_2 + 2H]^{2+}$ found: 793.8334, calculated: 793.8342.

Ac-Gly-Lys-Ser-(5-*O*-adenosine-diphosphate-α-D-ribosyl)-Ser-Gly-Pro-Thr-Ser-Leu-Phe-Ala-Val-Thr-Val-Ala-Pro-Pro-Gly-Ala-Arg-Gly-OH (26)

The above described procedures were applied to 50 µmol Tentagel® S AC resin preloaded with glycine. The amino acids used were: Fmoc-Gly-OH, Fmoc-Lys(Mtt)-OH, Fmoc-Ser(Trt)-OH, Fmoc-Pro-OH, Fmoc-Thr(Trt)-OH, Fmoc-Leu-OH, Fmoc-Phe-OH, Fmoc-Ala-OH, Fmoc-Val-OH, Fmoc-Arg(Alloc)$_2$-OH and **7**. The Alloc protecting group was removed by treating the resin with a freshly prepared solution of 10 mg Pd(PPh$_3$)$_4$ and 23 mg DMBA in 2.5 mL DCM. This procedure was then repeated twice prior to cleavage and final deprotection of remaining protecting groups. The crude peptide was purified by RP-HPLC in NH_4OAc buffer. The pure fractions were concentrated, co-eavporated extensively with a 1:1 mixture of MeCN:Milli-Q water, redissolved in Milli-Q water and lyophilized to obtain titled compound as a white solid (7.75 mgram, 3.05 µmol, 6.1%). **^{1}H NMR** (850 MHz, D_2O) δ 8.48 (s, 1H, H-2 adenine), 8.22 (s, 1H, H-8 adenine), 7.29 (t, J = 7.5 Hz, 2H, Phe arom.), 7.24 (t, J = 7.4 Hz, 1H, Phe arom.), 7.18 (d, J = 7.4 Hz, 2H, Phe arom.), 6.09 (d, J = 6.0 Hz, 1H, H-1′ adenosine), 4.96 (d, J = 3.4 Hz, 1H, H-1′ ribosyl). **^{31}P NMR** (202 MHz, D_2O) δ -10.51, -10.62, -10.67, -10.77. **LC-MS** (10 -> 90% B in A): Rt = 3.58. **HRMS:** $[C_{103}H_{164}N_{30}O_{41}P_2 + 2H]^+$ found: 1270.5652, calculated: 1270.5646.

Ac-Gly-Lys-Ser-Ser-Gly-Pro-Thr(5-*O*-adensoine-diphosphate-α-D-ribosyl)-Ser-Leu-Phe-OH (27)

The above described procedures were applied to 50 µmol Tentagel® S AC resin preloaded with phenylalanine. The amino acids used were: Fmoc-Gly-OH, Fmoc-Lys(Mtt)-OH, Fmoc-Ser(Trt)-OH, Fmoc-Pro-OH, Fmoc-Thr(Trt)-OH, Fmoc-Leu-OH and **20**. The crude peptide was purified by RP-HPLC in NH_4OAc buffer. The pure fractions were concentrated, co-eavporated extensively with a 1:1 mixture of MeCN:Milli-Q water, redissolved in Milli-Q water and lyophilized to obtain titled compound as a white solid (7.57 mgram, 4.74 µmol, 9.5%). **^{1}H NMR** (850 MHz, D_2O) δ 8.50 (s, 1H, H-2 adenine), 8.21 (s, 1H, H-8 adenine), 7.28 (t, J = 7.5 Hz, 2H, Phe arom.), 7.22 (t, J = 7.3 Hz, 1H, Phe arom.), 7.16 (d, J = 7.0 Hz, 2H, Phe arom.), 6.10 (d, J = 5.6 Hz, 1H, H-1′ adenosine), 4.96 (d, J = 4.5 Hz, 1H, H-1′ ribosyl). **^{31}P NMR** (202 MHz, D_2O) δ -11.15, -11.26, -11.33, -11.44. **LC-MS** (10 -> 90% B in A): Rt = 3.33. **HRMS:** $[C_{60}H_{92}N_{16}O_{29}P_2 + 2H]^+$ found: 782.2905, calculated: 782.2918.

Ac-Lys-Glu-Ser-Thr(5-*O*-adensoine-diphosphate-α-D-ribosyl)-Leu-His-Leu-Val-Leu-Arg-Leu-OH (28)

50 µmol Tentagel S AC resin was loaded by treating the resin with 2.5 mL of a 0.2M Fmoc-Leu-OH solution (10 eq.) and DIC (77 µL, 0.5 mmol, 10 eq.) in DMF together with a catalytic amount of DMAP for 2 hours. The resin was drained and washed with DMF after which the above described procedures were applied. The amino acids used were Fmoc-Lys(Mtt)-OH, Fmoc-Glu(O-2-Ph*i*Pr)-OH, Fmoc-Ser(Trt)-OH, Fmoc-Leu-OH, Fmoc-His(Trt)-OH, Fmoc-Val-OH, Fmoc-Arg(Alloc)$_2$-OH and **20**. The Alloc protecting group was removed by treating the resin with a freshly prepared solution of 10 mg Pd(PPh$_3$)$_4$ and 23 mg DMBA in 2.5 mL DCM. This procedure was then repeated twice prior to cleavage and final deprotection of remaining protecting groups. The crude peptide was purified by RP-HPLC in NH_4OAc buffer. The pure fractions were concentrated, co-eavporated extensively with a 1:1 mixture of MeCN:Milli-Q water, redissolved in Milli-Q water and lyophilized to obtain titled compound as a white solid (0.91 mgram. 0.47 µmol, 0.94%). **^{1}H NMR** (850 MHz, D_2O) δ 8.47 (s, 1H, H-2 adenine), 8.21 (s, 1H, H-8 adenine), 6.09 (d, J = 6.0 Hz, 1H, H-1′ adenosine), 4.95 (d, J = 4.0 Hz, 1H, H-1′ ribosyl). **LC-**

MS (10 -> 90% B in A): Rt = 4.50. **HRMS:** [$C_{76}H_{128}N_{22}O_{30}P_2$ + 2H]$^+$ found: 946.4379, calculated: 946.4394.

Ac-Pro-Ala-Lys-Cys(5-O-adenosine diphosphate-α-D-ribosyl)-Ala-Pro-Ala-Pro-Lys-Lys-Gly-OH (29)

The above described procedures were applied to 50 µmol Tentagel® S AC resin preloaded with glycine. The amino acids used were: Fmoc-Pro-OH, Fmoc-Ala-OH, Fmoc-Lys(Mtt)-OH and **21.** The crude peptide was purified by RP-HPLC in NH_4OAc buffer. The pure fractions were concentrated, co-eavporated extensively with a 1:1 mixture of MeCN:Milli-Q water, redissolved in Milli-Q water and lyophilized to obtain titled compound as a white solid (3.43 mgram, 2.04 µmol, 4.1%). **^{1}H NMR** (850 MHz, D_2O) δ 8.49 (s, 1H, H-2 adenine), 8.23 (s, 1H, H-8 adenine), 6.10 (d, J = 6.1 Hz, 1H, H-1′ adenosine), 5.38 (d, J = 4.8 Hz, 1H, H-1′ ribosyl). **^{31}P NMR** (202 MHz, D_2O) δ -11.11, -11.21, -11.30, -11.40. **LC-MS** (0 -> 50% B in A): Rt = 4.42. **HRMS:** [$C_{64}H_{105}N_{19}O_{26}P_2$ + 2H]$^+$ found: 825.8408, calculated: 825.8410.

Biotin-Pro-Ala-Lys-Cys(5-O-adenosine diphosphate-α-D-ribosyl)-Ala-Pro-Ala-Pro-Lys-Lys-Gly-OH (30)

The above described procedures were applied to 50 µmol Tentagel® S AC resin preloaded with glycine. The amino acids used were: Fmoc-Pro-OH, Fmoc-Ala-OH, Fmoc-Lys(Mtt)-OH and **21.** Oxidation steps were carried out with a 0.5M *t*BuOOH solution in MeCN. The crude peptide was purified by RP-HPLC in NH_4OAc buffer. The pure fractions were concentrated, co-evaporated extensively with a 1:1 mixture of MeCN:Milli-Q water, redissolved in Milli-Q water and lyophilized to obtain titled compound as a white solid (1.73 mgram, 0.93 µmol, 1.9%). **^{1}H NMR** (850 MHz, D_2O) δ 8.49 (s, 1H, H-2 adenine), 8.23 (s, 1H, H-8 adenine), 6.10 (d, J = 6.1 Hz, 1H, H-1′ adenosine), 5.38 (d, J = 4.7 Hz, 1H, H-1′ ribosyl). **LC-MS** (0 -> 50% B in A): Rt = 5.52. **HRMS:** [$C_{72}H_{117}N_{21}O_{27}P_2S_2$ + 2H]$^+$ found: 917.8730, calculated: 917.8745.

References

[1] H. A. V Kistemaker, G. J. Van Der Heden Van Noort, H. S. Overkleeft, G. A. Van Der Marel, D. V. Filippov, *Org. Lett.* **2013**, *15*, 2306–2309.

[2] R. R. Schmidt, J. Michel, *Angew. Chemie Int. Ed. English* **1980**, *19*, 731–732.

[3] M. Ciommer, H. Kunz, *Synlett* **1991**, 593–595.

[4] S. Rio, J. M. Beau, J. C. Jacquinet, *Carbohydr. Res.* **1991**, *219*, 71–90.

[5] P. Fontana, J. J. Bonfiglio, L. Palazzo, E. Bartlett, I. Matic, I. Ahel, *Elife* **2017**, *6*, 1–20.

[6] J. G. M. Rack, R. Morra, E. Barkauskaite, R. Kraehenbuehl, A. Ariza, Y. Qu, M. Ortmayer, O. Leidecker, D. R. Cameron, I. Matic, A. Y. Peleg, D. Leys, A. Traven, I. Ahel, *Mol. Cell* **2015**, *59*, 309–320.

[7] D. Slade, M. S. Dunstan, E. Barkauskaite, R. Weston, P. Lafite, N. Dixon, M. Ahel, D. Leys, I. Ahel, *Nature* **2011**, *477*, 616–622.

[8] J. Voorneveld, J. G. M. Rack, I. Ahel, H. S. Overkleeft, G. A. Van Der Marel, D. V. Filippov, *Org. Lett.* **2018**, *20*, DOI 10.1021/acs.orglett.8b01742.

[9] L. Palazzo, B. Thomas, A. S. Jemth, T. Colby, O. Leidecker, K. L. H. Feijs, R. Zaja, O. Loseva, J. C. Vert, I. Matic, T. Helleday, I. Ahel, *Biochem. J.* **2015**, *468*, 293–301.

[10] C. Atdjian, L. Iannazzo, E. Braud, M. Ethève-Quelquejeu, *European J. Org. Chem.* **2018**, *2018*, 4411–4425.

H-NMR 1-O-(2,3-bis-O-(4-methoxybenzyl)-5-O-((tert-butyl)-diphenylsilyl)-D-ribosyl)-N-fluorenylmethoxycarbonyl — serine allyl ester —

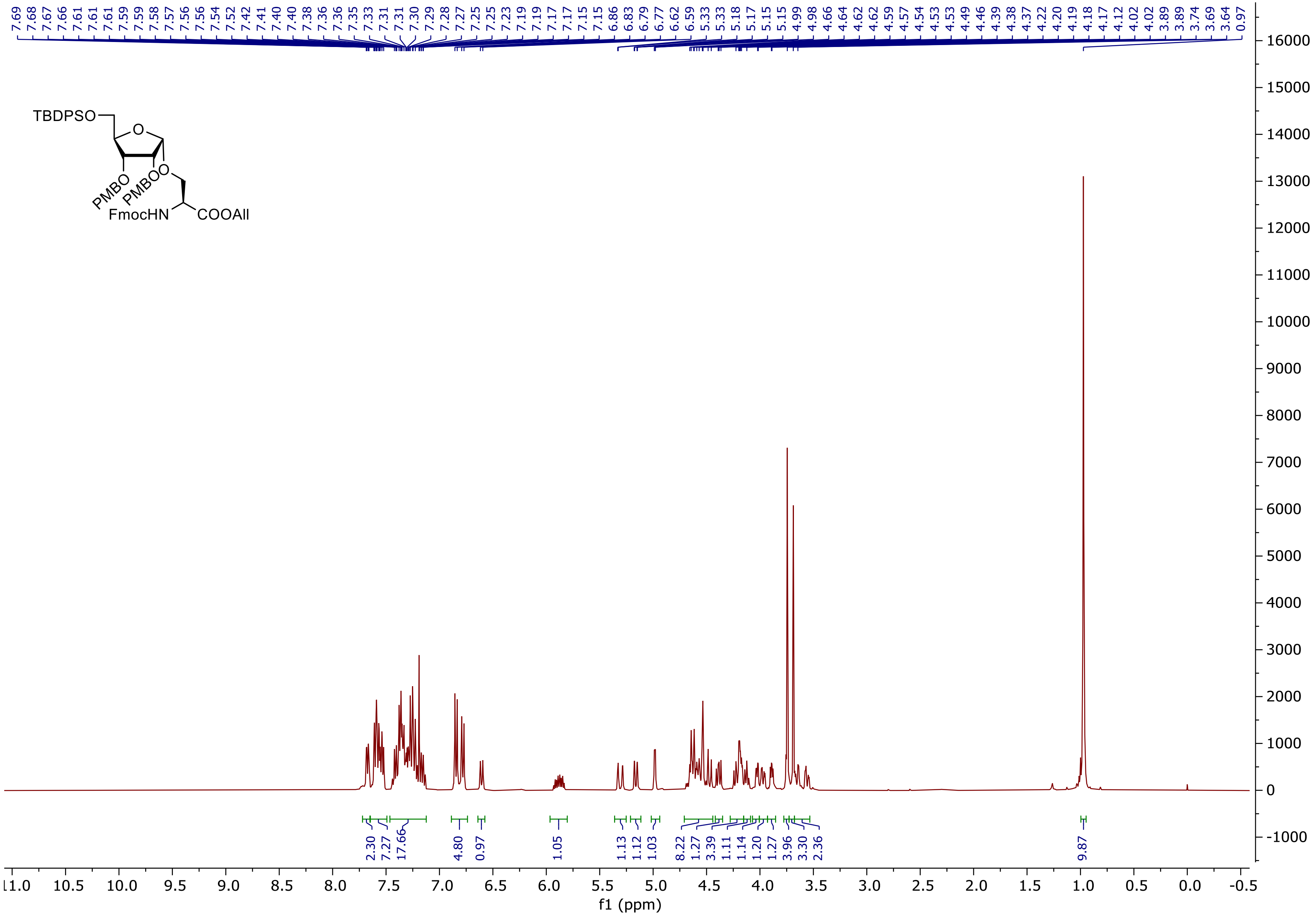

C-NMR 1-O-(2,3-bis-O-(4-methoxybenzyl)-5-O-((tert-butyl)-diphenylsilyl)-D-ribosyl)-N-fluorenylmethoxycarbonyl — serine allyl ester —

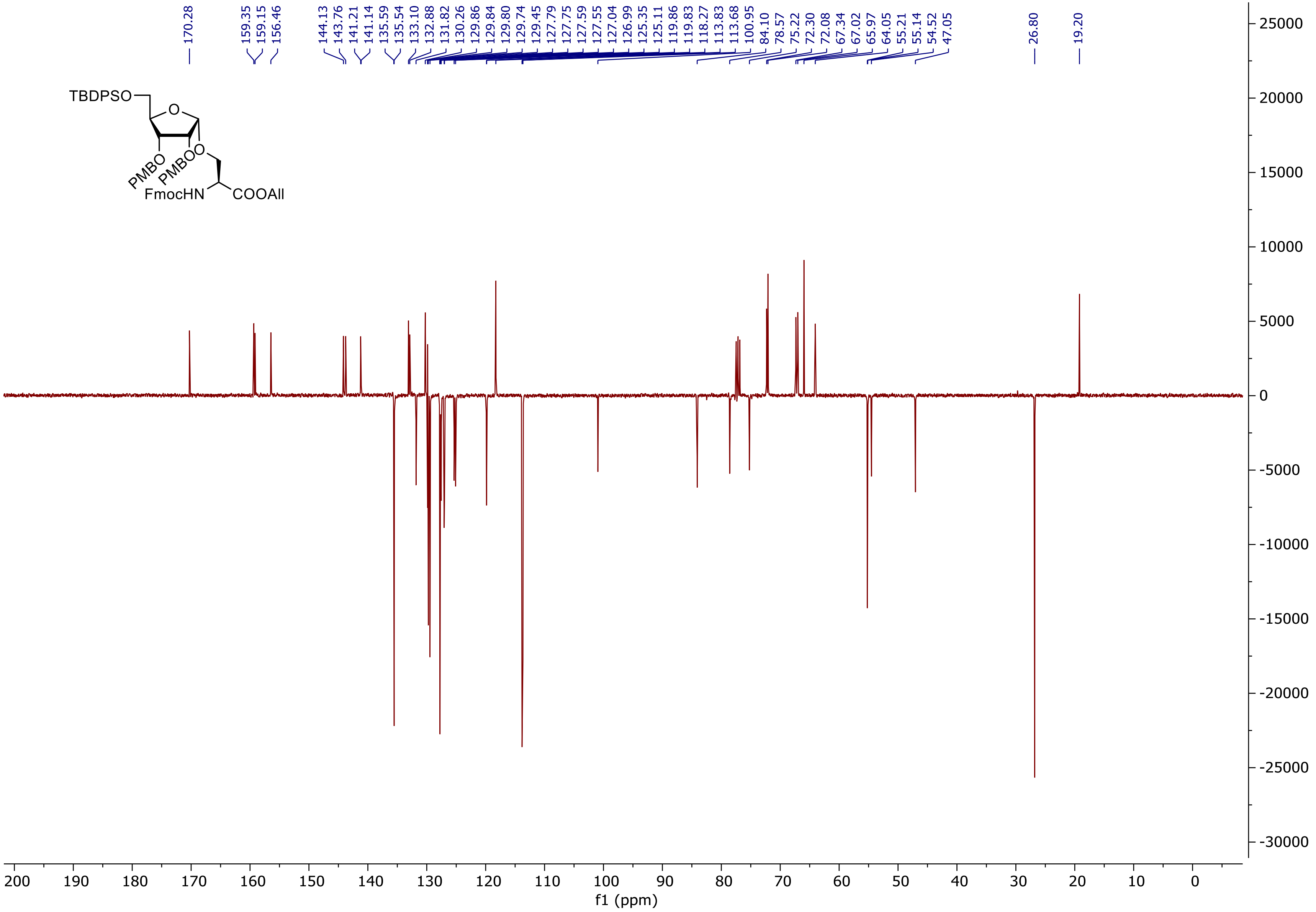

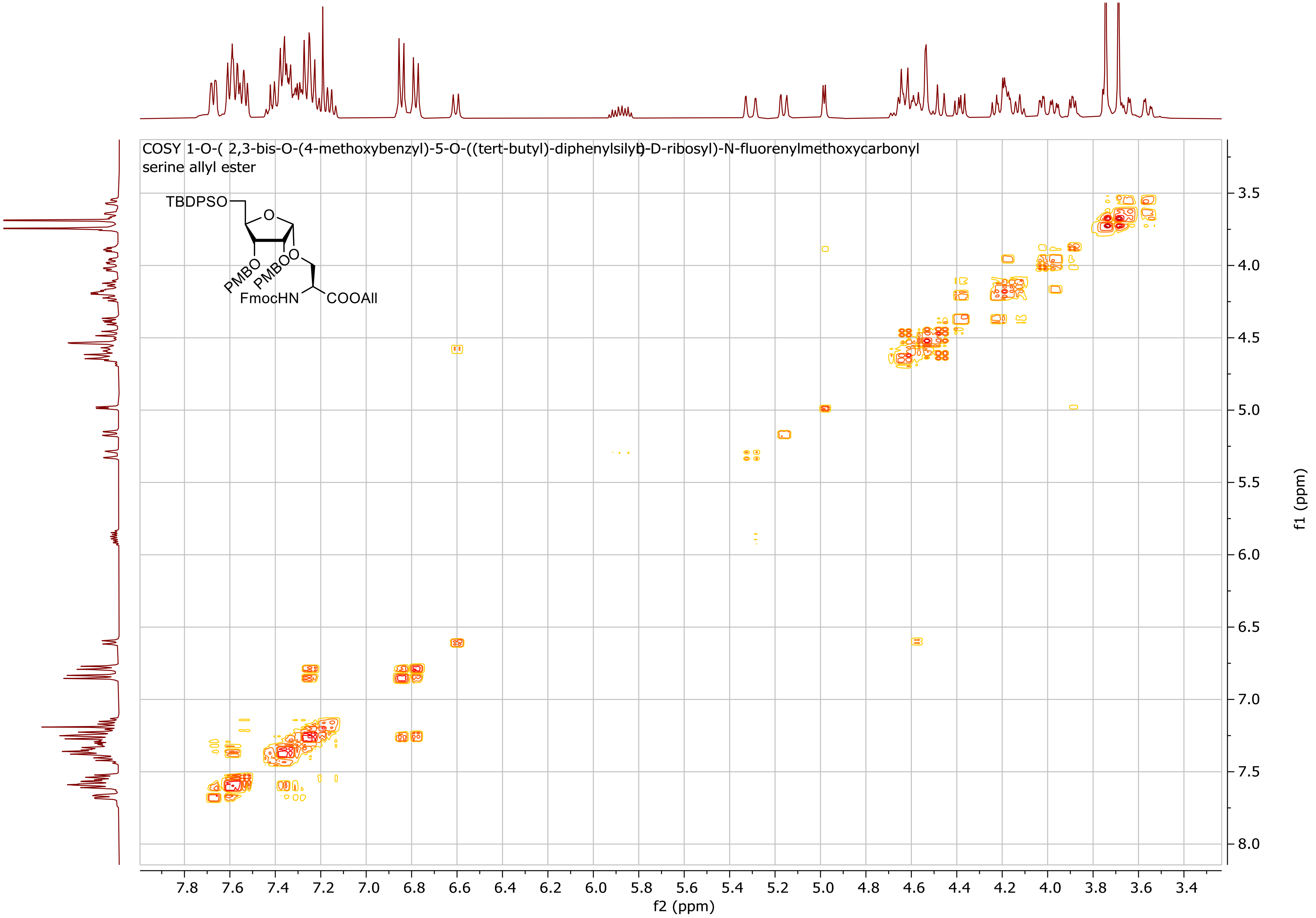
COSY 1-O-(2,3-bis-O-(4-methoxybenzyl)-5-O-((tert-butyl)-diphenylsilyl)-D-ribosyl)-N-fluorenylmethoxycarbonyl
serine allyl ester
TBDPSO
O
PMBO
PMBO
O
FmocHN
COOAll
f2 (ppm)
f1 (ppm)

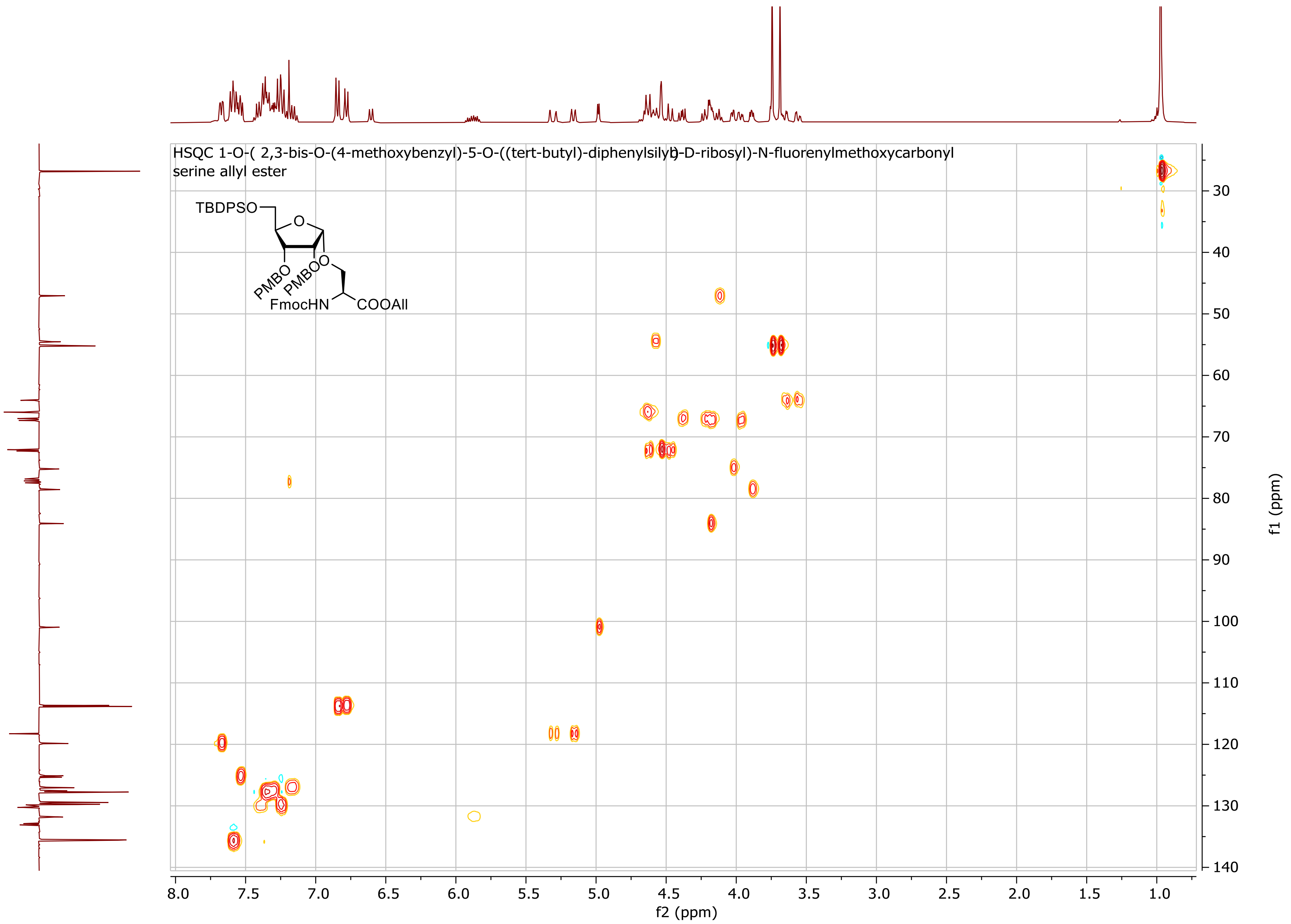
HSQC 1-O-(2,3-bis-O-(4-methoxybenzyl)-5-O-((tert-butyl)-diphenylsilyl)-D-ribosyl)-N-fluorenylmethoxycarbonyl serine allyl ester
TBDPSO
O
PMBO
PMBO
O
FmocHN
COOAll
f2 (ppm)
f1 (ppm)

H-NMR 1-O-(2,3-bis-O-(4-methoxybenzyl)-5-O-((tert-butyl)-diphenylsilyl)-D-ribosyl)-N-fluorenylmethoxycarbonyl — threonine allyl ester —

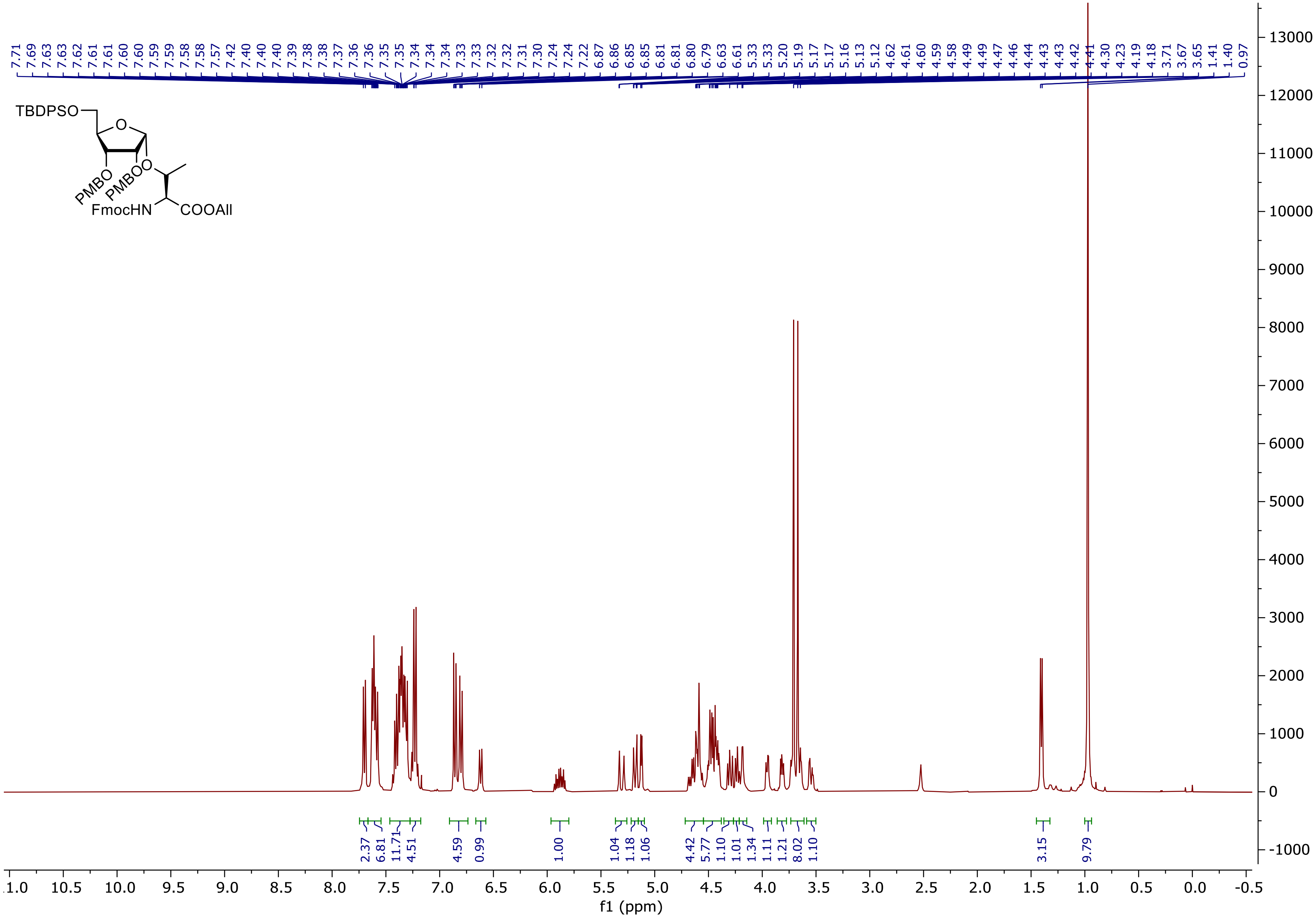

C- NMR 1-O-(2,3-bis-O-(4-methoxybenzyl)-5-O-((tert-butyl)-diphenylsilyl)-D-ribosyl)-N-fluorenylmethoxycarbonyl — threonine allyl ester —

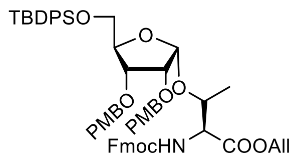

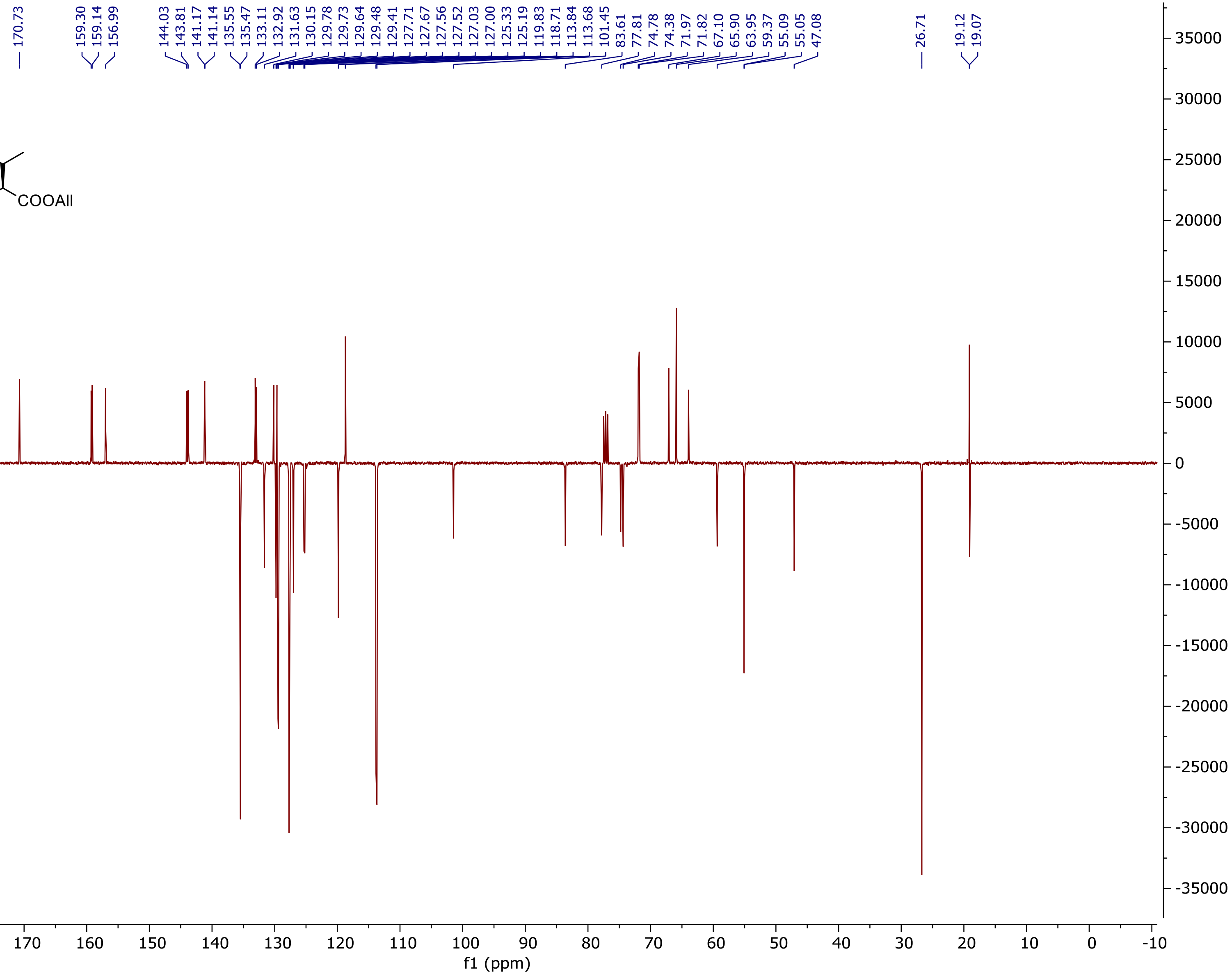

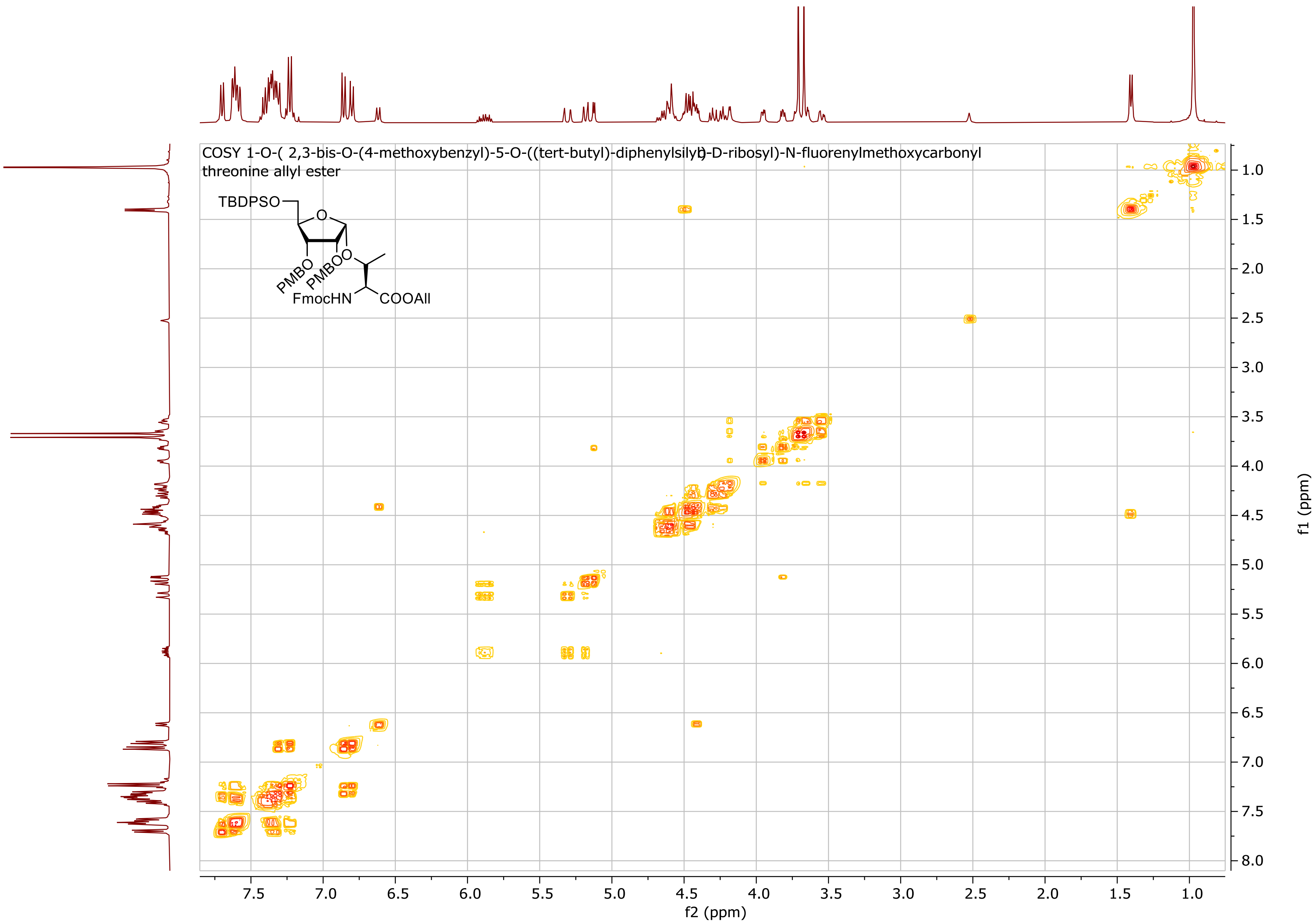
COSY 1-O-(2,3-bis-O-(4-methoxybenzyl)-5-O-((tert-butyl)-diphenylsilyl)-D-ribosyl)-N-fluorenylmethoxycarbonyl threonine allyl ester
TBDPSO
O
PMBO
PMBO
O
FmocHN
COOAll
f2 (ppm)
f1 (ppm)

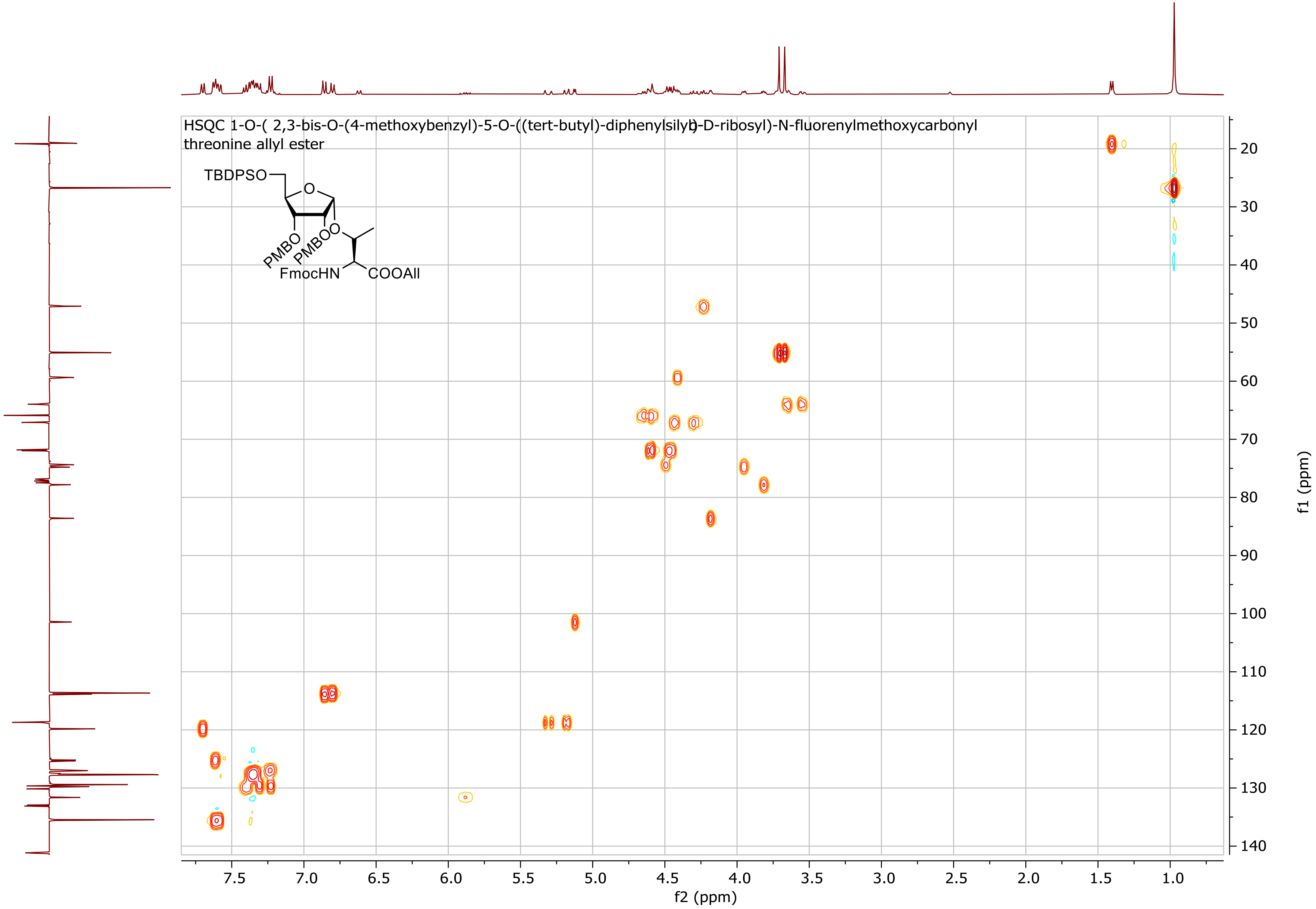
HSQC 1-O-(2,3-bis-O-(4-methoxybenzyl)-5-O-((tert-butyl)-diphenylsilyl)-D-ribosyl)-N-fluorenylmethoxycarbonyl threonine allyl ester
TBDPSO
O
PMBO
PMBO
O
FmocHN
COOAll
f2 (ppm)
f1 (ppm)

H-NMR 1-O-(2,3-bis-O-(4-methoxybenzyl)-5-O-((tert-butyl)-diphenylsilyl)-D-ribosyl)-N-fluorenylmethoxycarbonyl
cysteine allyl ester

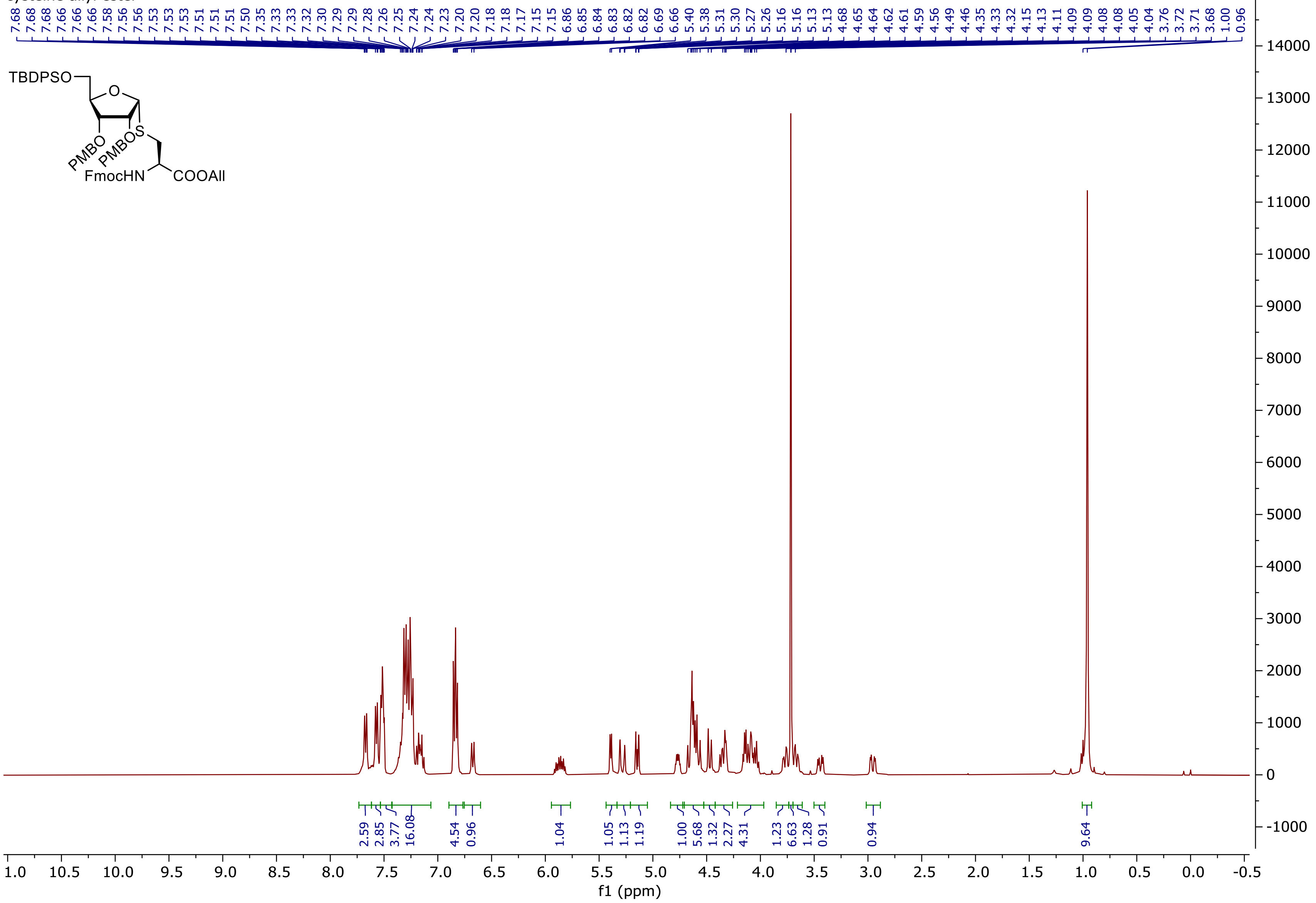

C-NMR 1-O-(2,3-bis-O-(4-methoxybenzyl)-5-O-((tert-butyl)-diphenylsilyl)-D-ribosyl)-N-fluorenylmethoxycarbonyl — cysteine allyl ester

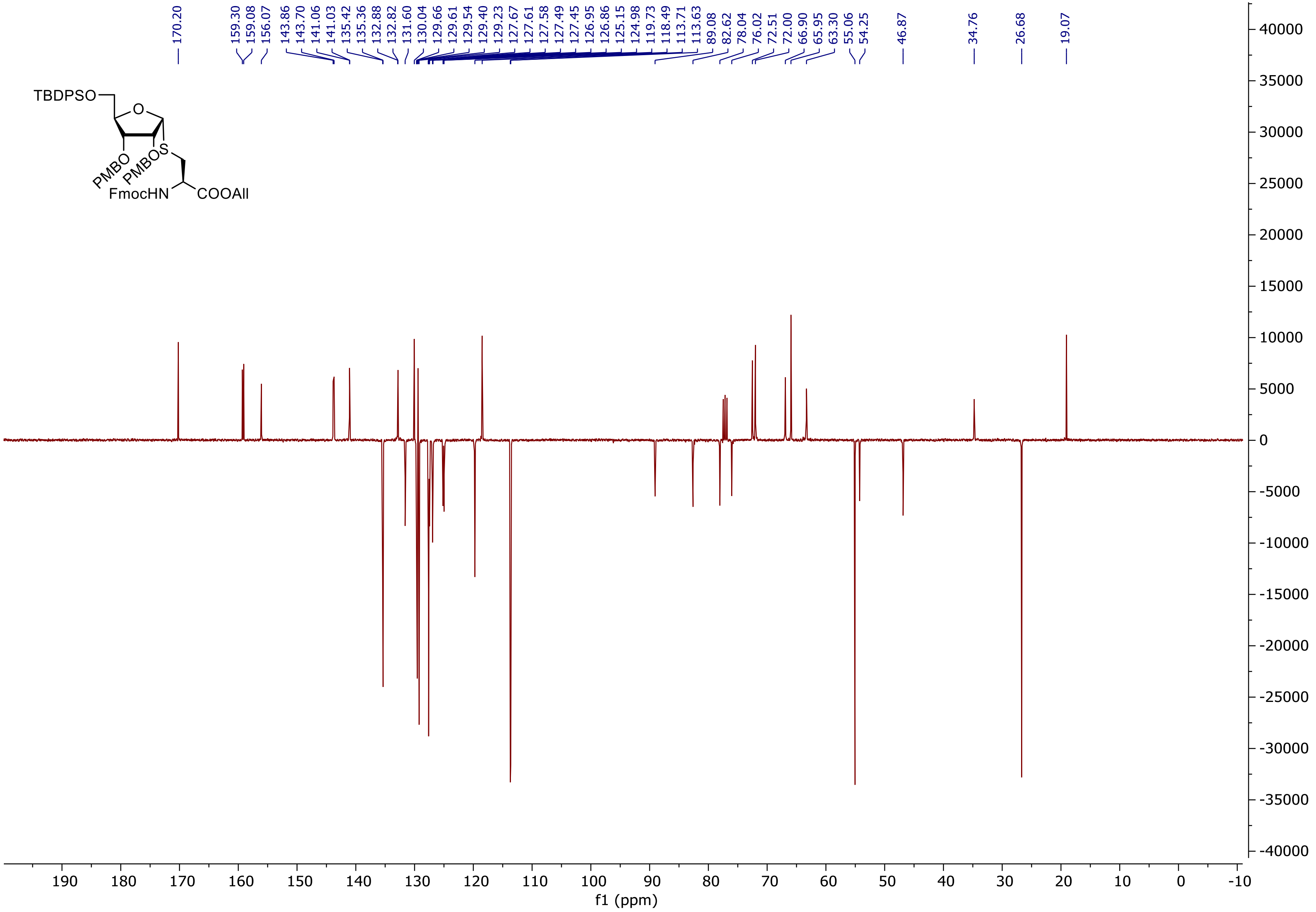

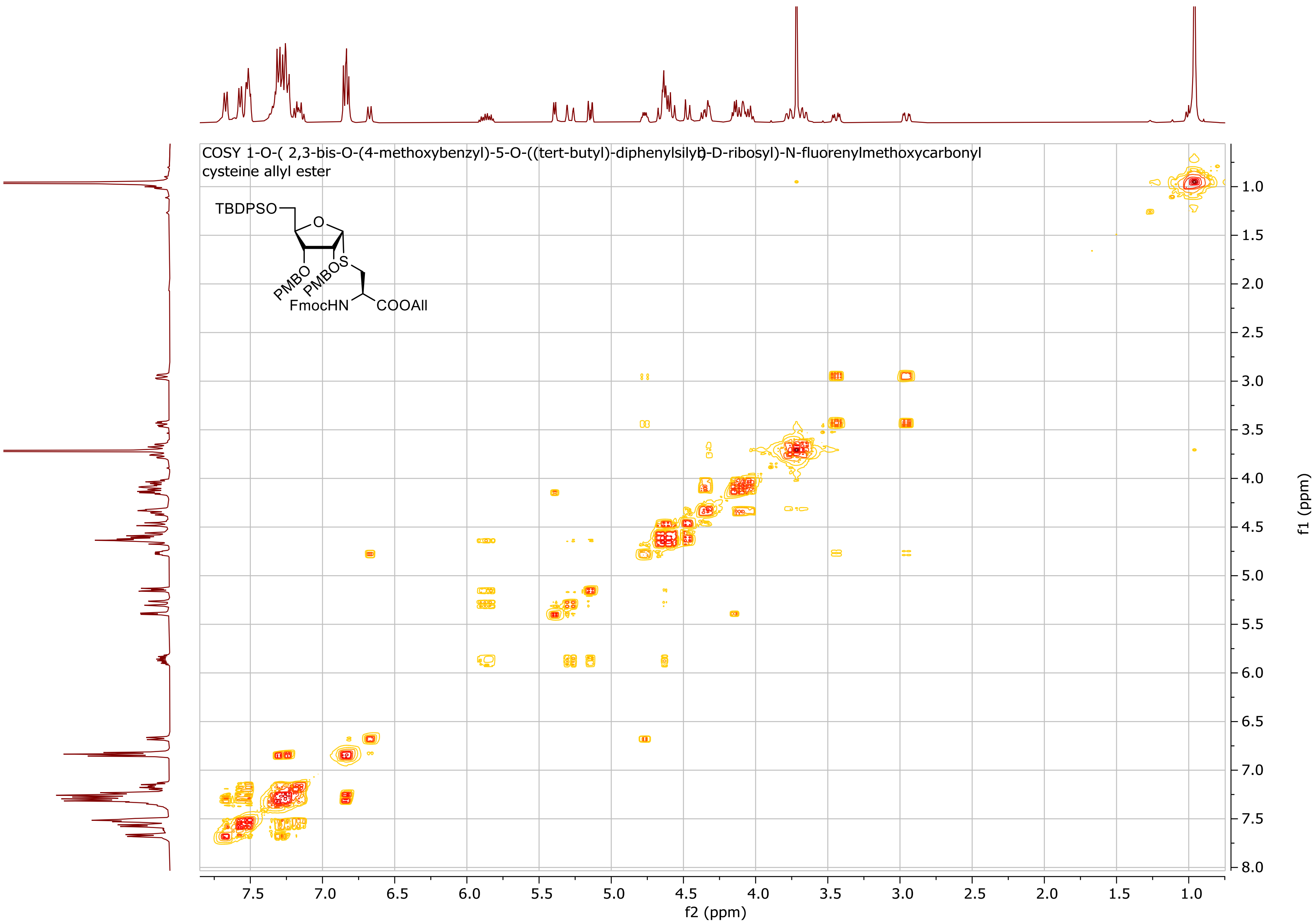
COSY 1-O-(2,3-bis-O-(4-methoxybenzyl)-5-O-((tert-butyl)-diphenylsilyl)-D-ribosyl)-N-fluorenylmethoxycarbonyl cysteine allyl ester
TBDPSO
O
PMBO
PMBO
S
FmocHN
COOAll
f2 (ppm)
f1 (ppm)

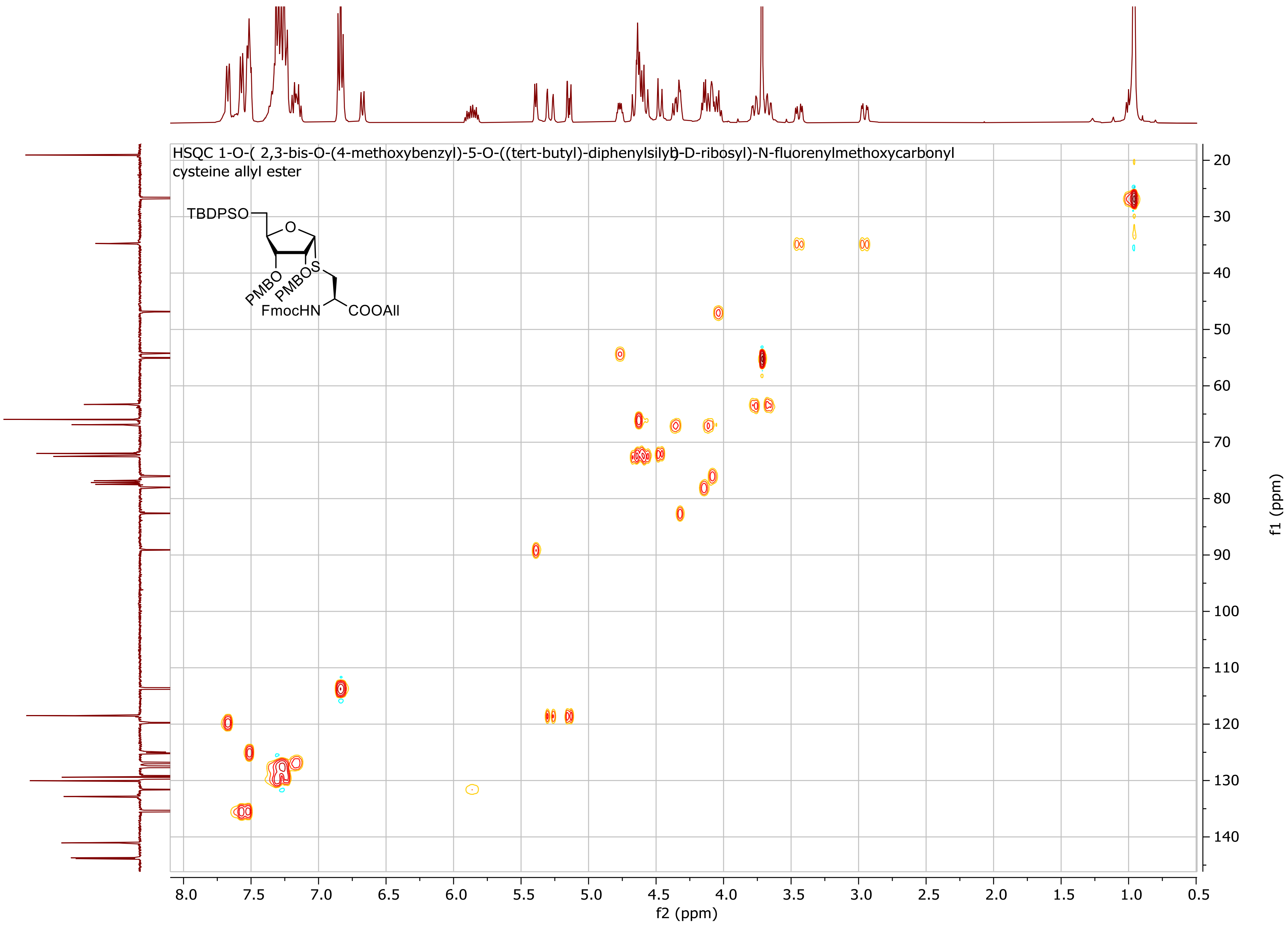
HSQC 1-O-(2,3-bis-O-(4-methoxybenzyl)-5-O-((tert-butyl)-diphenylsilyl)-D-ribosyl)-N-fluorenylmethoxycarbonyl
cysteine allyl ester
TBDPSO
O
PMBO
PMBO
S
FmocHN
COOAll
f2 (ppm)
f1 (ppm)

H-NMR 1-O-(2,3-bis-O-(4-methoxybenzyl)-5-O-((tert-butyl)-diphenylsilyl)-D-ribosyl)-N-fluorenylmethoxycarbonyl serine

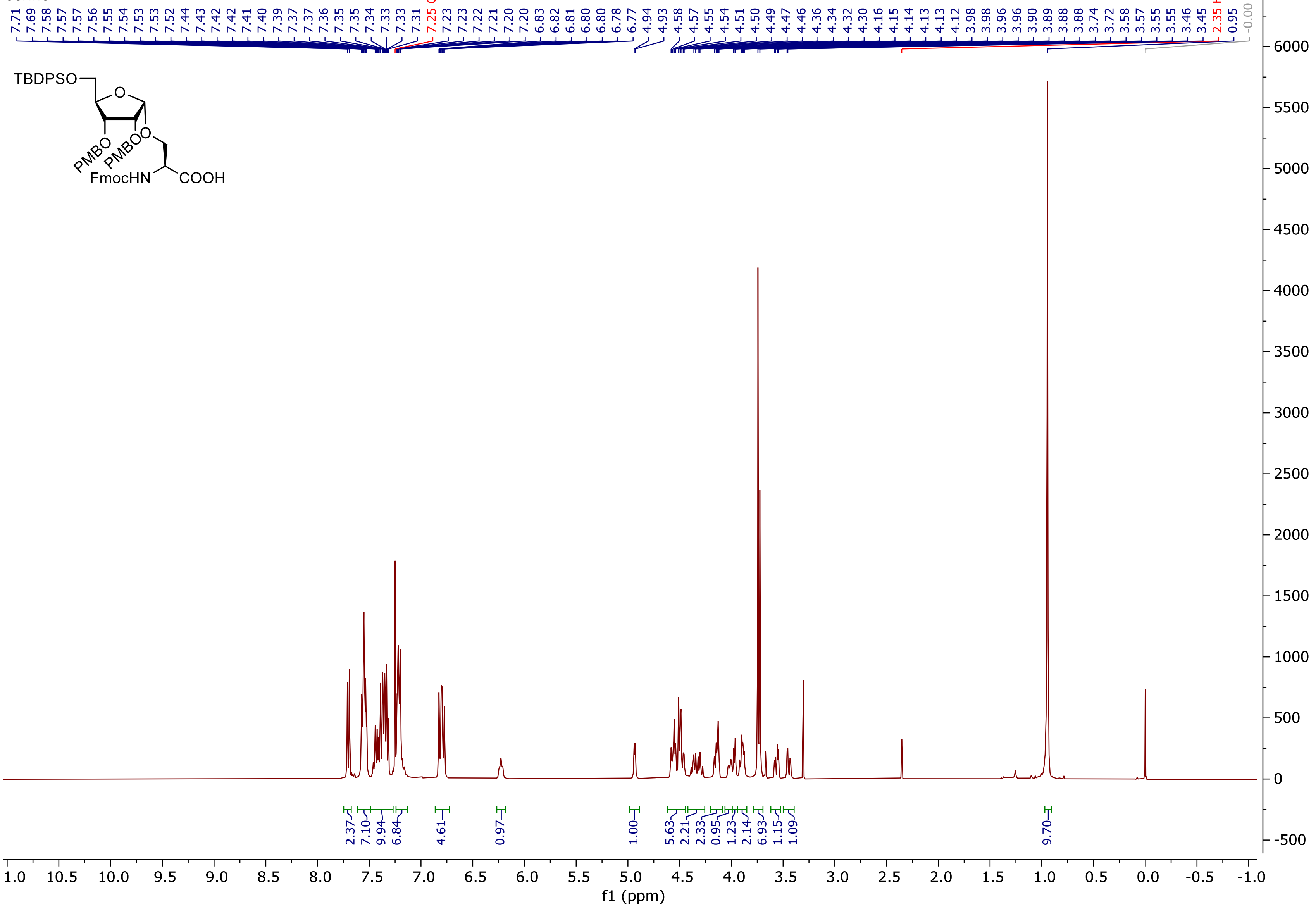

C-NMR 1-O-(2,3-bis-O-(4-methoxybenzyl)-5-O-((tert-butyl)-diphenylsilyl)-D-ribosyl)-N-fluorenylmethoxycarbonyl — serine —

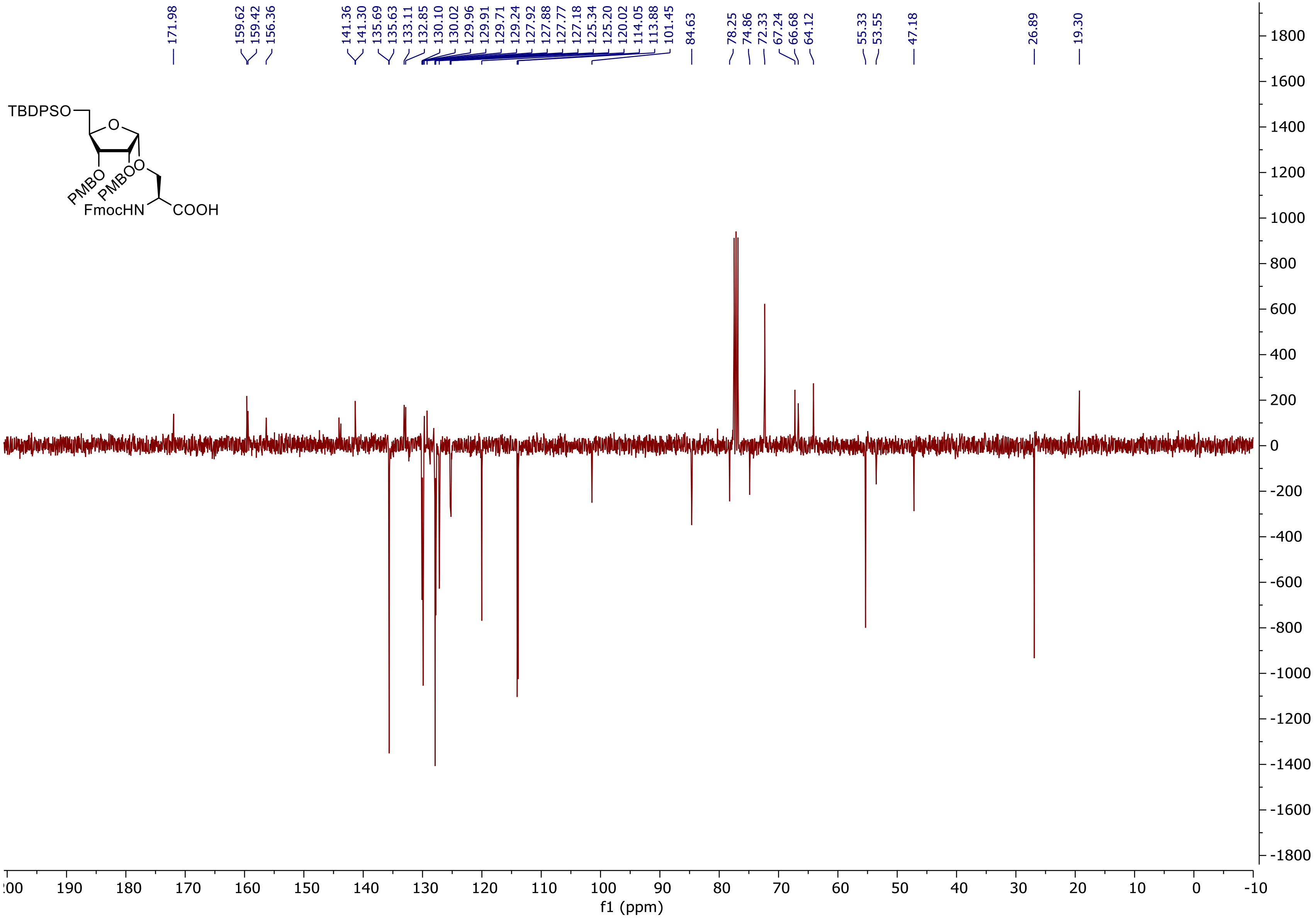

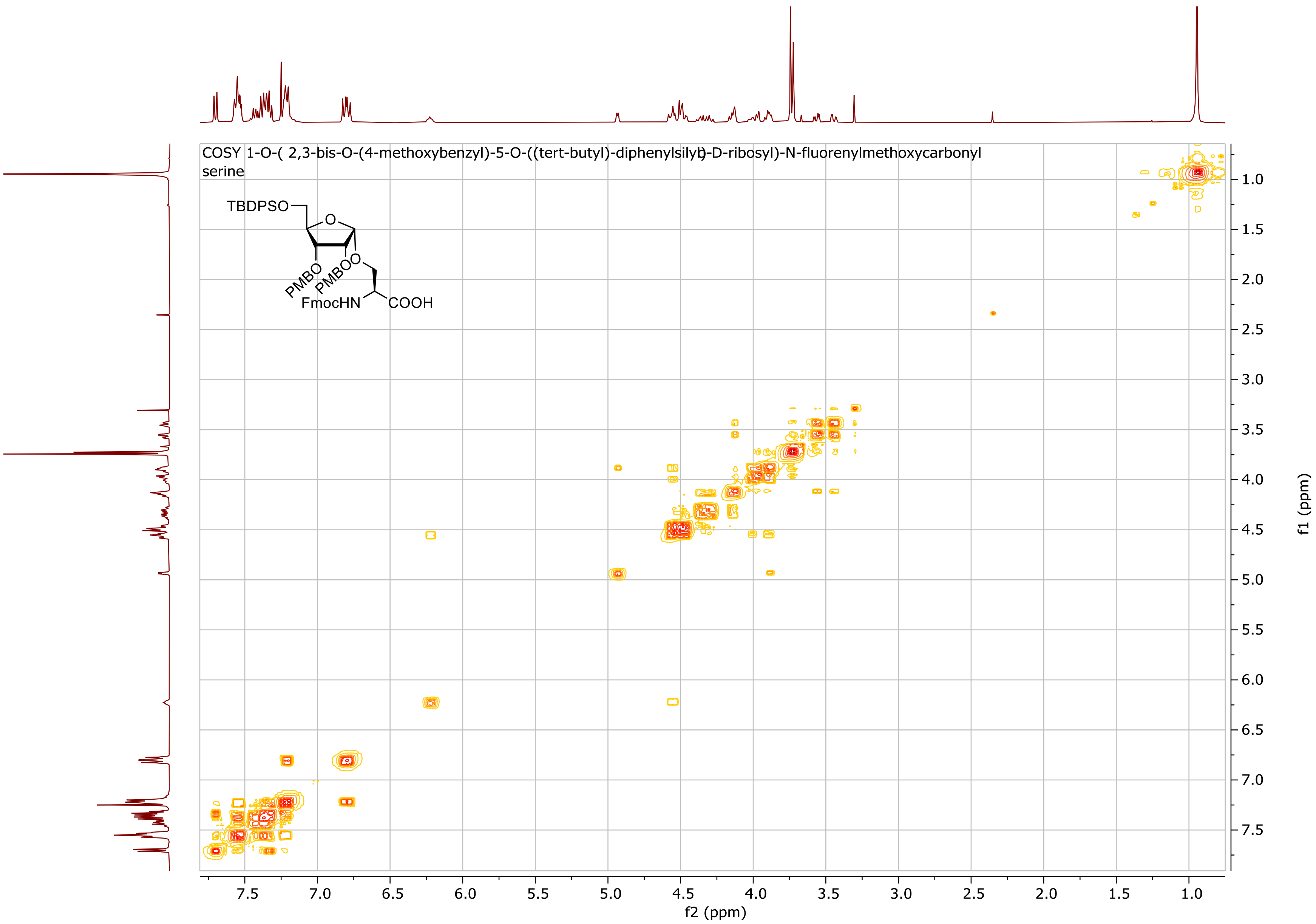
COSY 1-O-(2,3-bis-O-(4-methoxybenzyl)-5-O-((tert-butyl)-diphenylsilyl)-D-ribosyl)-N-fluorenylmethoxycarbonyl serine
TBDPSO
O
PMBO
PMBO
O
FmocHN
COOH
f2 (ppm)
f1 (ppm)

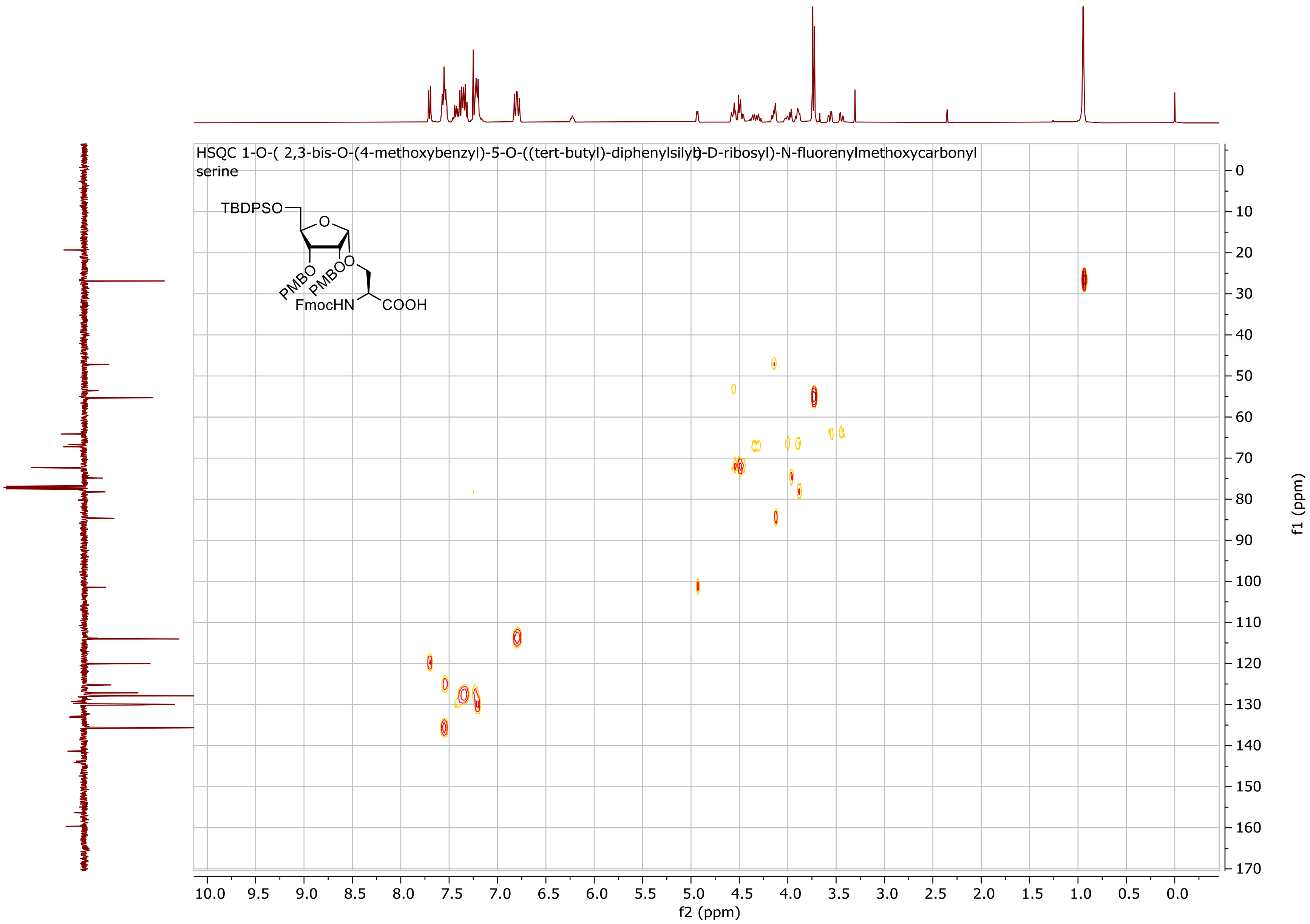
HSQC 1-O-(2,3-bis-O-(4-methoxybenzyl)-5-O-((tert-butyl)-diphenylsilyl)-D-ribosyl)-N-fluorenylmethoxycarbonyl serine
TBDPSO
O
PMBO
PMBO
O
FmocHN
COOH
f2 (ppm)
f1 (ppm)

H-NMR 1-O-(2,3-bis-O-(4-methoxybenzyl)-5-O-((tert-butyl)-diphenylsilyl)-D-ribosyl)-N-fluorenylmethoxycarbonyl — threonine —

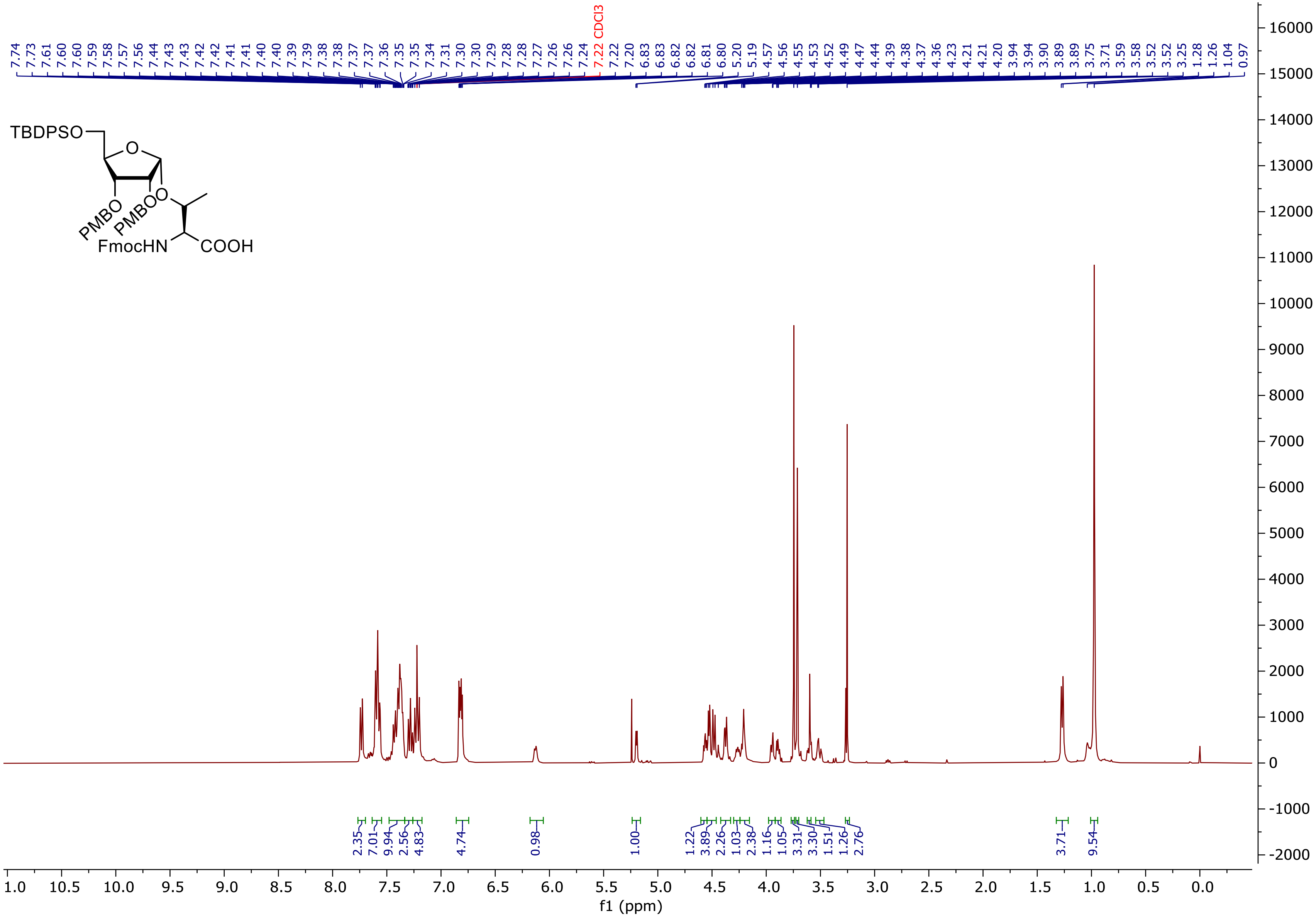

C-NMR 1-O-(2,3-bis-O-(4-methoxybenzyl)-5-O-((tert-butyl)-diphenylsilyl)-D-ribosyl)-N-fluorenylmethoxycarbonyl — threonine —

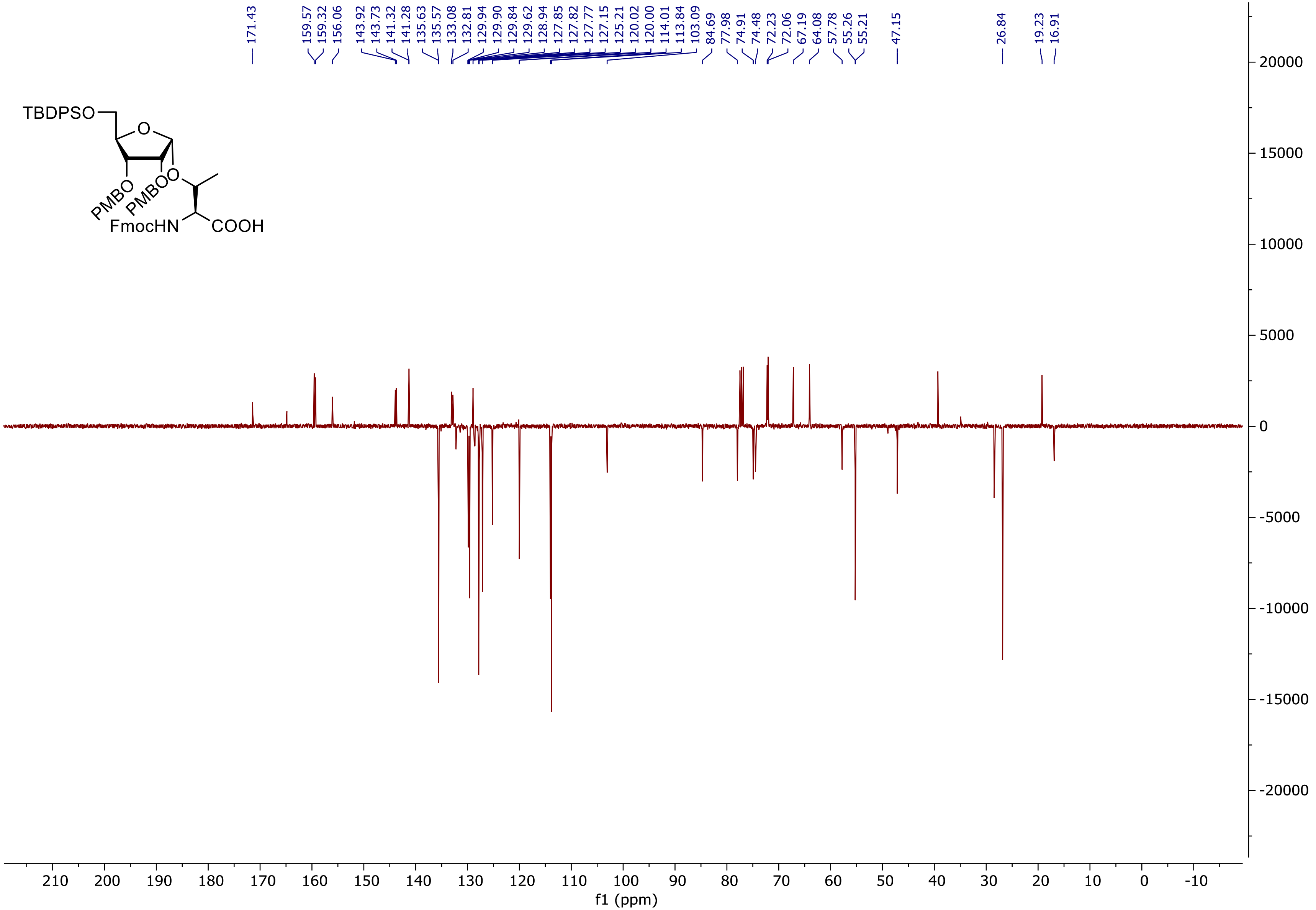

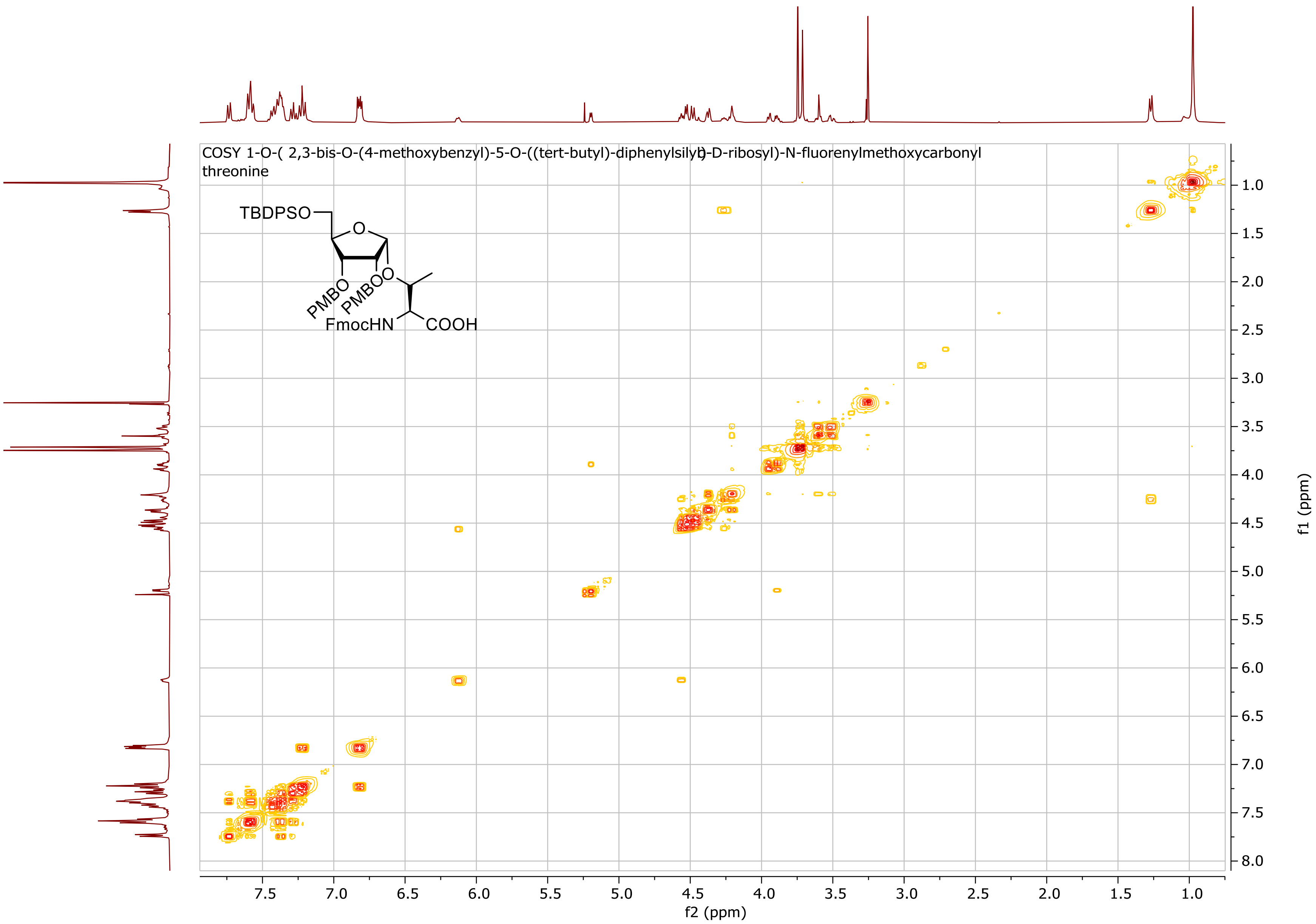
COSY 1-O-(2,3-bis-O-(4-methoxybenzyl)-5-O-((tert-butyl)-diphenylsilyl)-D-ribosyl)-N-fluorenylmethoxycarbonyl threonine
TBDPSO
O
PMBO
PMBO
O
FmocHN
COOH
f2 (ppm)
f1 (ppm)
7.5
7.0
6.5
6.0
5.5
5.0
4.5
4.0
3.5
3.0
2.5
2.0
1.5
1.0
8.0

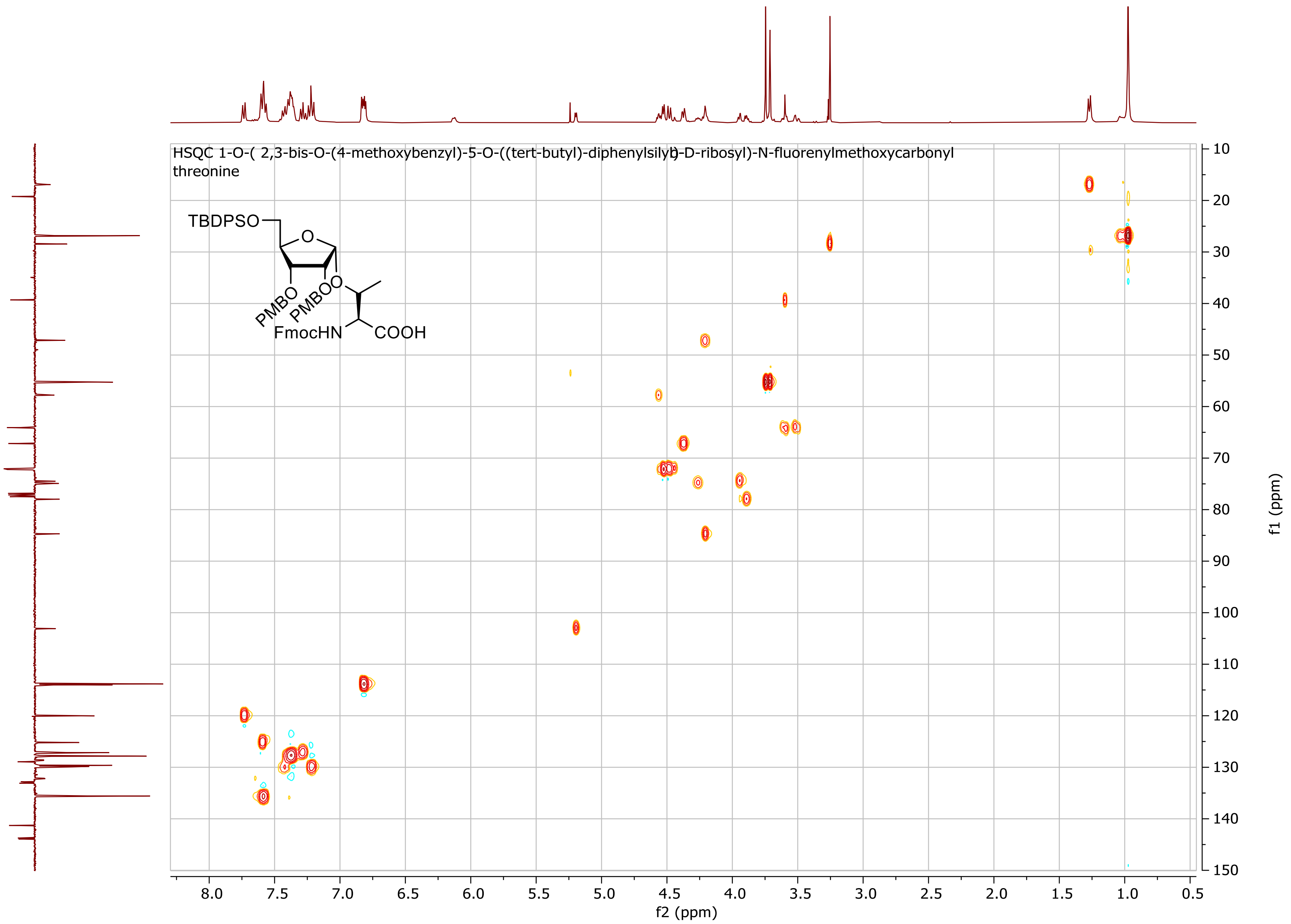
HSQC 1-O-(2,3-bis-O-(4-methoxybenzyl)-5-O-((tert-butyl)-diphenylsilyl)-D-ribosyl)-N-fluorenylmethoxycarbonyl threonine
TBDPSO
O
PMBO
PMBO
O
FmocHN
COOH
f2 (ppm)
f1 (ppm)

H-NMR 1-O-(2,3-bis-O-(4-methoxybenzyl)-5-O-((tert-butyl)-diphenylsilyl)-D-ribosyl)-N-fluorenylmethoxycarbonyl — cysteine —

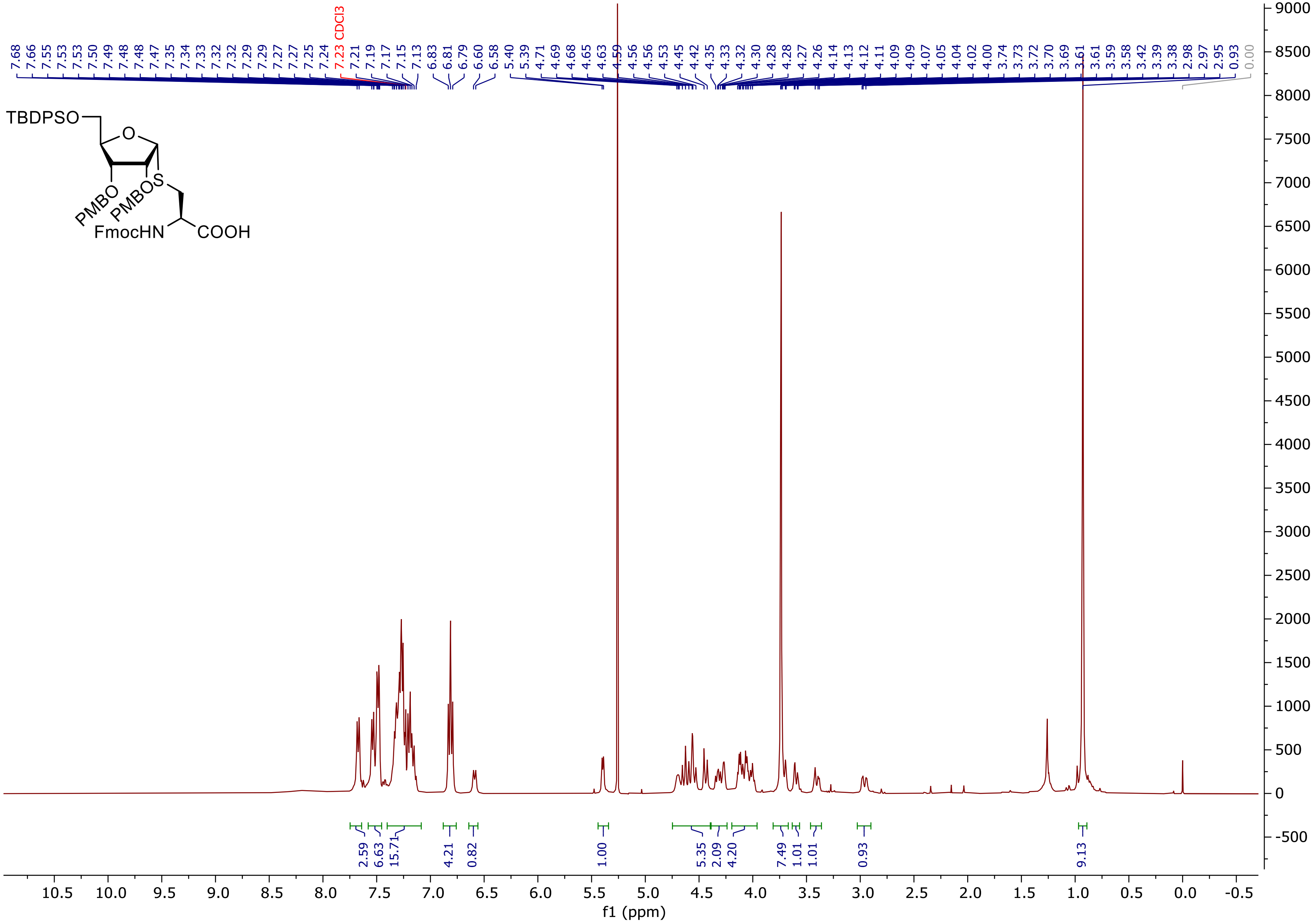

C-NMR 1-O-(2,3-bis-O-(4-methoxybenzyl)-5-O-((tert-butyl)-diphenylsilyl)-D-ribosyl)-N-fluorenylmethoxycarbonyl — cysteine —

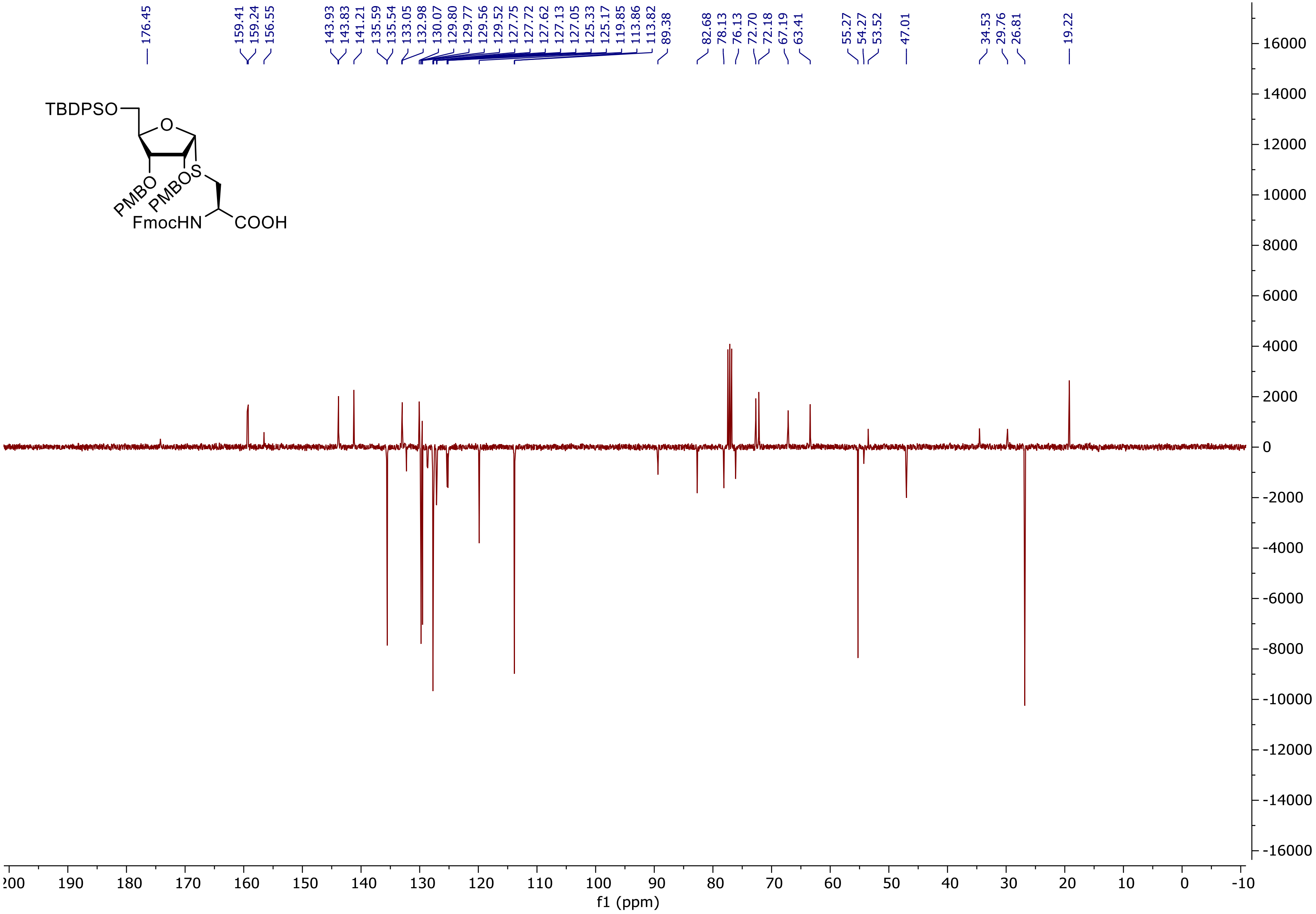

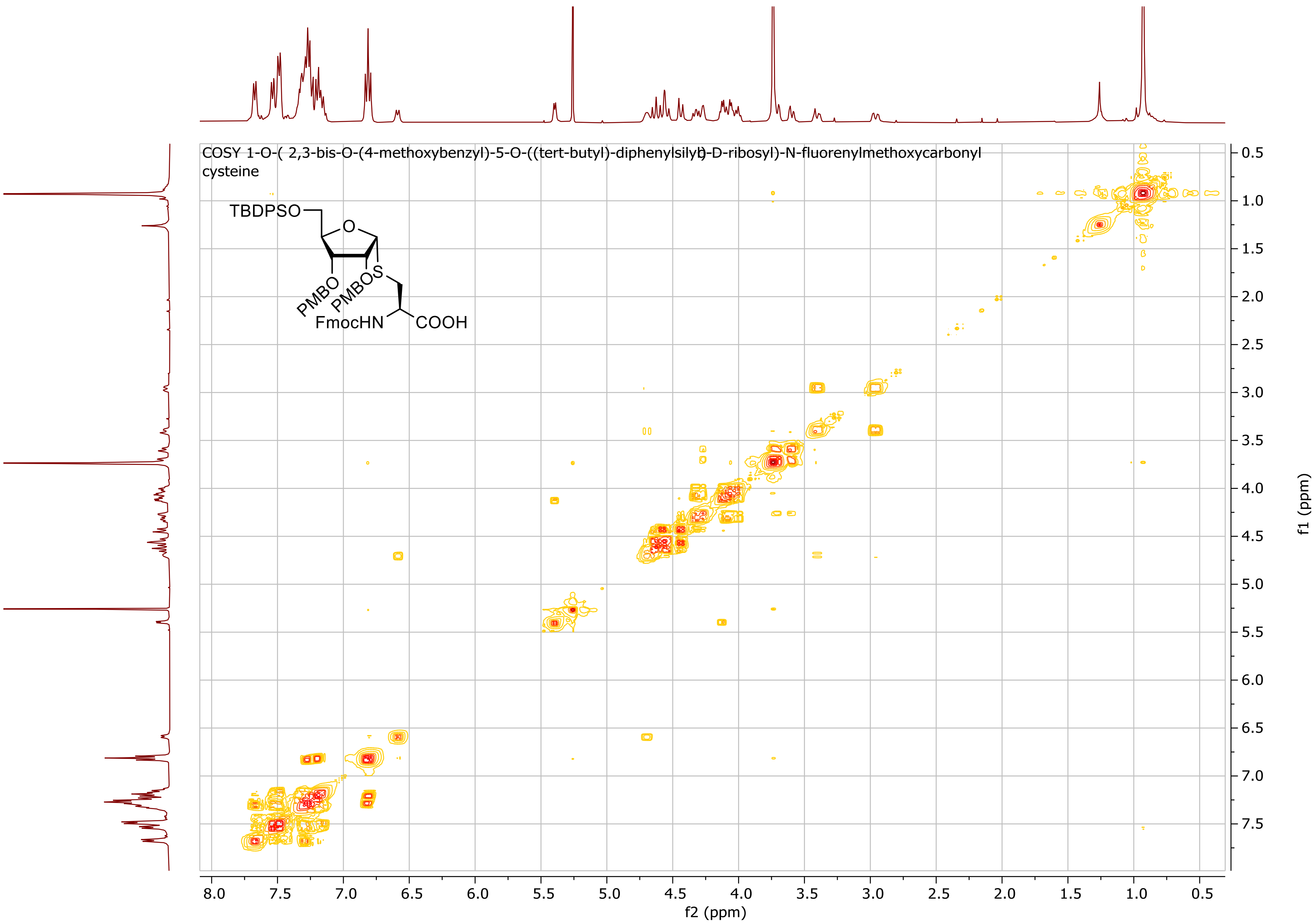
COSY 1-O-(2,3-bis-O-(4-methoxybenzyl)-5-O-((tert-butyl)-diphenylsilyl)-D-ribosyl)-N-fluorenylmethoxycarbonyl cysteine
TBDPSO
O
PMBO
PMBO
S
FmocHN
COOH
f2 (ppm)
f1 (ppm)

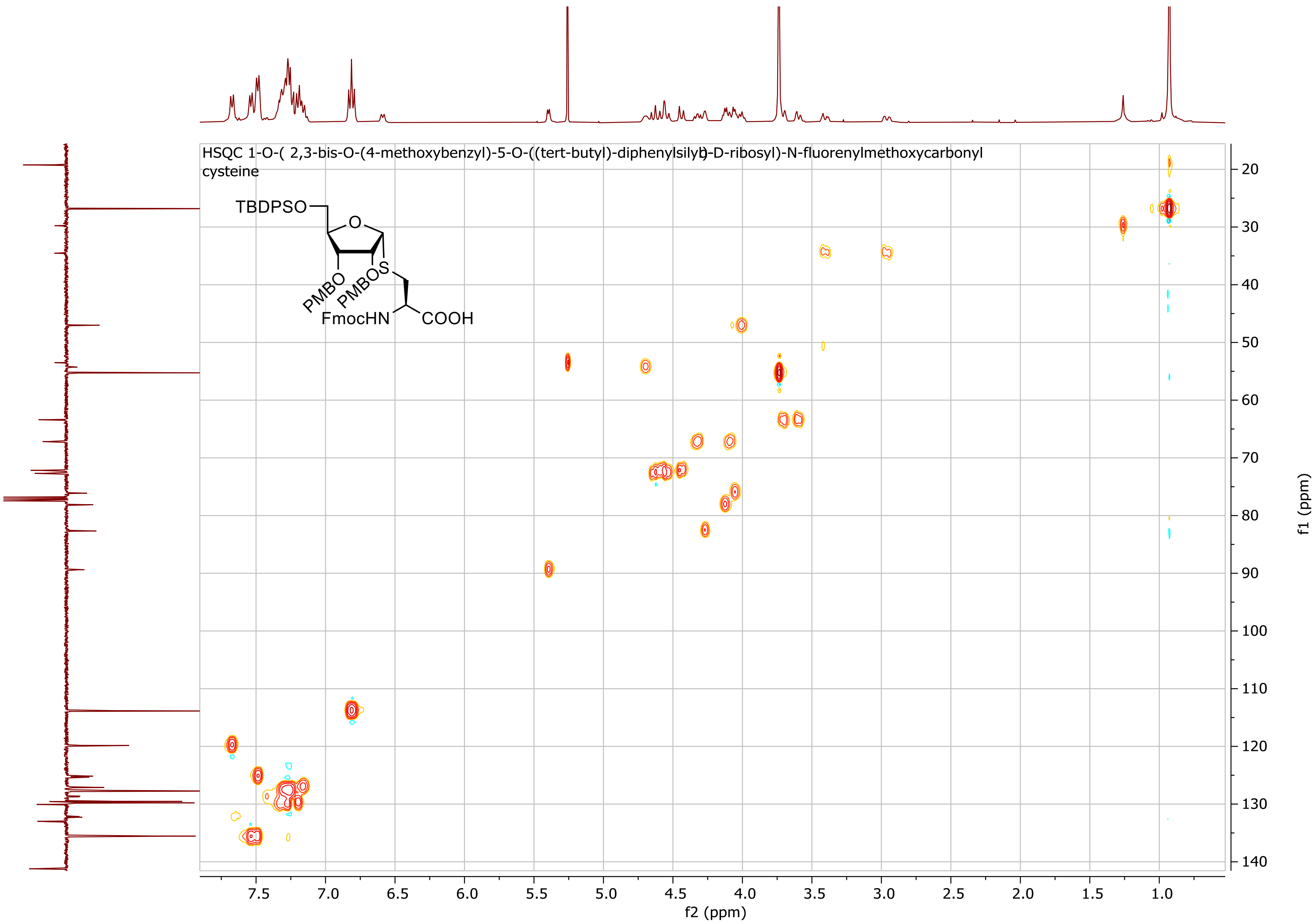
HSQC 1-O-(2,3-bis-O-(4-methoxybenzyl)-5-O-((tert-butyl)-diphenylsilyl)-D-ribosyl)-N-fluorenylmethoxycarbonyl cysteine
TBDPSO
O
PMBO
PMBO
S
FmocHN
COOH
f2 (ppm)
f1 (ppm)

H-NMR 5′-O-(N6-tert-butyloxycarbonyl-2′,3′-di-O-tert-butyldimethylsilyladenosine)-2-cyanoethyl-N-N-diisopropylphosphoramidite

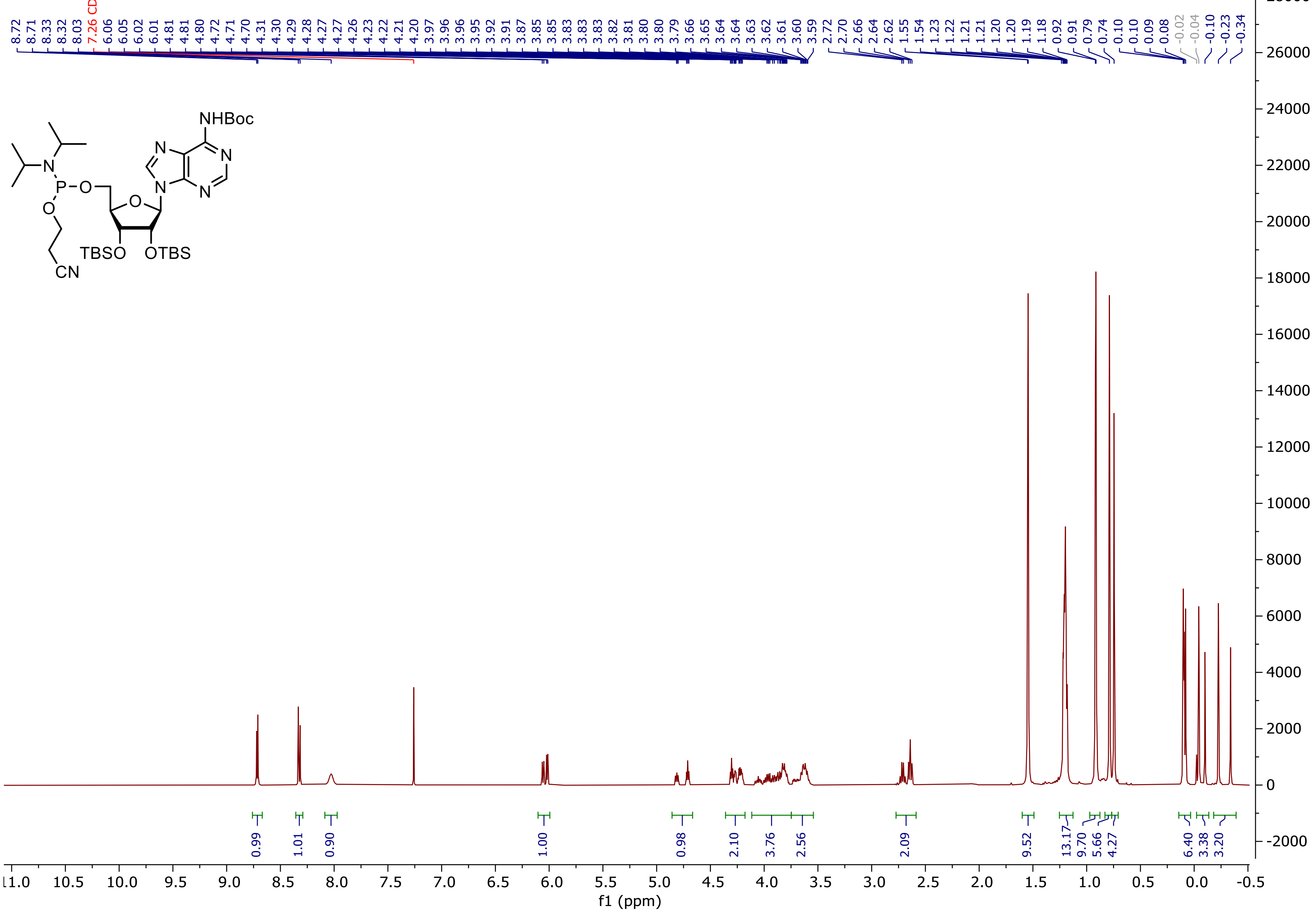

C-NMR 5’-O-(N6-tert-butyloxycarbonyl-2’,3’-di-O-tert-butyldimethylsilyladenosine)-2-cyanoethyl-N-N-diisopropylphosphoramidite

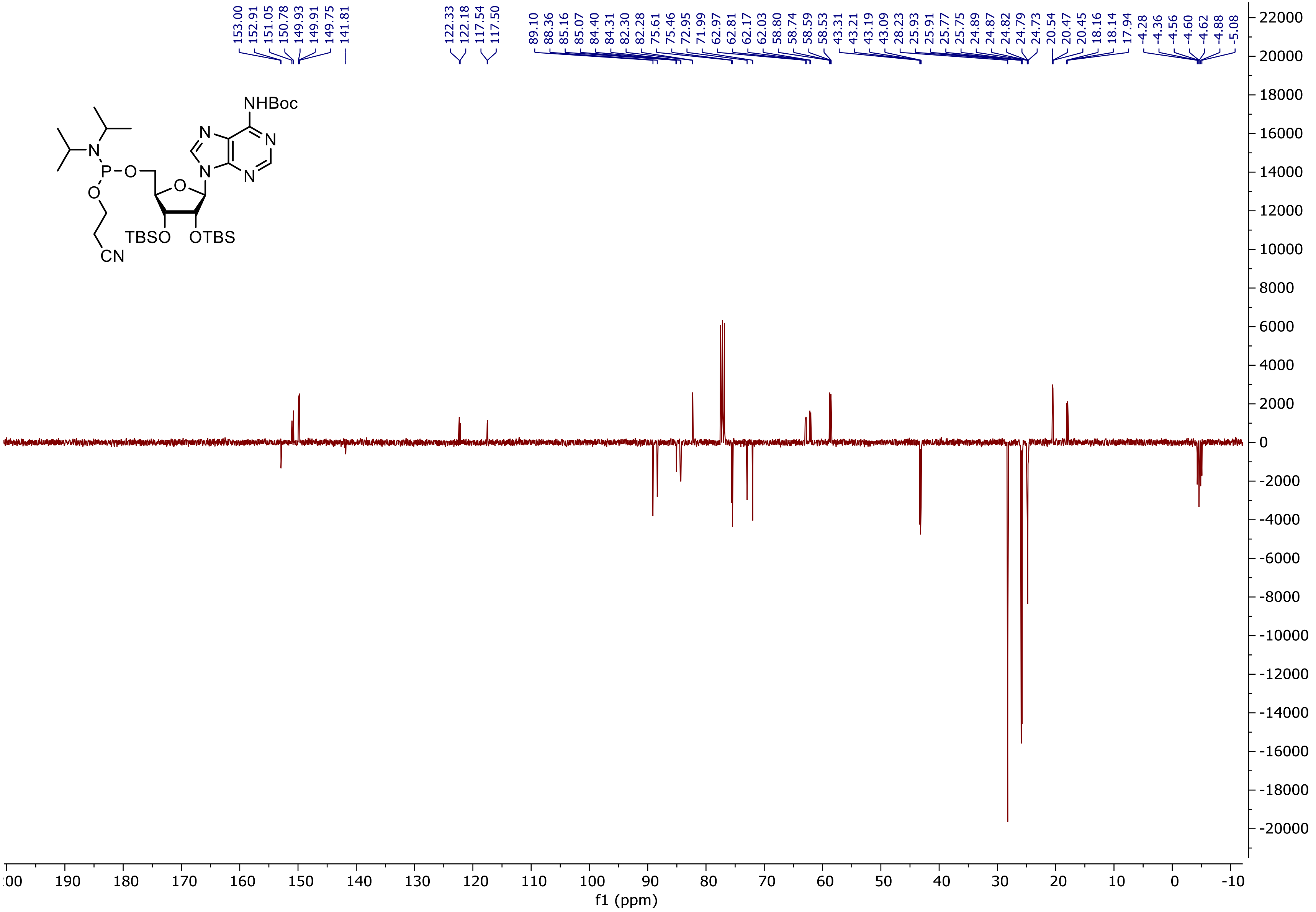

P-NMR 5'-O-(N6-tert-butyloxycarbonyl-2',3'-di-O-tert-butyldimethylsilyladenosine)-2-cyanoethyl-N-N-diisopropylphosphoramidite

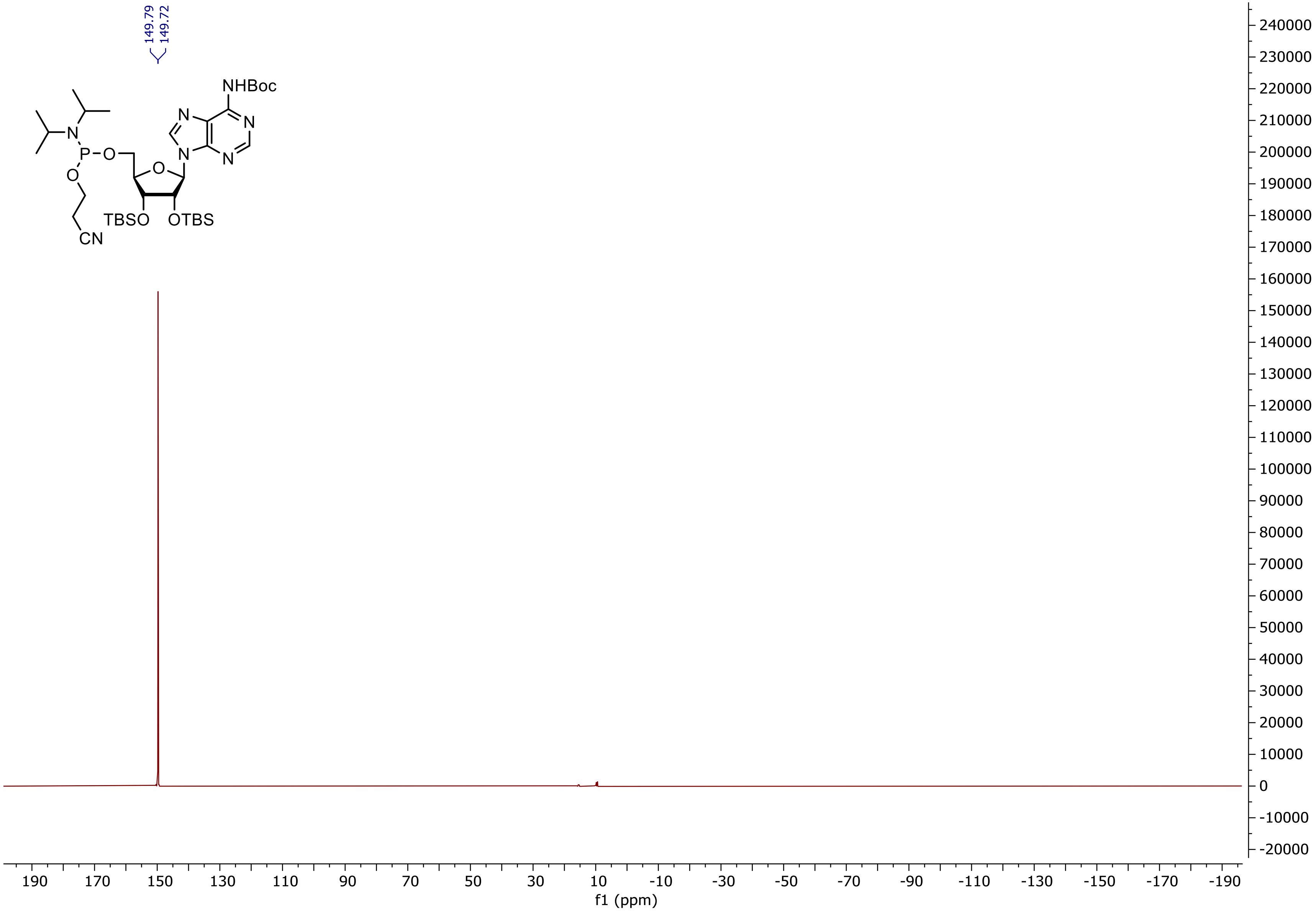

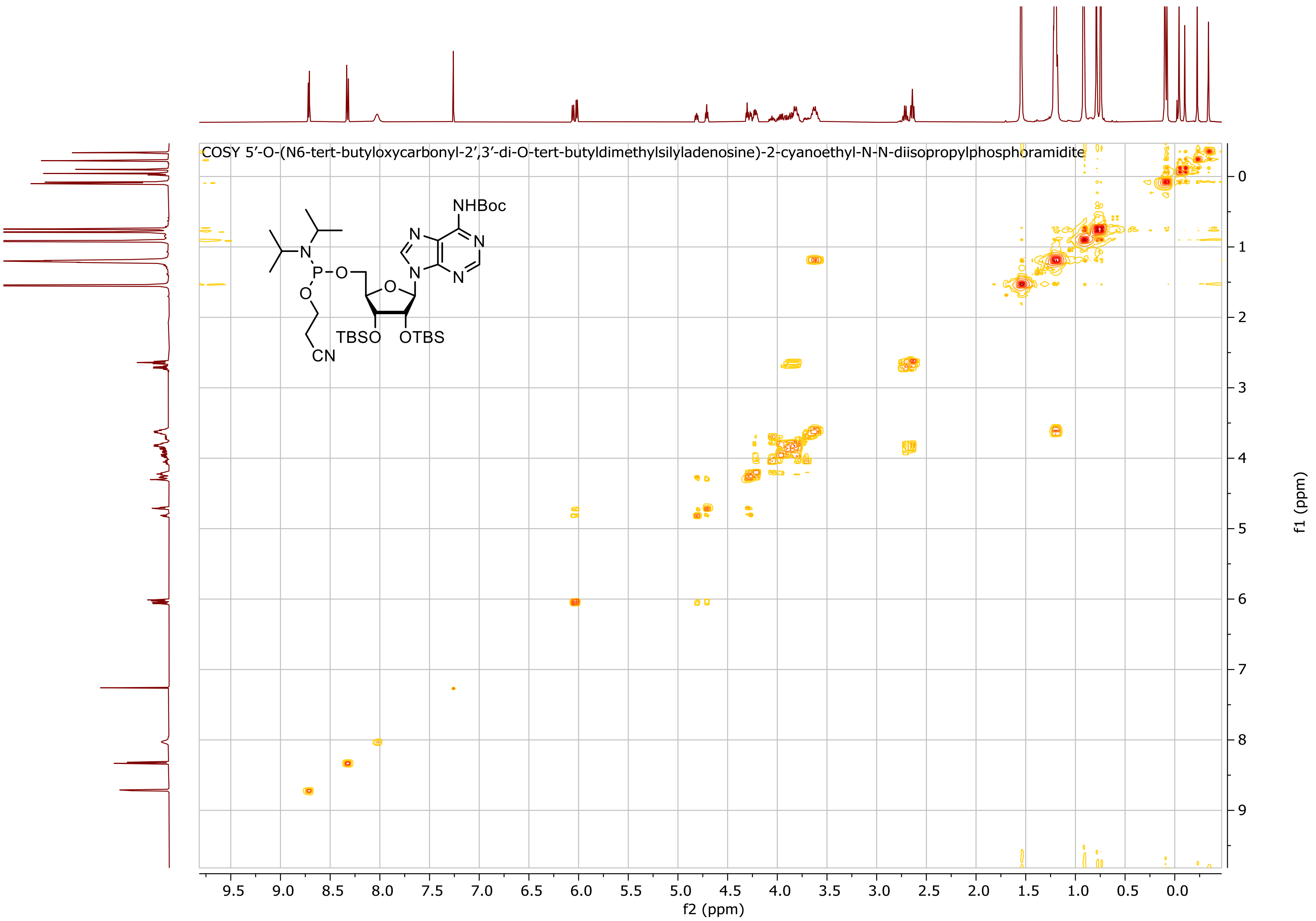
COSY 5′-O-(N6-tert-butyloxycarbonyl-2′,3′-di-O-tert-butyldimethylsilyladenosine)-2-cyanoethyl-N-N-diisopropylphosphoramidite
NHBoc
N
P
O
TBSO
OTBS
CN
f2 (ppm)
f1 (ppm)

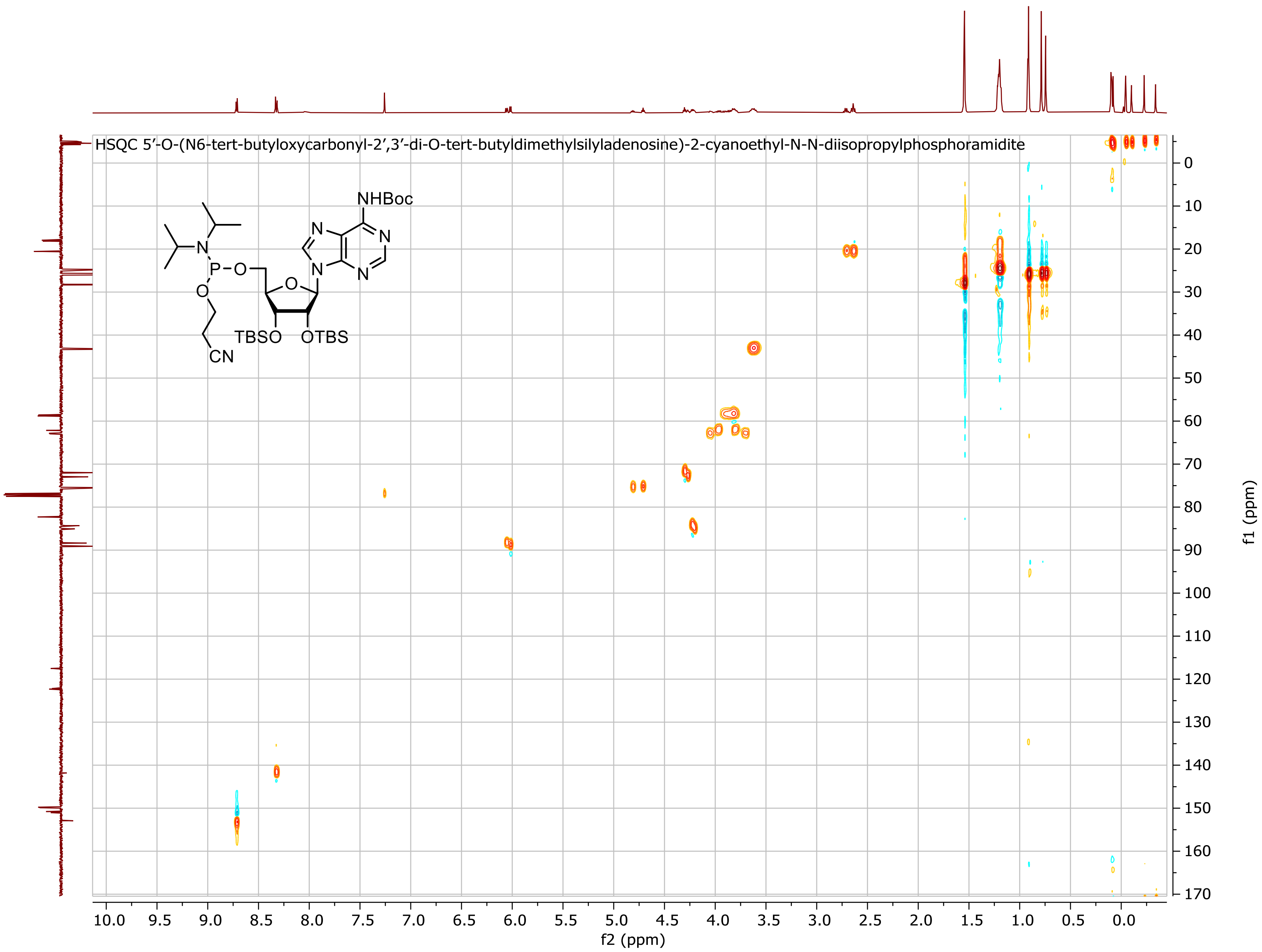
HSQC 5′-O-(N6-tert-butyloxycarbonyl-2′,3′-di-O-tert-butyldimethylsilyladenosine)-2-cyanoethyl-N-N-diisopropylphosphoramidite
NHBoc
TBSO
OTBS
CN
f2 (ppm)
f1 (ppm)

H-NMR Ac-PAKS(ADPr)APAPKKG-OH

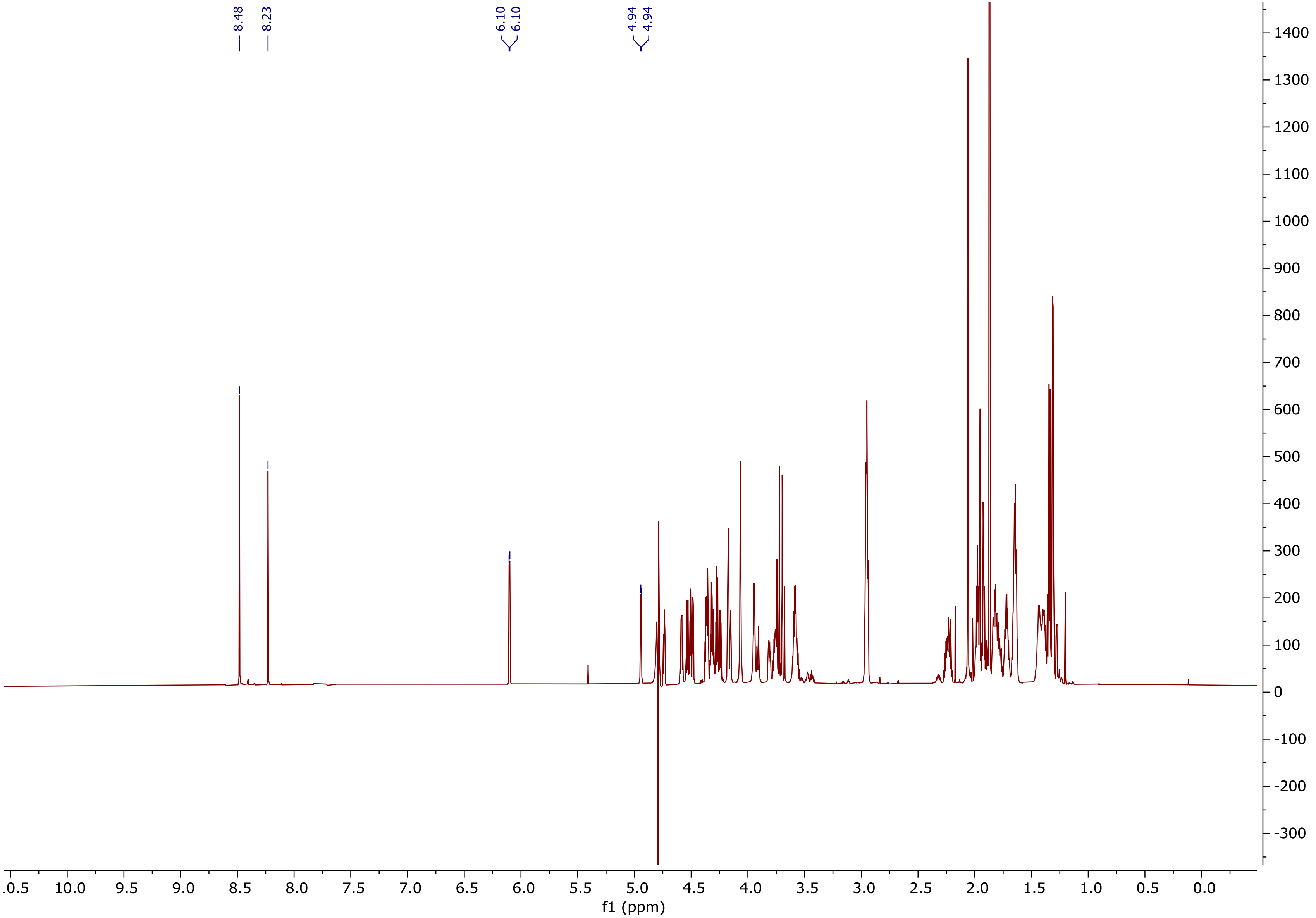

P-NMR PAKS(ADPr)APAPKKG-OH + EDTA

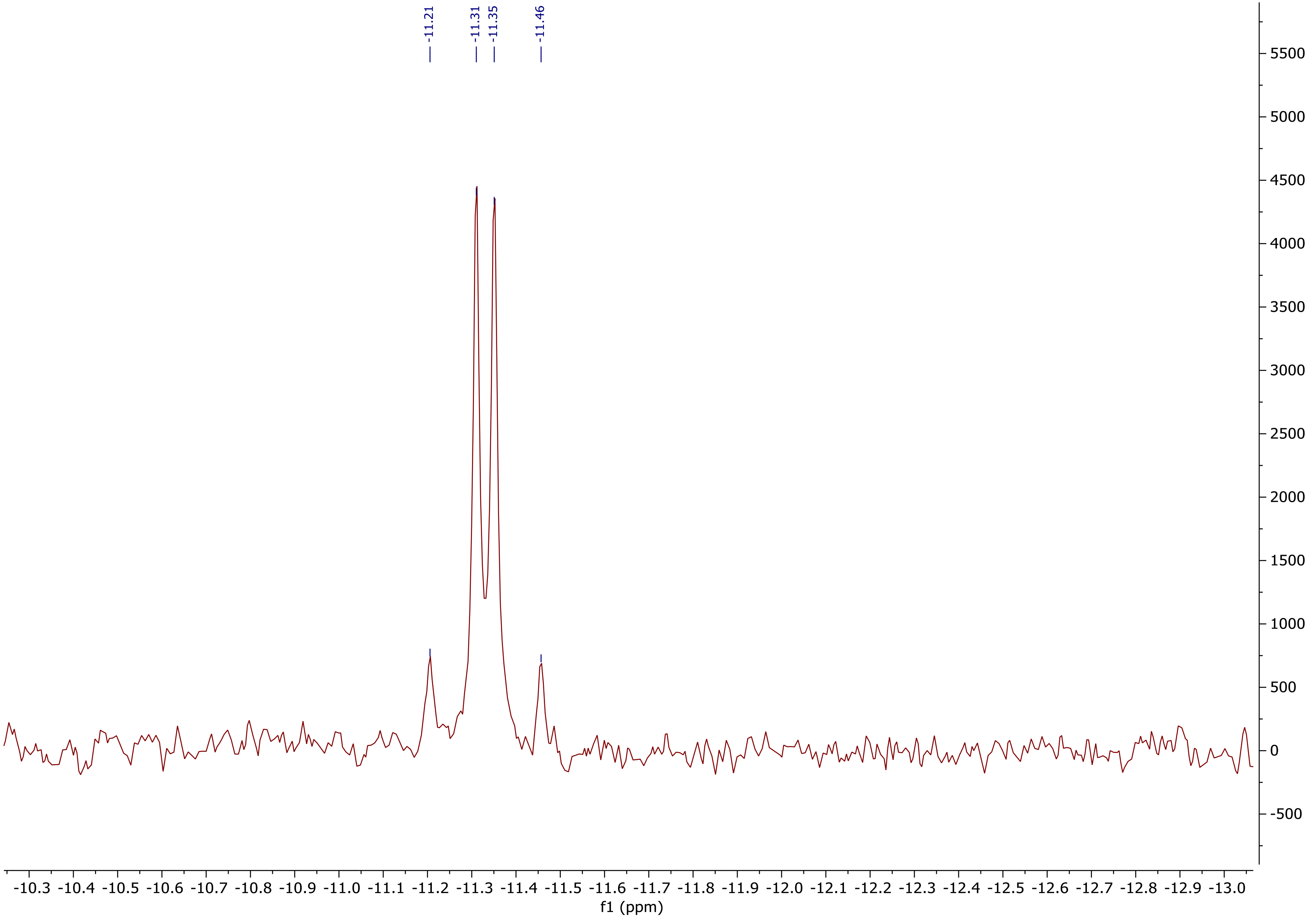

H-NMR Ac-GKS(ADPr)GAALSKKG-OH

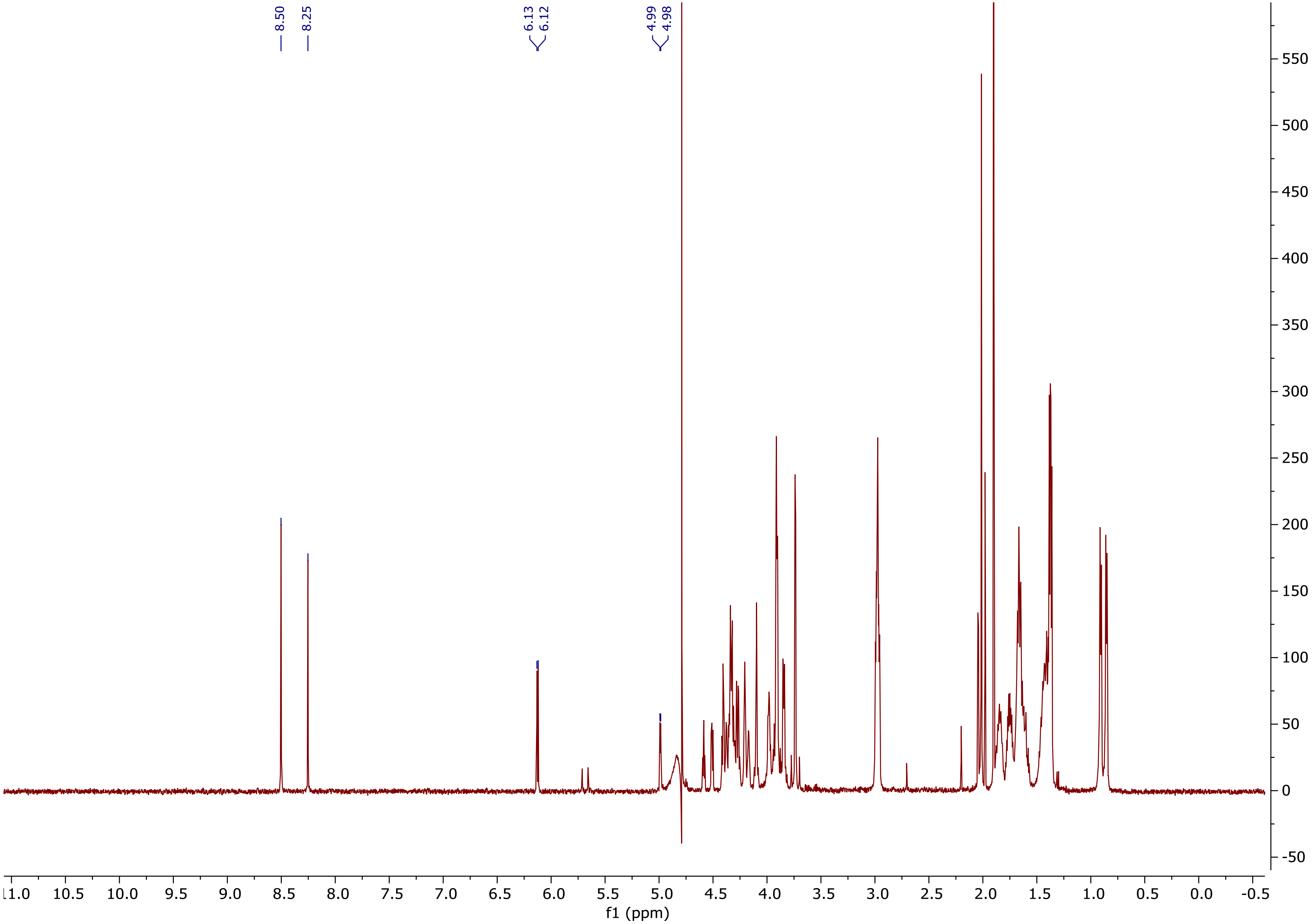

P-NMR Ac-GKS(ADPr)GAALSKKG-OH + EDTA

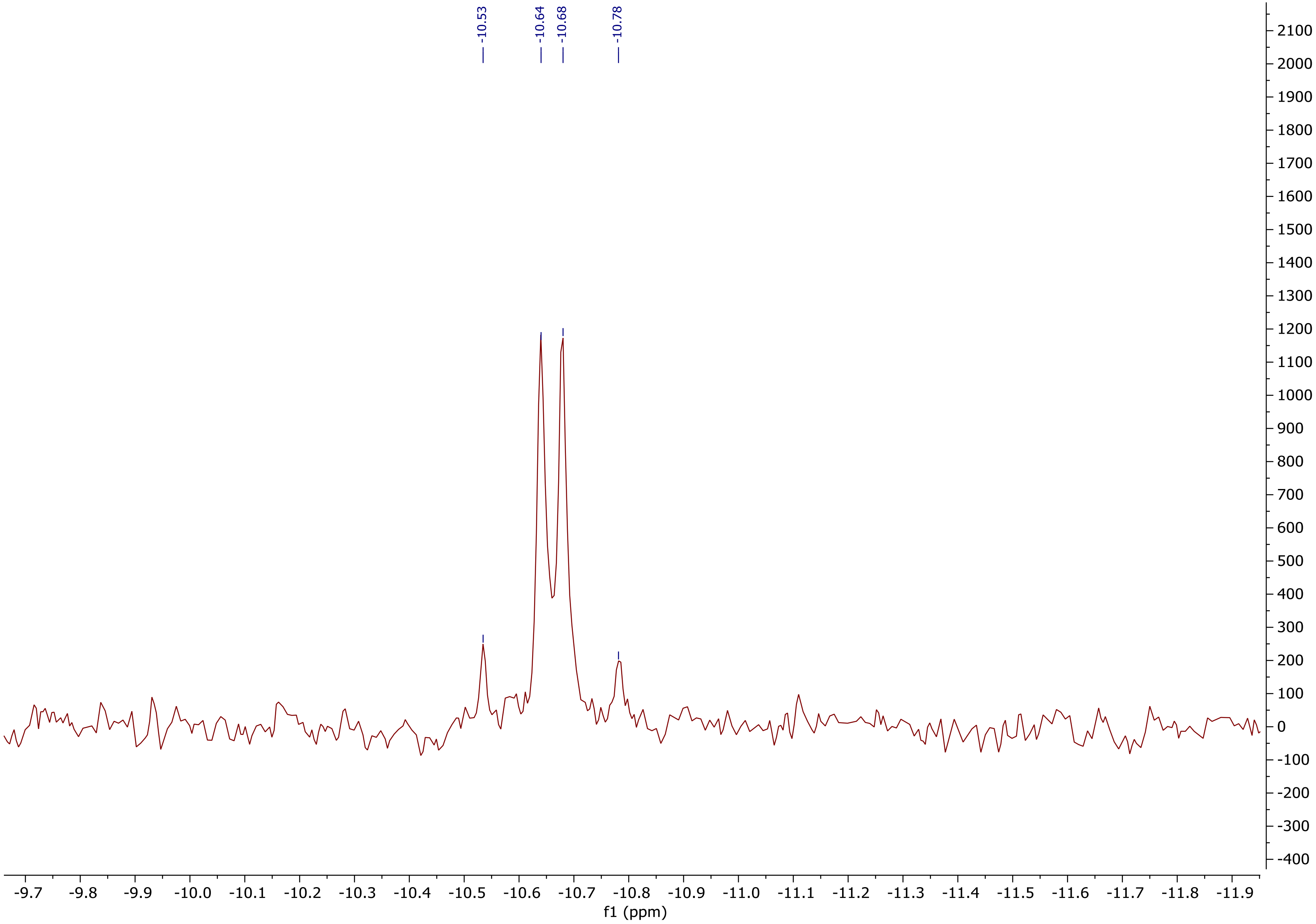

H-NMR Ac-GKS(ADPr)SGPTSLFAVTVAPPGARG-OH

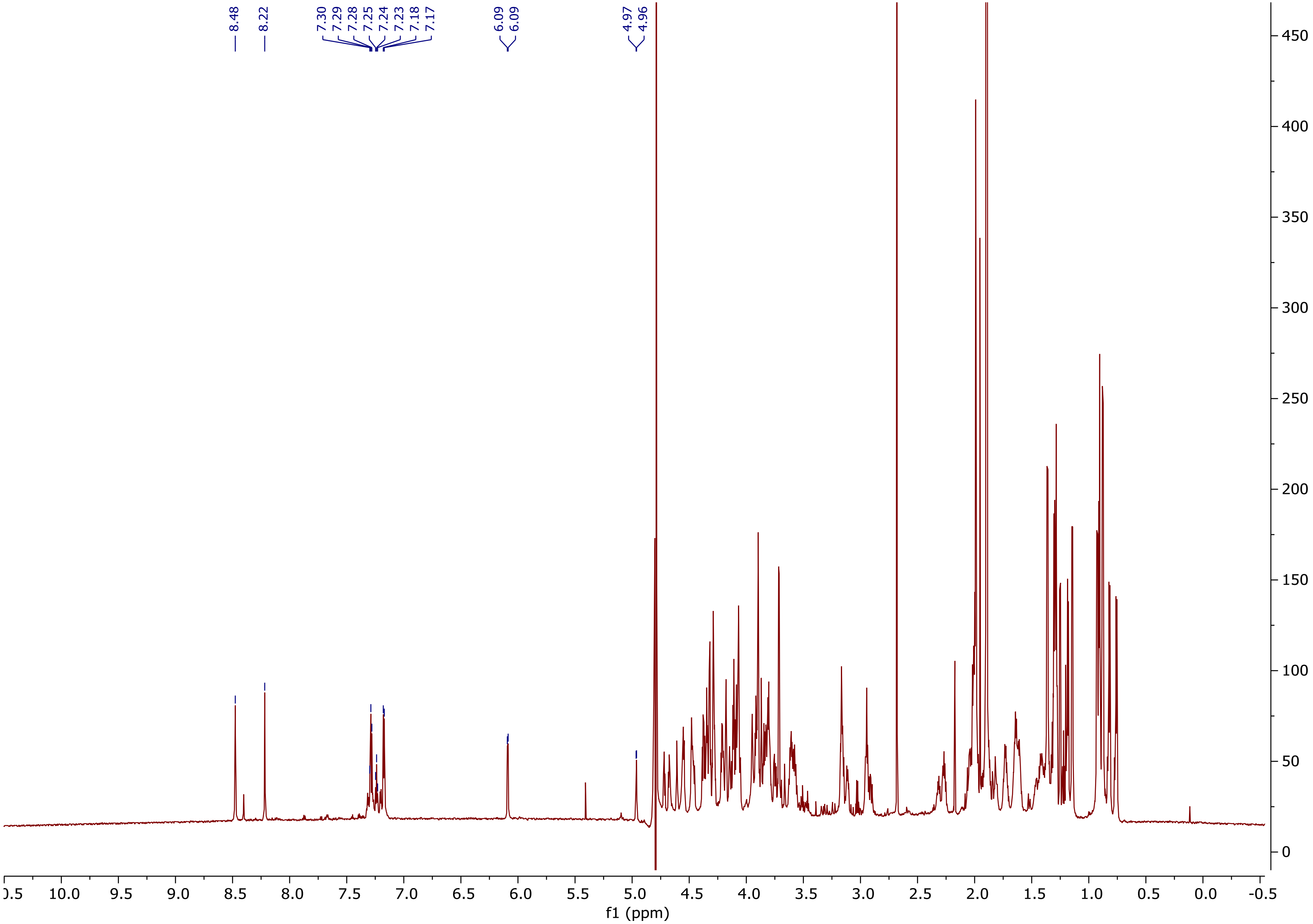

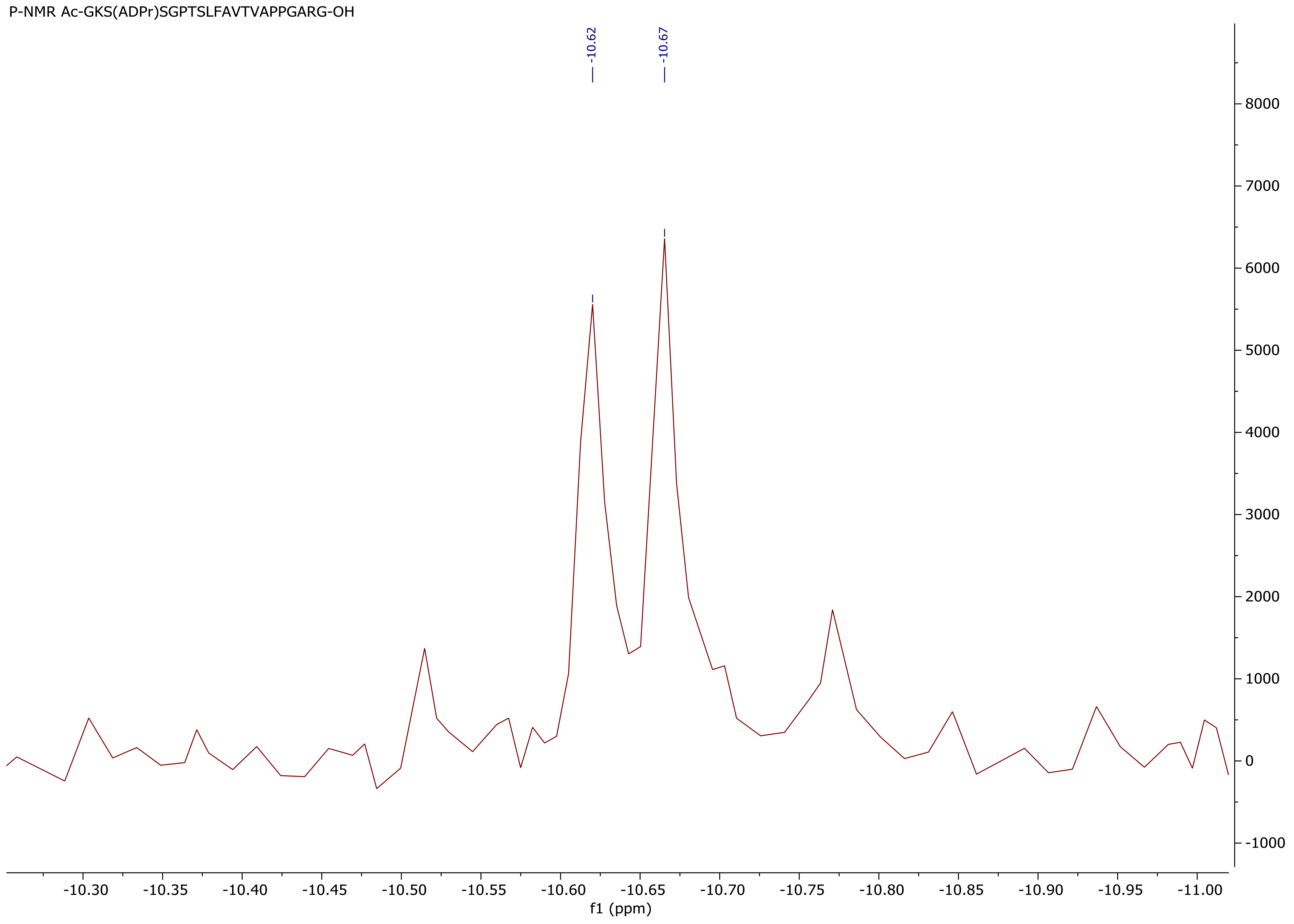

P-NMR Ac-GKS(ADPr)SGPTSLFAVTVAPPGARG-OH
-10.62
-10.67
8000
7000
6000
5000
4000
3000
2000
1000
0
-1000
-10.30
-10.35
-10.40
-10.45
-10.50
-10.55
-10.60
-10.65
-10.70
-10.75
-10.80
-10.85
-10.90
-10.95
-11.00
f1 (ppm)

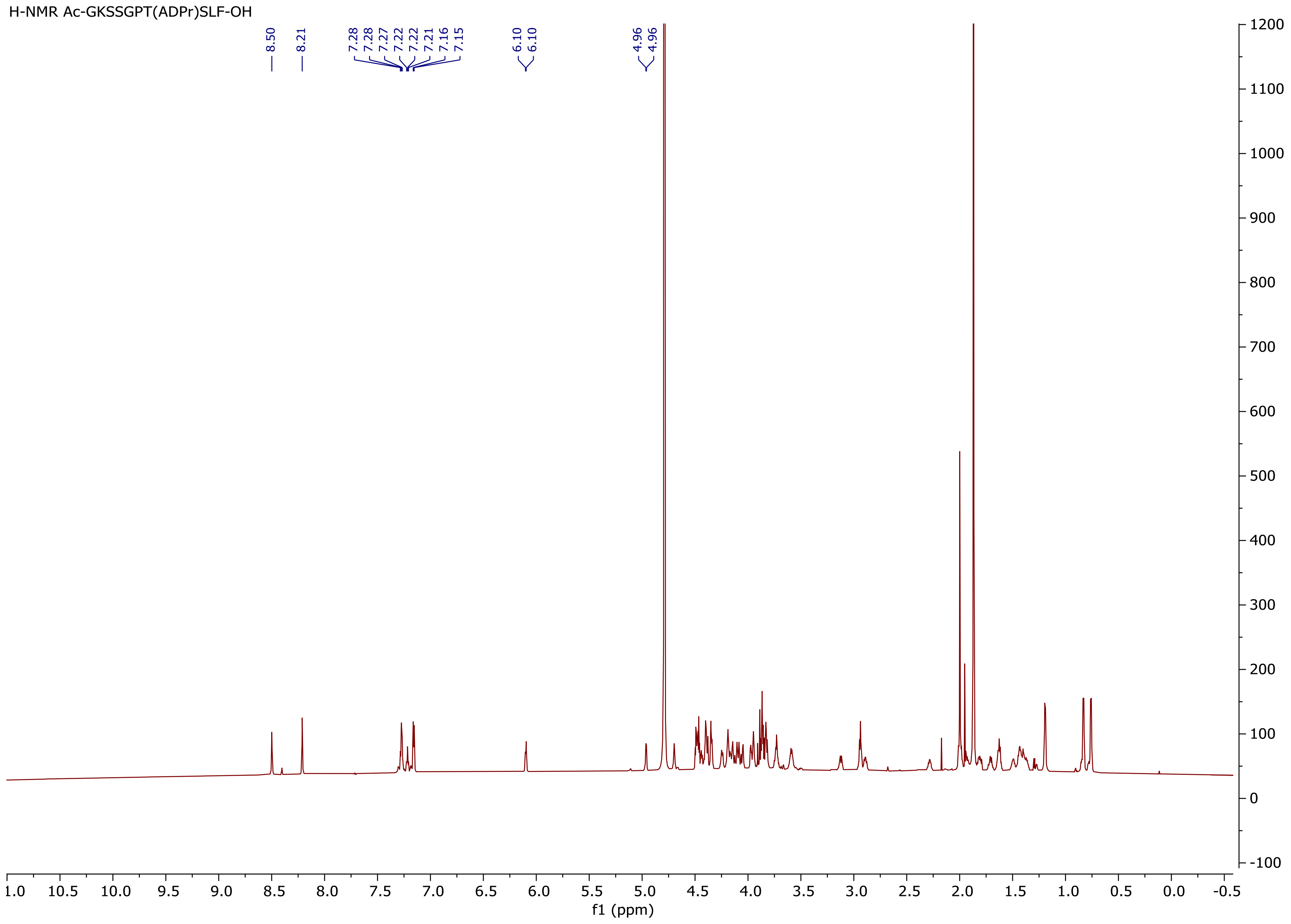
H-NMR Ac-GKSSGPT(ADPr)SLF-OH
8.50
8.21
7.28
7.28
7.27
7.22
7.22
7.21
7.16
7.15
6.10
6.10
4.96
4.96
f1 (ppm)

P-NMR Ac-GKSSGPT(ADPr)-SLF-OH

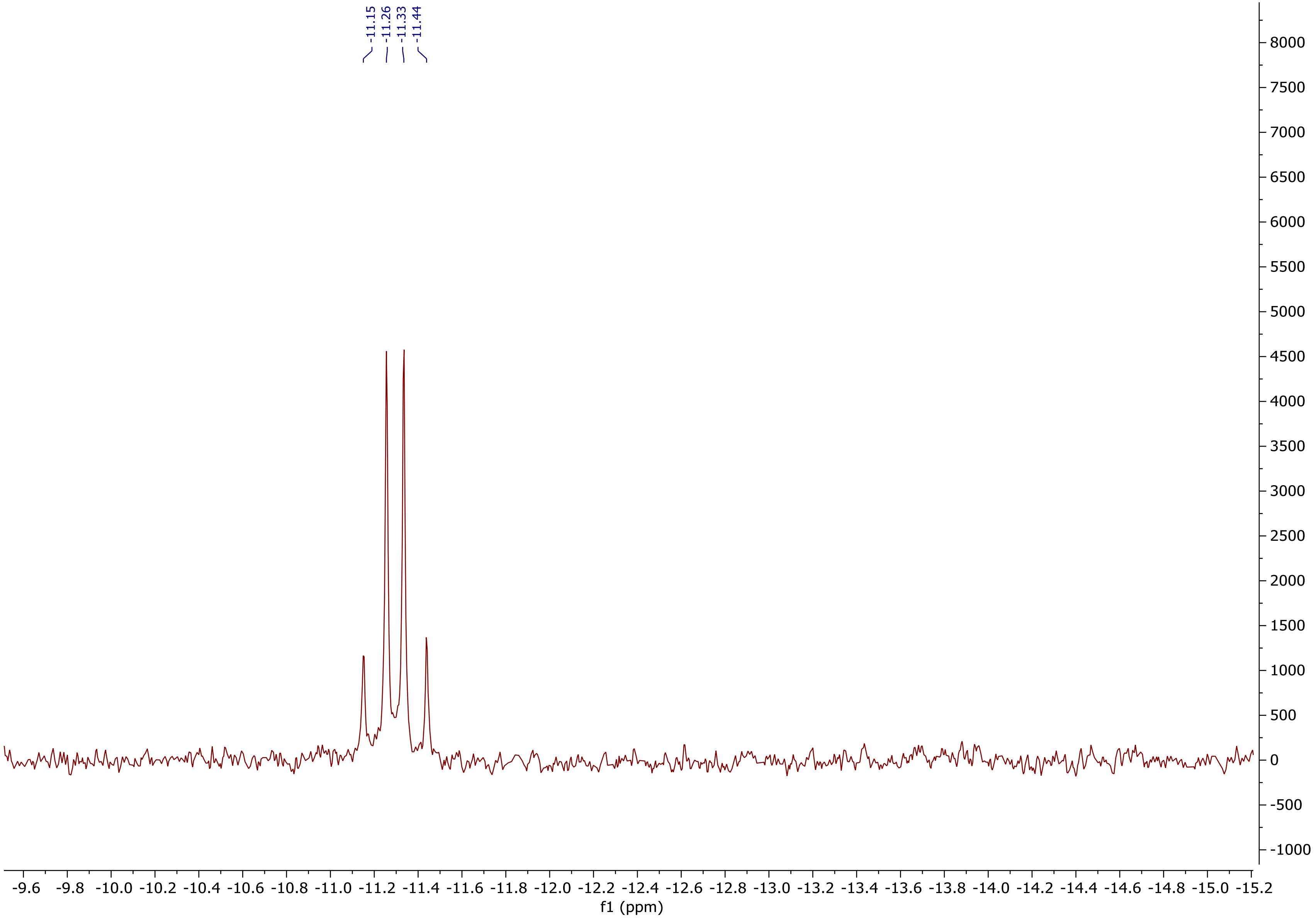

H-NMR Ac-KEST(ADPr)LHLVLRL-OH

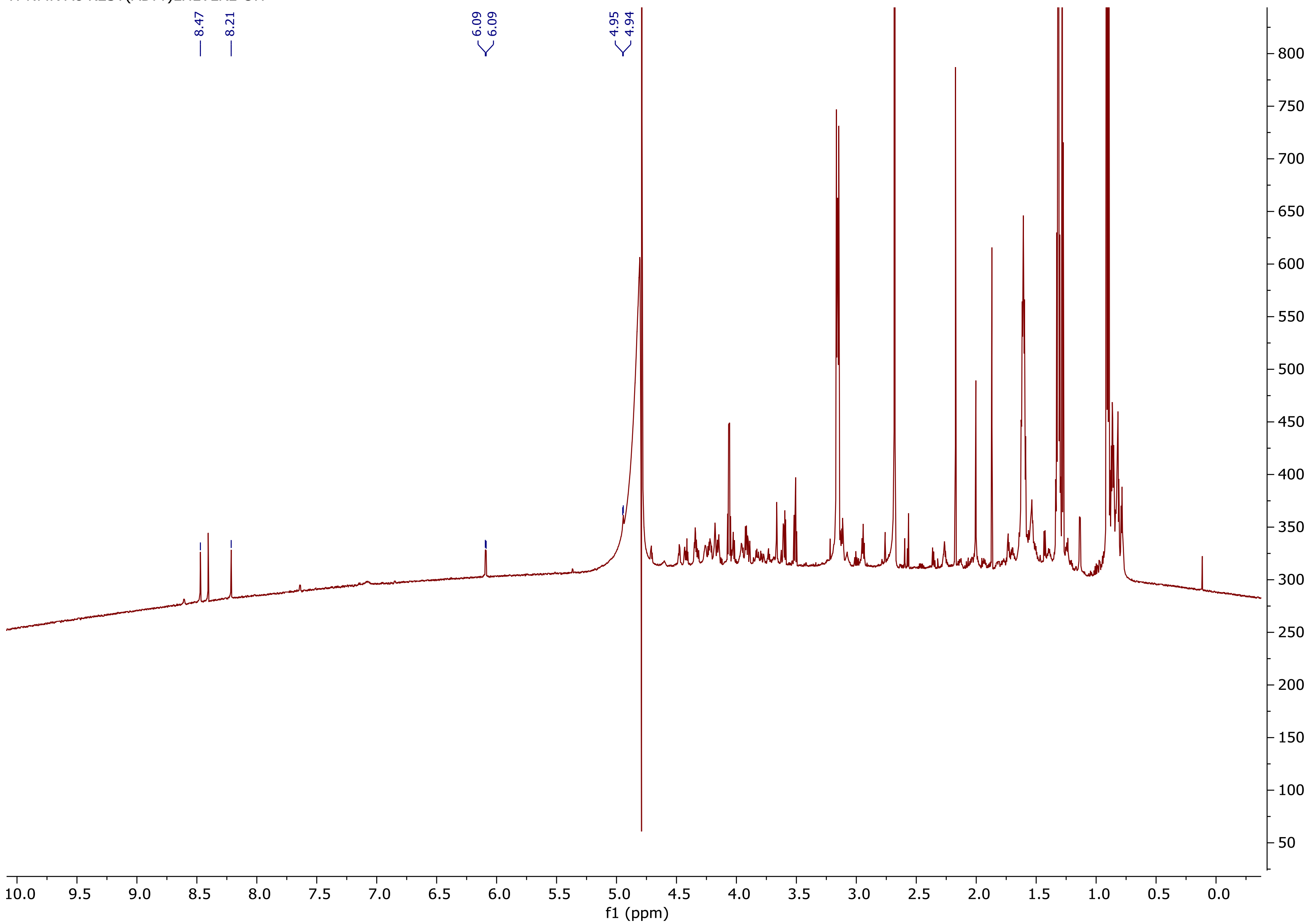

H-NMR Ac-PAKC(ADPr)APAPKKG-OH

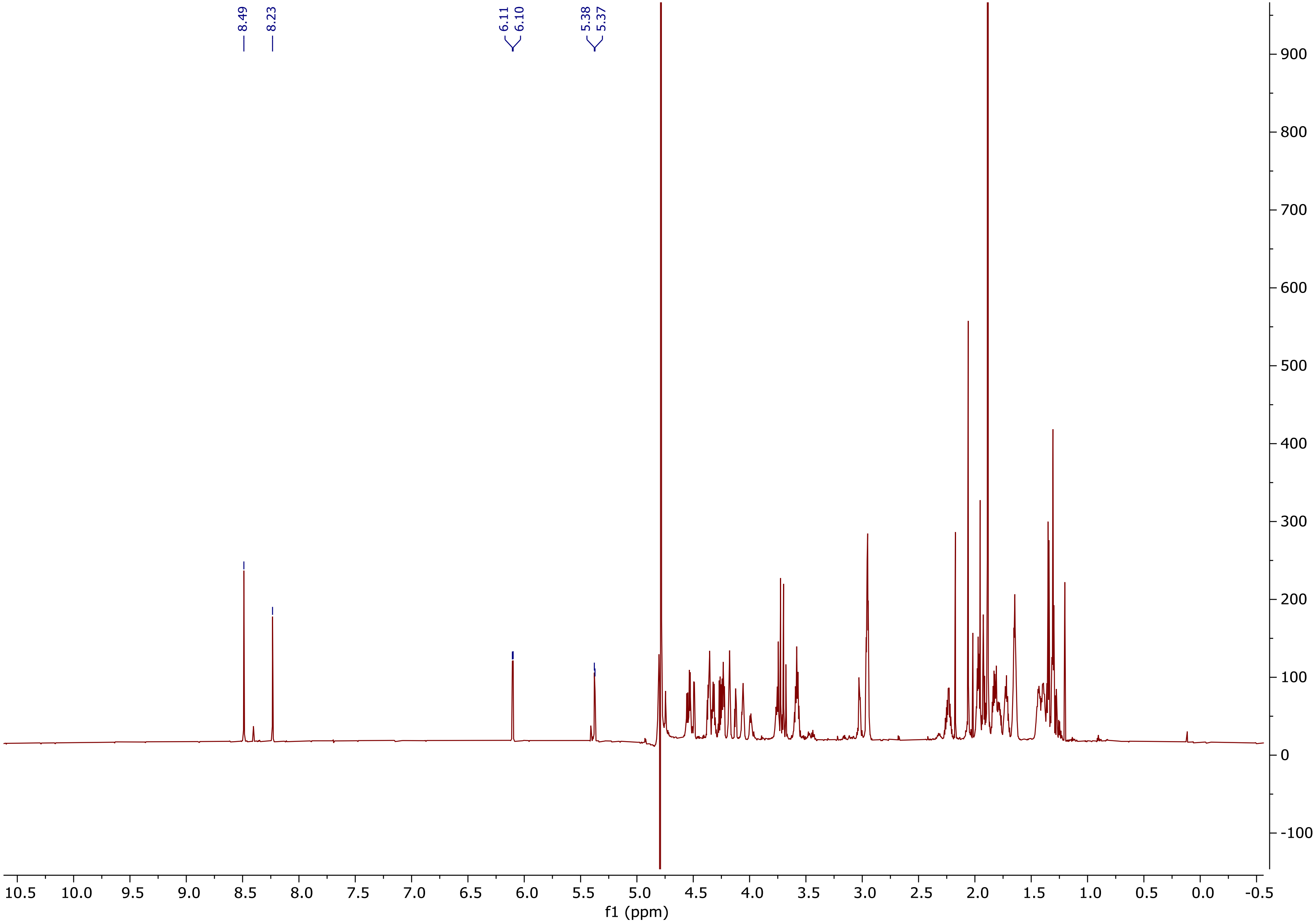

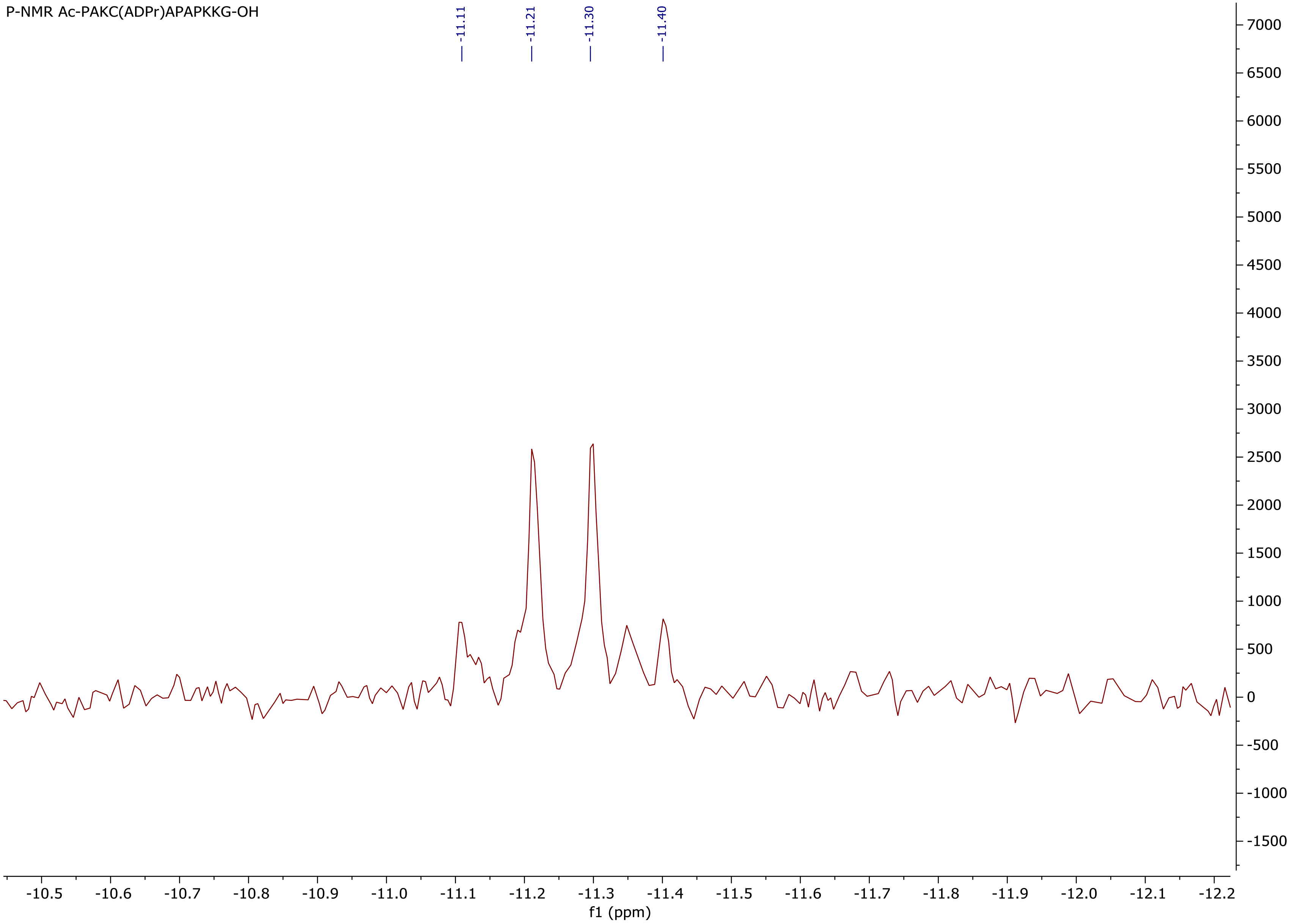
P-NMR Ac-PAKC(ADPr)APAPKKG-OH
-11.11
-11.21
-11.30
-11.40
7000
6500
6000
5500
5000
4500
4000
3500
3000
2500
2000
1500
1000
500
0
-500
-1000
-1500
-10.5
-10.6
-10.7
-10.8
-10.9
-11.0
-11.1
-11.2
-11.3
-11.4
-11.5
-11.6
-11.7
-11.8
-11.9
-12.0
-12.1
-12.2
f1 (ppm)

H-NMR biotin-PAKC(ADPr)APAPKKG-OH

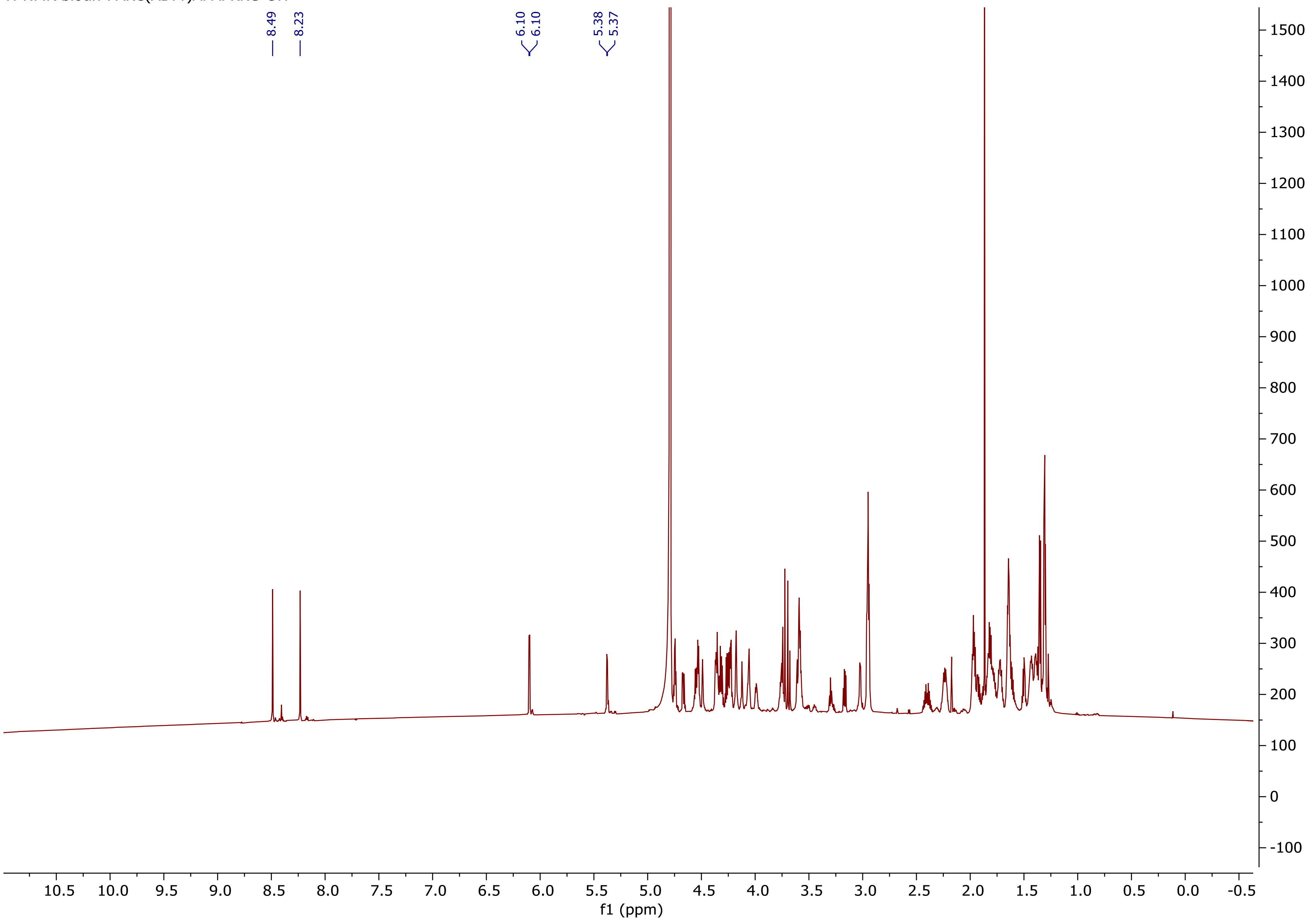

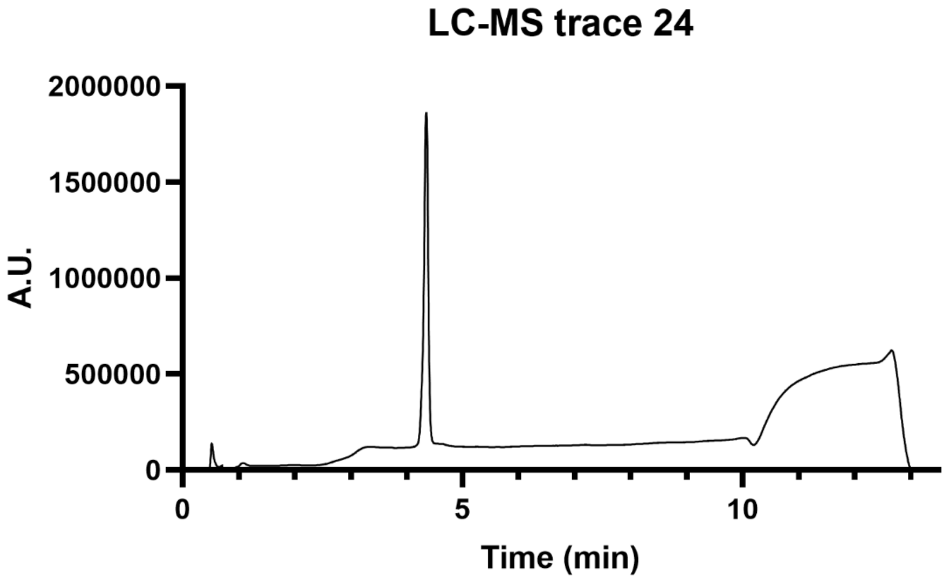
LC-MS trace 24
2000000
1500000
1000000
500000
0
A.U.
0
5
10
Time (min)

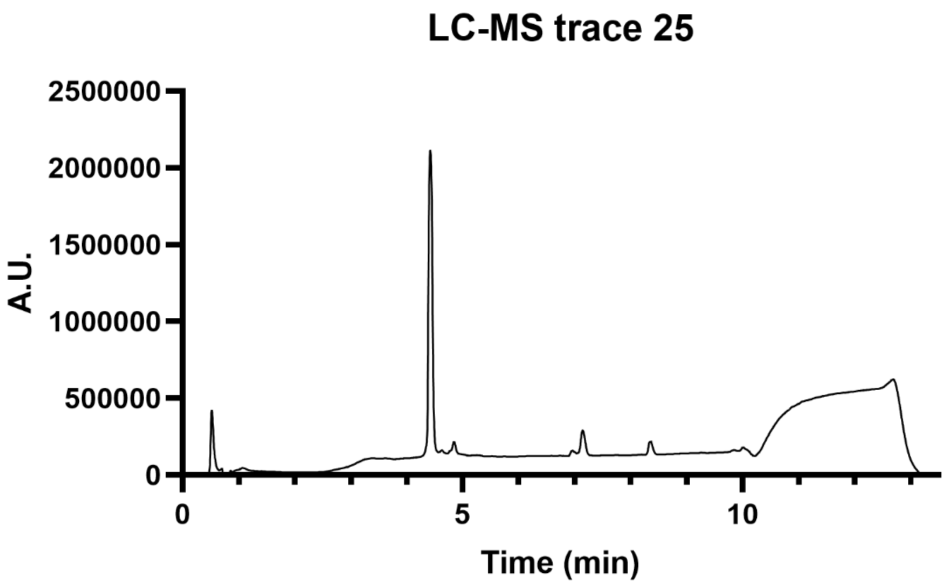

LC-MS trace 25
2500000
2000000
1500000
1000000
500000
0
A.U.
0
5
10
Time (min)

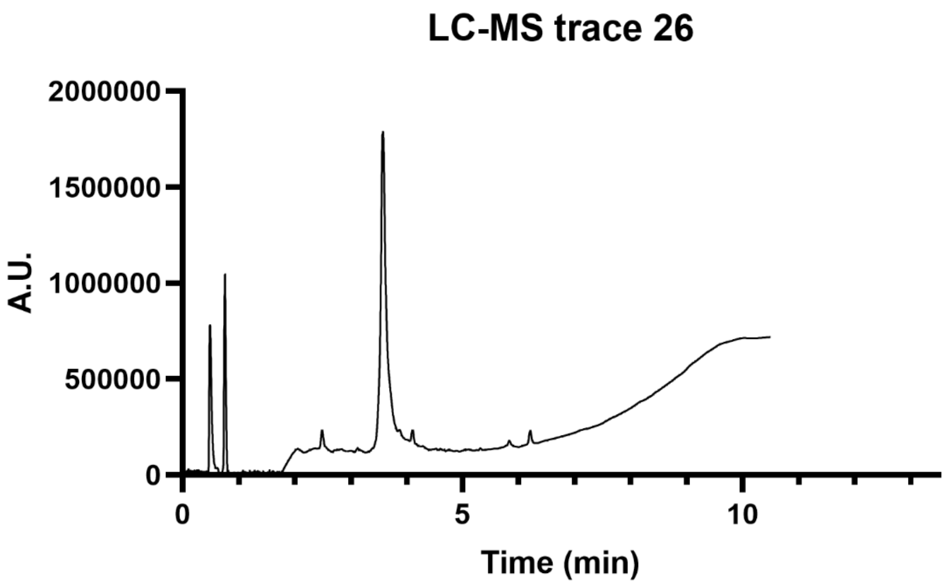
LC-MS trace 26
2000000
1500000
1000000
500000
0
A.U.
0
5
10
Time (min)

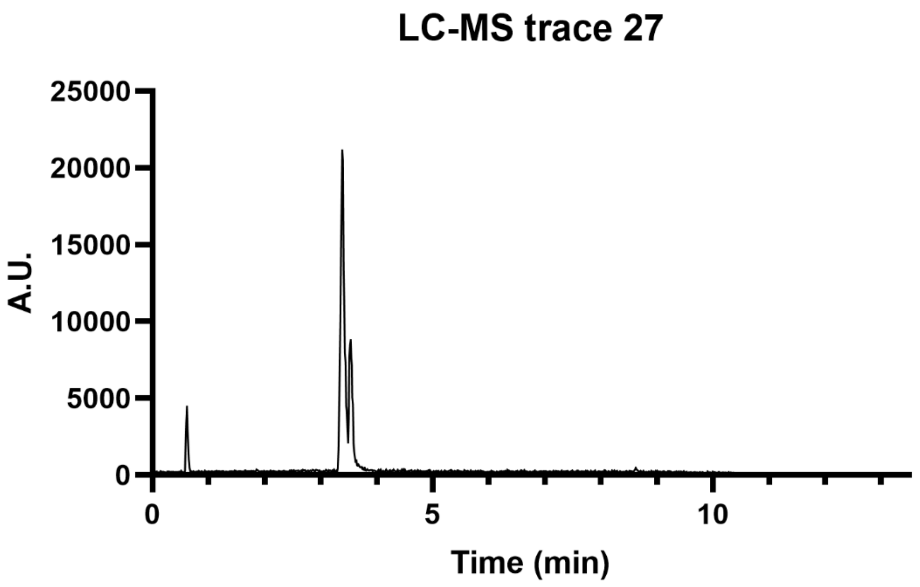
LC-MS trace 27
25000
20000
15000
10000
5000
0
A.U.
0
5
10
Time (min)

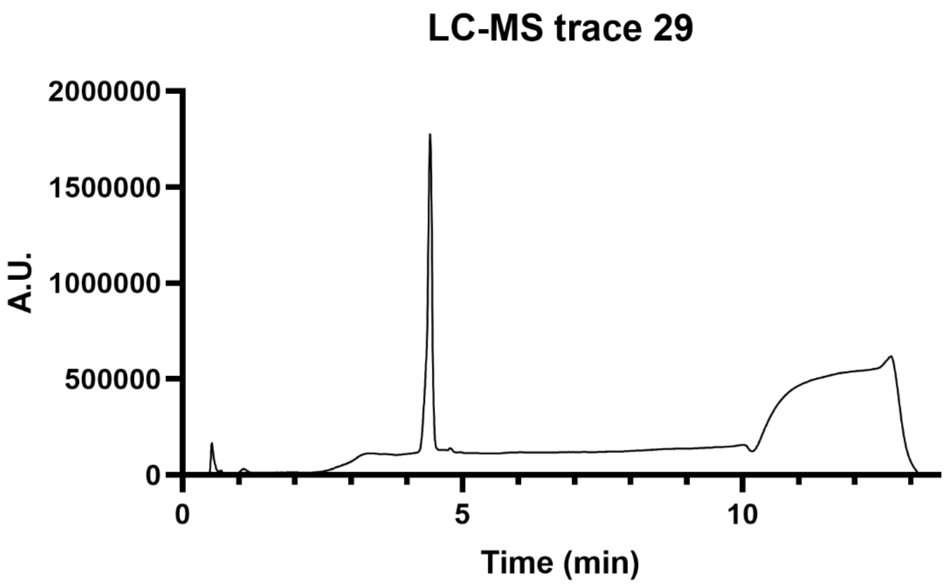
LC-MS trace 29
2000000
1500000
1000000
500000
0
A.U.
0
5
10
Time (min)

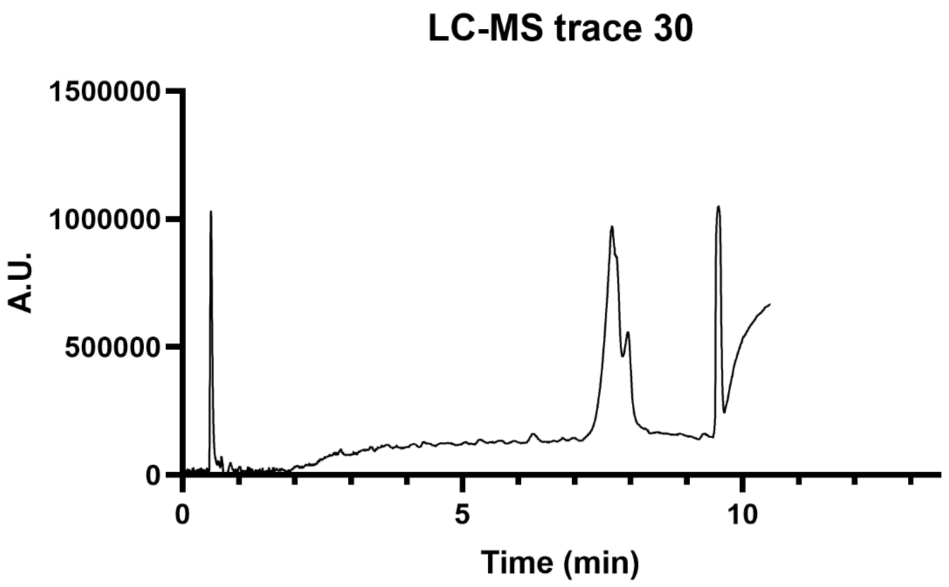
LC-MS trace 30
1500000
1000000
500000
0
A.U.
0
5
10
Time (min)
